# Losing *helena: *The extinction of a drosophila line-like element

**DOI:** 10.1186/1471-2164-9-149

**Published:** 2008-03-31

**Authors:** Rita Rebollo, Emmanuelle Lerat, Liliana Lopez Kleine, Christian Biémont, Cristina Vieira

**Affiliations:** 1 Université de Lyon; Université Lyon 1; CNRS; UMR 5558, Laboratoire de Biométrie et Biologie Evolutive, Villeurbanne F-69622, France; 2 UR477 de Biochimie Bactérienne, UR341 de Mathématiques et informatiques Appliquées, INRA. 78352 Jouy en Josas, France

## Abstract

**Background:**

Transposable elements (TEs) are major players in evolution. We know that they play an essential role in genome size determination, but we still have an incomplete understanding of the processes involved in their amplification and elimination from genomes and populations. Taking advantage of differences in the amount and distribution of the Long Interspersed Nuclear Element (LINE), *helena *in *Drosophila melanogaster *and D. *simulans*, we analyzed the DNA sequences of copies of this element in samples of various natural populations of these two species.

**Results:**

*In situ *hybridization experiments revealed that *helena *is absent from the chromosome arms of *D. melanogaster*, while it is present in the chromosome arms of *D. simulans*, which is an unusual feature for a TE in these species. Molecular analyses showed that the *helena *sequences detected in *D. melanogaster *were all deleted copies, which diverged from the canonical element. Natural populations of *D. simulans *have several copies, a few of them full-length, but most of them internally deleted.

**Conclusion:**

Overall, our data suggest that a mechanism that induces internal deletions in the *helena *sequences is active in the *D. simulans *genome.

## Background

Genome evolution occurs by several processes, including global genome duplications, segmental duplications and the amplification/deletion of repetitive sequences. Among the repeated sequences, transposable elements (TEs), which constitute a high proportion in many genomes, play an important role in genome evolution [1]. The transposition rates of these TEs depend on the amount and type of the TEs present in the genome; they are not constant over time, but are subject to amplification bursts in certain species and populations [2]. As a result, genomes contain widely differing amounts of TEs that are not directly correlated to their activity levels. For instance, the human genome is composed of at least 50% of TEs, but only very few are active, and they are responsible for less than 1% of mutations [3]. In contrast, in *Drosophila melanogaster*, only 18% of the genome is composed of TEs, but a high proportion of mutations (more than 50%) is attributable to their transposition [4].

A TE life cycle can be viewed as successive waves of transposition/loss: invasion of the host genome by TEs being followed by their progressive elimination [5,6]. For example, the *LINE-1 *(*L1*) element has colonized the entire human genome by successful waves of transposition [2,7], and today it is the most abundant TE family in this genome. However, in humans, most of the elements have been inactivated either by structural changes or by epigenetic control, such as DNA methylation [7]. In *D. melanogaster*, the *I *factor has recently reinvaded this genome after being lost from the chromosome arms [8]. TE elimination from genomes is therefore a commonly observed phenomenon, although no real-time observation of a TE extinction has ever been reported. As TEs have a considerable influence on remodeling the genome structure [9], we need to understand the dynamics of changes in their copy numbers. One way to investigate these dynamics is to analyze closely related species with differing TE amounts, such as *D. simulans *and *D. melanogaster*. These species diverged 2 to 3 million years ago [10] and have differing proportions of TEs: *D. melanogaster *contains more than 18% of TEs, whereas *D. simulans *contains only 5% [11].

D. *simulans *has fewer copies of most TEs [11], but there are a few exceptions. The DNA-transposon *hobo *is more abundant in *D. simulans*, the retrovirus-like *gypsy *and *ZAM *elements have the same low number of copies in both species, and the LINE-like element *helena *is present in the *D. simulans *genome (10 insertion sites as determined by *in situ *hybridization), but has not been detected in the chromosome arms of *D. melanogaster *[11,12]. The striking distribution of *helena *in natural populations of these two species, and the fact that degenerated copies are found in the sequenced *D. melanogaster *genome, make this LINE-like element an ideal model system to study the real time TE life cycle.

Petrov and colleagues [13] proposed that deletions are common events in Drosophila, and based this suggestion on the analysis of partial *helena *sequences from different Drosophila species. However, it is difficult to extrapolate this to other TEs, if we take into account the fact that *helena *is one of the few degenerate TEs in the *D. melanogaster *sequenced genome [14]. Comparing closely related species with differing TE amounts, could be used to test the importance of this deletion process in regulating TE genome invasions.

We analyzed the structure and activity of *helena *using the sequenced genomes and of 41 natural populations of *D. melanogaster *and *D. simulans*. We show that the elimination of *helena *from its host genomes is a very quick process, and that it is mediated by massive internal deletions in the element [15]. We conclude that the process of elimination of *helena *is far advanced in *D. melanogaster*. but is still in progress in *D. simulans*.

## Results

### *In silico *identification of a complete copy of *helena *in the *D. simulans *genome

**Figure 1:**
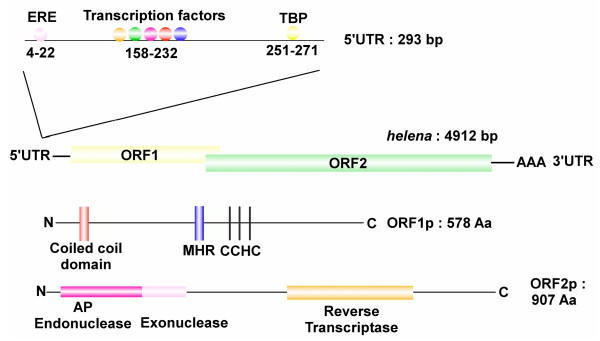
***helena *structure**. Full-length copy of *helena *in the D. *simulans *genome (3R: 1506433 – 15011368). DNA sequence: UTR, untranslated region; ORF, open reading frame; AAA, polyA tail. 5'UTR: ERE, estrogen response element; GATA, SP1, stimulating protein 1; USF-like, upstream stimulating factor-like; Protein sequences: MHR, major homology region; CCHC, cysteine rich domain; AP, apyrimidic. See Materials and Methods for prediction information.

Because no full-length copy of *helena *had previously been described, we performed a bioinformatic search for such a copy in the draft sequence of the *D. simulans *genome [16]. We found a 4912-bp copy of *helena *on the chromosome arm 3R (at position 1506433 – 1511368 on the minus strand) (Figure 1). *Helena *belongs to the *jockey *clade [17], has a 25-bp poly A tail, and two overlapping open reading frames (ORF1 and ORF2). The first ORF is 1737-bp and codes a 579-amino acid (aa) protein that has high similarities to the *gag *protein of other LINE-like elements, such as *X*, *jockey *and *HeT-A *[18,19]. The *gag*-like protein contains the major homology region (MHR), followed by a cysteine-rich domain (CX_2_CX_4_HX_4_C, CX_2_CX_3_HX_4_C, CX_2_CX_3_HX_6_C). This region is common to all *gag*-like proteins, and confers an RNA or DNA single-strain binding property on these elements, as well as being essential for *gag *oligomerization. *Helena *has a coiled-coil domain located in the 5' region of the *gag *protein, something that had previously only been seen in L1 elements from mammals [20] and in some LTR retrotransposons from Drosophila [21]. The second ORF, which starts on the last base of ORF1, is 2721-bp, and codes a 907-aa protein corresponding to the *pol *gene, which is very similar to the protein of the *BS *and *jockey *elements. The *pol*-like protein contains all the domains necessary for its function: an apyrimidic endonuclease and an exonuclease (from amino acid 4 to 221), plus a reverse transcriptase domain (from amino acid 493 to 746). Both ORFs are intact, could produce transcripts, and are surrounded by two untranslated regions (5'UTR and 3'UTR respectively). Because the regulatory region is often defined in the 5'UTR [22], we performed a bioinformatic search for transcription factors binding sites in this region. A single region was detected containing several transcription factor binding sites, such as SP1 and upstream stimulating factor-like (USF) binding domains. This region also displays a binding site for a TATA-binding protein (TBP), and an estrogen response element (ERE).

### The copies of *helena *in the sequenced genomes of *D. melanogaster *and D. *simulans*

Using the complete sequence of *helena *as a query, we found 62 *helena *sequences in the *D. simulans *genome (see Additional File 1 for details). Twenty-eight of these copies were located on the chromosome arms, and the remaining 34 were in the U part of the genome, that may correspond to heterochromatin. The copies ranged in size from 107 bp to 5098 bp. However, it is difficult to determine the exact size of some copies due to the presence of numerous undetermined bases. Two copies (chr2R_13305831 and chrX_16602314) were longer than the reference sequence due to insertions. In some other cases, we may be looking at fragments of the same copy; however, the distances that separate them are too large to allow us to find out with certainty whether they come from the same copy. The estimated number of 62 copies in *D. simulans *may therefore be an overestimation. The average percentage identity is 96.1% for all copies, with an average of 97.4% for the copies in the euchromatin, and of 94.9% for the copies in the U part.

In the sequenced genome of *D. melanogaster*, we found 26 copies of *helena *(see Additional File 2 for details), which ranged in size from 91 bp to 4805 bp. The average percentage identity was 80.4% for all copies, with an average of 78.7% for the copies in the chromosome arms, and of 83.7% for the copies located in the U part. Most of the copies in this genome have therefore been degraded, with numerous internal deletions or insertions. All copies are truncated on the 5' side, and are DOA (Dead on arrival) copies, apart from the 3L_23487977 copy, although even this displays some internal deletions.

**Figure 2:**
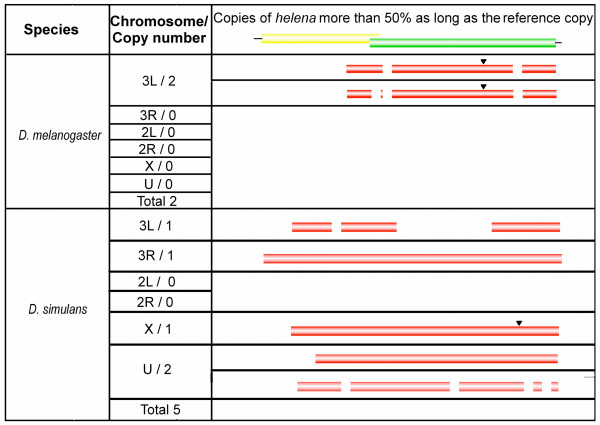
**Scheme of helena copies**. Representation of *helena *copies in the D. *melanogaster *and D. *simulans *genomes with at least 90% identity and 50% of the length of the complete copy, and with e-values of less than 10e^-10^; Triangles = insertions; Spaces = deletions.

We analyzed in greater detail any copies that could correspond to the most recent insertions in both *D. melanogaster *and *D. simulans*. We used specific blast criteria to identify these copies: we selected matches with at least 90% identity, and a length at least 50% of that of the complete copy, with e-values of less than 10e-10 (Figure 2). In *D. melanogaster*, only the 3L_23487977 copy described above met all the blast criteria. It is obviously an inactive copy, since more than four deletions were detected within its sequence. In *D. simulans*, six copies were found that matched the blast criteria, including the complete copy on the 3R chromosome (position 1506433 – 1511368 on the minus strand); the other five copies had internal deletions, and insertions were detected in four of them.

### Chromatin localization of *helena *copies in natural populations

We used *in situ *hybridization to estimate the number of *helena *insertion sites located on the arms (euchromatin) of the polytene chromosomes from salivary glands of both *D. melanogaster *and *D. simulans*. Both species had centromeric staining, but only D. *simulans *from natural populations presented euchromatic bands (mean copy number 10.7 ± 2.2) with no fixed sites (see Additional File 3 for details on insertion sites per population). With this experiment we did not detect any insertions of *helena *in the chromosome arms, which could be explained by the short size of the elements and the divergence to the probe used.

#### Inter-population polymorphism

**Figure 3:**
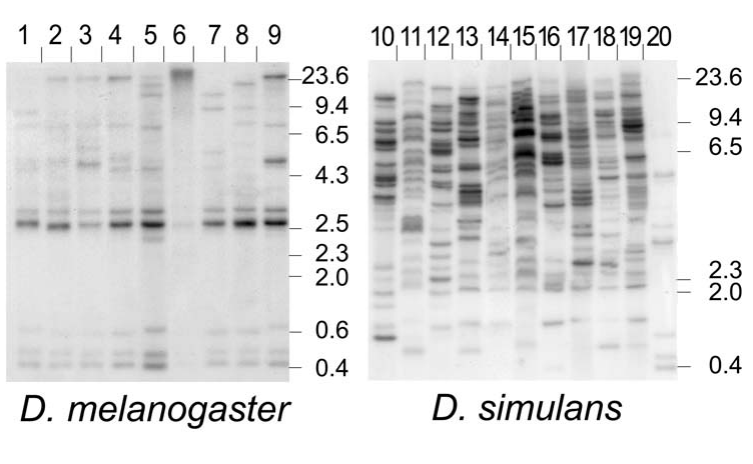
**Southern blot analysis of D. *melanogaster *and D. *simulans *populations**. Lanes 1 to 9 are D. *melanogaster *populations (Bolivia, Brazzaville, Canton, Chicharo, Reunion Island, Arabia, Virasoro, Vietnam, and ISO for the 9^th ^and 20^th ^lane). Lanes 1 to 19 are D. *simulans *populations (Amieu, Eden, Valence, Canberra, Papeete, Moscow, Makindu, Zimbabwe, Cann River and Reunion Island). For both Southern blots, the DNA size is estimated in base pairs.

We analyzed the inter-population *helena *copy number polymorphism by Southern blot, using a restriction enzyme that does not cut inside the element. This method detects both heterochromatic and euchromatic sequences. As shown in Figure 3, *D. melanogaster *had 8 to 11 bands per population, and several bands were shared by different populations. These copies could correspond to ancient and fixed heterochromatic copies in the *D. melanogaster *genome. *D. simulans *populations contained numerous *helena *copies (19 to 30 copies per population) with a high level of insertion polymorphism. Since the enzyme used for the Southern blot did not cut inside the element, all bands over 4.5 kb could correspond to a complete element. Because both species harbored bands over 4.5 kb, they could have full-length *helena *copies.

#### PCR screening

Three sets of primers were used to amplify the whole ORF1 and two fragments of the ORF2. No bands corresponding to the ORF1 were observed in any of the *D. melanogaster *natural populations in agreement with the absence of this ORF in the sequenced genome. There was a high level of size polymorphism for the *D. melanogaster *ORF2, corresponding to the different internal deletions already analyzed by Petrov et al. [13,23]. In contrast, *D. simulans *displayed low size polymorphism for *helena *ORF1 and ORF2. Indeed, only one population out of twenty had two sets of ORF1 (Papeete). All *D. simulans *populations had two to three sets of ORF2.

#### Analysis of *helena *copies in D. *simulans *populations

**Figure 4:**
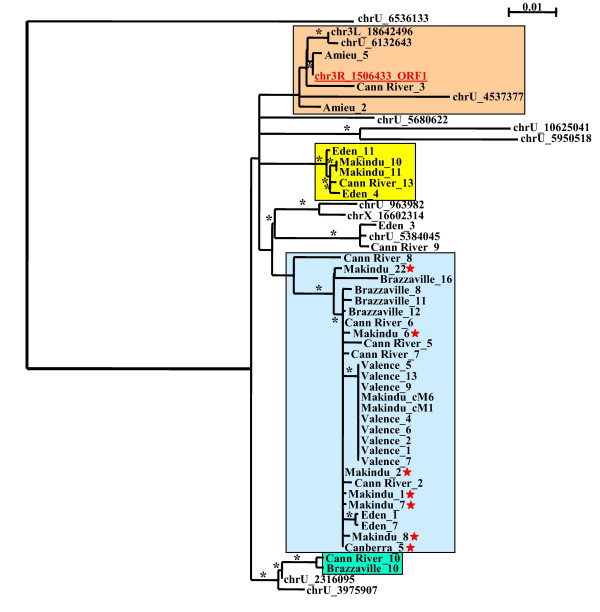
**phylogenetic tree of the DNA sequences from the ORF1 region**. The reconstruction was performed on the cloned DNA sequences of the ORF1 region from the different populations of *D. simulans*, and from some sequences detected in the sequenced genome (we eliminated sequences that were too short relative to the global length of the alignment). Colored boxes identify sequences harboring common patterns of deletions and/or insertions. All the positions are given by reference to the complete copy. Green box: sequences display the same deletions of 118 bp (at position 45), 3 bp (at position 839), 6 bp (at position 846), 1 bp (at position 854) and a 1-bp insertion (at position 415). Blue box: sequences display the same 28-bp deletion (at position 1092) – those with a red star also have a 77-bp deletion (at position 508), and a 91-bp deletion (at position 593). Yellow box: sequences display the same deletions of 1 bp (at position 160), 28 bp (at position 165), 19 bp (at position 954), 2 bp (at position 989), and 37 bp (at position 1006), and an insertion of 1 bp (at position 322). Orange box: sequences with no deletion or insertion, very closely related to the complete copy chr3R_1506433. Black asterisks correspond to bootstrap values greater than 50%.

**Figure 5:**
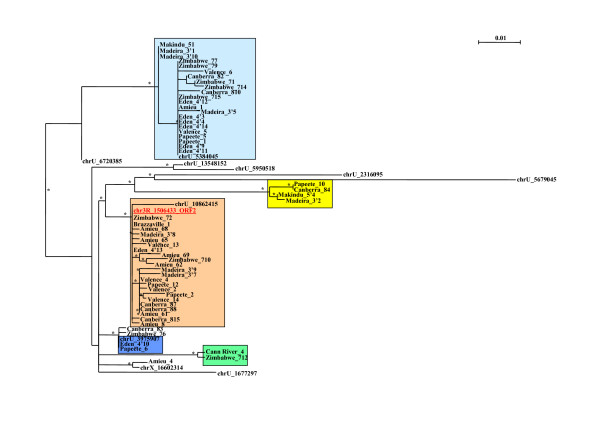
**phylogenetic tree of the DNA sequences from the ORF2 region**. The reconstruction was performed on the cloned DNA sequences of the ORF2 region from the different populations of *D. simulans *and from some sequences detected in the sequenced genome (we eliminated sequences that were too short relative to the global length of the alignment). Colored boxes represent sequences harboring common patterns of deletions and/or insertions. All the positions are given relative to the complete copy. Green box:sequences display the same 4-bp deletion (at position 547) and the same 2-bp insertion (at position 112). Dark blue box: sequences display the same 1-bp deletion (at position 521). Yellow box: sequences display the same deletions of 3 bp (at position 143), 8 bp (at position 335), 4 bp (at position 345), and an insertion of 2 bp (at position 460). Light blue box: sequences display the same 401-bp deletion (at position 179). Orange box: sequences with no deletion or insertion, very closely related to the complete copy chr3R_1506433. Black asterisks correspond to bootstrap values greater than 50%.

PCR fragments obtained from the *D. simulans *population screening mentioned above were cloned and sequenced. Surprisingly several common indels were detected at the same positions in different sequences from all the populations analyzed. Phylogenetic reconstructions based on the ORF1 and ORF2 (Figures 4 and 5, alignments from Additional Files 4 and 5) showed that the sequences that displayed the same indels are grouped in the tree. This suggests that the deletion or insertion events were produced before the amplification of these sequences. Some copies had only a few insertions, and might be inactive since their reading frames were not preserved. However, the amplification of some of these copies could have been promoted in trans. We did not find any population that had both complete ORFs. Nevertheless, some ORFs had no internal stop codons in their sequence, suggesting that copies bearing them could be active despite the deletions.

The percentage identity between the reference copy and ORF1 ranges between 96% and 99%, meaning that these copies have not diverged much. No relationship was detected between the size and location of the deletions, and the percentage identity. For the ORF2, we found copies with a percentage identity of more than 93% that reached 100% for some copies. A common 401-bp deletion was found in copies with 93% identity with the complete *helena *copy, but no correlation was observed with the percentage identity for the other deletions or insertions. Based on the age estimation of each copy, we found that most of the oldest ORF2 fragments had the 401-bp deletion. Several young copies of both ORF1 and ORF2 displayed major internal deletions, showing that the mechanism leading to these deletions is much more powerful than copy divergence in inactivating them.

#### Transcript analysis in D. *simulans*

To test the transcriptional potential of *helena *in *D. simulans*, we performed northern blot and RT-PCR in various populations (see Materials and Methods). Since the sequenced genome was obtained from strains from North America, we added three populations from this continent (San Antonio, SW3-S2 and San Diego). No transcripts were found by Northern blot in any of the populations. However, the RT-PCR method detected transcripts of both ORF1 and ORF2 in the Valence population, an extremely low signal for ORF1 and ORF2 in three of the American populations, and ORF2 transcripts in the Amieu population. None of the other populations had any transcript for *helena*, implying that this element is extinct in these populations. Since the Northern blot technique is less sensitive than RT-PCR, these findings suggest that *helena *is transcribed at extremely low levels in the populations in which some transcripts were detected by RT-PCR.

## Discussion

Our *in silico *and experimental analyses of the *D. melanogaster *genome show that *helena *copies are mostly DOA, devoid of ORF1, and therefore unable to transpose autonomously. All these features have been associated with elements that are in the process of inactivation [20]. The scenario for the *helena *copies in *D. simulans *is quite different. Analysis of the sequenced genome of this species allowed us to identify a full-length copy of *helena *with the structures required for an active element: two intact ORFs, a poly-A tail, and regulatory regions. The high level of insertion polymorphism detected in the natural populations suggests that *helena *is an active element or has been active recently. However, sequence analysis of the two ORFs of *helena *in the natural populations revealed two main points: **first**, both ORFs are intact in only very few populations; **second**, even though the sequences of *helena *are very similar at the nucleotide level, their deletion features differ.

The first point was strengthened by the almost total absence of *helena *transcripts in all natural populations of *D. simulans*, which means that very few copies were involved in transcription. Because a single master copy is enough to maintain TE transposition [24], the putative activity of *helena *in this species could reside in the full-length copy probably present in some populations such as Valence, where we did observe transcripts. Hence, we would expect sequences that are still similar at nucleotide level to differ only in the 5' end truncation size, as usually observed for LINE-like elements. However, as mentioned in the second point, we actually observed many other kinds of internal deletions that occurred throughout the length of the element. Intriguingly this deletion-promoting process appears to be quite powerful in inactivating the elements, and could be even more powerful than other mutation processes such as point mutations. This means that a real-time loss of *helena *is ongoing in all *D. simulans *populations.

The nature of the mechanisms leading to internally deleted copies is still unknown. In humans, LINE elements can be spliced [25], a process that creates internal deletions. We used bioinformatic analyses, but were unable to find splice sites in the full-length *helena *sequence. Also, although recombination between mRNAs can produce internal deletions [26], *helena *sequences are not sufficiently divergent to allow us to infer the origin of a single copy.

*Helena *appears to be extinct in *D. melanogaster*, and this recalls the *I *element, which also disappeared from the *D. melanogaster *genome in the past, and reinvaded it only recently [27]. The *I *and *helena *elements are both LINE-like elements, leading us to wonder whether amplification/loss of copies could be a characteristic of this type of element. Waves of amplification/loss have been observed in humans, where only the youngest L1 subfamily is active, perhaps as a result of competition between different L1 subfamilies [2,7].

Our data show that *helena *has been almost entirely removed from the *D. melanogaster *genome, and was not subjected to the recent wave of transpositions reported for other elements [14]. In *D. simulans*, we did observe an insertion site polymorphism of *helena*, but this corresponds to copies that are being internally deleted by an efficient mechanism. This may be generalized on the basis of data on the LTR retrotransposons *412 *and *tirant*, which have also internal deletions [28,29]. We still do not clearly understand which mechanisms lead to a low copy number in the *D. simulans *genome, but a mechanism promoting internal deletions could be a major force at work [30].

## Conclusion

TEs are major players in genome evolution, and the way they are controlled by the host genome is one of the most fundamental questions in evolutionary genomics. Here we show that two closely related species of drosophila have a TE family at different stages in its life cycle. The mechanism by which this is achieved in *D. simulans *implies that a very efficient internal deletion mechanism is acting on TEs, which is more powerful than the simple neutral evolution of non-active elements. The difference in the amount of TEs between *D. melanogaster *and *D. simulans *could be explained by such a process, that doesn't seem to be very active in *D. melanogaster *present populations.

## Methods

### Natural populations

We worked on fly samples collected from several geographically-distinct, natural populations (confer each Method for natural populations investigated). These populations were maintained in the laboratory as isofemale lines or small mass cultures with around 50 pairs in each generation.

### *In situ *hybridization

Polytene chromosomes from salivary glands of third-instar female larvae were prepared and treated with nick-translated, biotinylated DNA probes, as previously described [31]. Insertion sites were visible as brown bands resulting from a dye-coupled reaction with peroxidase substrate and diaminobenzidine. The insertion site numbers of the TE(s) were determined on all the long chromosomes arms (X, 2L, 2R, 3L, 3R), and were summed to give the total number of labeled sites per diploid genome. We did not take into account the insertions located in pericentromeric regions 20, 40, 41, 80, and 81, because TE site number estimations in these regions are difficult and not reliable for all chromosomes or all squashes. We used a probe (1278 bp) from *helena *of *D. sechellia *(AF012044). The following populations of *D. melanogaster *were investigated: Portugal (Chicharo), Saudi Arabia, Congo (Brazzaville), Reunion Island, Argentina (Virasoro), Bolivia, China (Canton), Vietnam, and Iso line. The *D. simulans *populations analyzed were from France (Valence), Russia (Moscow), Kenya (Makindu), Zimbabwe, Reunion Island, Australia (Eden, Cann River and Canberra), French Polynesia (Papeete), New Caledonia (Amieu).

### Southern blot

DNA was extracted from one or five adult females by a standard phenol-chlorophorm-salt method with proteinase K digestion. The *D. melanogaster *populations analyzed were from Bolivia, Congo (Brazzaville), China (Canton), Portugal (Chicharo), Reunion Island, Saudi Arabia, Argentina (Virasoro), Vietnam, and ISO line. The *D. simulans *populations analyzed were from New Caledonia (Amieu), Australia (Eden, Cann River and Canberra), France (Valence), French Polynesia (Papeete), Russia (Moscow), Kenya (Makindu), Zimbabwe, and Reunion Island. The DNA was cut using the *Hind*III enzyme, which has no restriction site within the *helena *sequence, and therefore allowed us to estimate the number of complete *helena *copies. Electrophoresis of a 0.8% agarose gel containing digested DNA was carried out for 17 h. The DNA was denatured (NaOH 0.5 M), and then transferred overnight to a Hybond-N+ nylon membrane. Pre-hybridization and hybridization were carried out at 67°C using a Denhardt 5× solution. The probe used for hybridization (AF012044) was radiolabeled with ^32^P, using a random procedure from Amersham.

### Amplification of ORF1 and ORF2

DNA was extracted from single flies by a standard phenol-chlorophorm method. The following populations of *D. melanogaster *were investigated: France (Valence and Saint Cyprien), Portugal (Chicharo), Saudi Arabia, Senegal, Congo (Brazzaville), Reunion Island, Guadeloupe, Argentina (Virasoro), Bolivia, and China (Canton). The *D. simulans *populations analyzed were from France (Valence), Russia (Moscow), Egypt (Tanta), Congo (Brazzaville), Kenya (Meru, Kwalé and Makindu), Zimbabwe, Tanzania (Arusha), Puerto Rico, Japan, Australia (Eden, Cann River and Canberra), French Polynesia (Papeete), Saint Martin, Hawaii, New Caledonia (Amieu), and Portugal (Madeira).

PCR was run using 1 μg DNA with the two following primers – ORF1: H1for (285 5' AAC TGT AAA ATG GAT ACG AAC A 3' 306), H1rev (1808 5' GCC ACT TCA TAA ATT GTT CC 3' 1827). – ORF2: Hel2F (2325 5' CCG GGC TGG GCG ATA TGG 3' 2342), Hel2R (4548 CGT ACA TAC CAG GGG CAG TTG G 3' 4569). PCR was run in 30 cycles with annealing temperatures of 57°C (ORF1) and 56°C (ORF2). We used Euroblue taq from Eurobio. DNA amplified fragments were purified and cloned on competent bacteria (Qiagen kits). Four primers were used for sequencing: M13 forward and reverse; Seq1 (5' CTC TTC CTT CAT TTG GTA CG 3') and Seq2 (5' AAG GGG AAA CAG TGA GAA TA 3') for the complete ORF1; Seq3F (5' TTA GAC CAT GCT CTC GGT TA 3') and Seq3R (5' TGT CAA TTC CTG GAG CTT TA 3') for a fragment of ORF2. Sequencing was performed by Genome Express. Accession numbers (Genbank) from EU168807 to EU168844 correspond to ORF1 fragments. Accession numbers (Genbank) from EU170377 to EU170431 correspond to ORF2 fragments.

### RT-PCR

Total RNA was extracted from four adult females, four adult males, 10 ovaries and 10 testes from *D. simulans *populations (France (Valence), Congo (Brazzaville), Kenya (Makindu), Zimbabwe, Australia (Canberra), New Caledonia (Amieu), Portugal (Madeira), United States (San Antonio, San Diego, Arena, SW3)) with the RNeasy protect mini kit from Qiagen. RNA extracts were treated with the Ambion's DNA-free kit. ThermoScript RT-PCR system from Invitrogen was used to synthesize four different cDNA pools (55°C for 90 min and 85°C for 5 min): a control reaction with no retrotranscriptase to test DNA contamination, a pool of total cDNA synthesized with oligo-dt primers, two specific cDNA pools obtained with H1R (ORF1) and Hel2R (ORF2), respectively, corresponding to *helena *transcripts. All four cDNA pools were tested for the presence of *actin *cDNA (house keeping gene) by PCR with Act5cfw (5'ATGTGACGAAGAAGTTG3') and Act5cRv (5'TTAGAAGCACTTGCGGTGCA3') primers. Oligo-dt and specific *helena *cDNA pools were analyzed by PCR using ORF1 and ORF2 specific primers (H1R/H1F and Hel2R/Hel2F).

### Northern blot

Total RNA was extracted from adult females or embryos from several *D. simulans *populations (Valence, Makindu, Amieu, Brazzaville) with the RNeasy protect mini kit from Qiagen. Total RNA extracts were treated with the Ambion's DNA-free kit. Electrophoresis (MOPS, formaldehyde gel) was run for 3 h after RNA denaturing. After washing (water and NaOH, 75 mM) RNA was passively transferred to a nylon membrane, and cross linked for 2 hours at 64°C. Blots were pre-hybridized in hybridization buffer, then hybridized overnight at 42°C in hybridization buffer containing a ^32^P-labeled *helena *cDNA probe. The radiolabeled cDNA probe was prepared using a Megaprime DNA Labeling Kit according to the manufacturer's protocol (Cat # RPN 1607; Amersham Biosciences, Little Chalfont, Buckinghamshire, England). Following hybridization, blots were washed in 2 × SSC/0.1% SDS at 42°C and then exposed to X-ray film (KODAK).

### Identification of *helena *copies in the complete genomes

We retrieved the sequences of the chromosome arms 2L, 2R, 3L, 3R, 4, X and the unassigned part (named U) corresponding to the first release of the mosaic assembly of the genome of *D. simulans *available at the ftp site of the Genome Sequencing Center at the Washington University Medical School [32]. This mosaic assembly corresponds to different strains of *D. simulans*. We also used the sequenced genome of *D. melanogaster *[33]. We will refer to the *helena *copies found in the genomes according to the chromosome name and the start position of the copy (for example chr2L_133500 corresponds to a copy found on the 2L chromosome, and it starts at position 133500).

The *helena *element was only found in the databases as fragments of the reverse transcriptase (RT). We retrieved the longest sequences from D. *yakub*a (accession number in Genbank AF012049), *D. melanogaster *(AF012030) and *D. virilis *(U26847) to build a chimeric, 1532-bp sequence. Using this chimeric element we searched for copies in the *D. simulans *sequenced genome using blastn [34]. The reconstructed *helena *sequence (ID Helena_DS) is available in Repbase [35]. Only matches with an e-value of less than 10e^-10 ^have been retained, and any separated by distances of less than 300 bp have been merged. As the query used corresponds to a small portion of the ORF2, in order to search for longer sequences of *helena*, we retrieved the matches after adding 5000 bp around their positions. We then performed multiple alignments of these sequences using clustalw [36] in order to detect the longest copies. By this procedure, we identified a sequence on the chromosome 3R that was the longest of the matches detected. The prediction of potentially coding parts was made using the ORF finder program available on the NCBI web site [37], and this allowed us to identify two ORFs. It was not possible to use the presence of target site duplication to determine the exact position of the beginning of the sequence, because the copy was surrounded by unidentified bases, and so we performed a blast search in the draft sequence of *D. sechellia*, the closest relative to *D. simulans*. This allowed us to find a homologous copy, and to identify the beginning of the complete copy of *helena*. Once this copy had been identified, it was used as a query to perform blast searches in the *D. simulans *and *D. melanogaster *genomes to determine the *helena *copy populations.

### Sequence analysis

The computation of the percentage identity was performed using the DNADIST program in the PHYLIP package [38]. We used the sequence editor Seaview [39] to visualize the sequences and the alignments. Splice sites and transcription binding sites were predicted by the Softberry tools [40] and Genomatix [41]; PEPcoil ([42] allowed us to find the coiled coil domain in ORF 1. Conserved domains in both ORF1 and ORF2 were predicted with the "Conserved domain search" tool from NCBI. Sequenced copies were aligned with T_coffee [43]. Phylogenetic analysis were made using maximum likelihood with HKY substitution model implemented in PhyML [44]. The reconstruction was performed on the cloned DNA sequences of the ORF1 and ORF2 region from the different populations of *D. simulans*, and from some sequences detected in the sequenced genome (we eliminated sequences that were too short relative to the global length of the alignment).

Age was estimated using the Bowen and McDonald method [45] with the formula Age = K/(2r), where K is the divergence between the two copies calculated from the Kimura two-parameter distance via DNAdist, and r is the synonymous substitution rate per site per million years in *D. melanogaster *(r = 0.016 from Li [46]). It is important to note that the age of *helena *copies is underestimated due to the lack of knowledge about conversion and substitution rates in *D. melanogaster *genome, and is also unreliable when applied to old and highly diverged copies.

## Abbreviations

DOA, dead on arrival; LINE, long interspersed nuclear element; L1, LINE 1; LTR, long terminal repeat; ORF, open reading frame; TE, transposable element

## Authors' contributions

RR and LL carried out the molecular and genetic studies, RR and EL did the bioinformatic analysis, CV and CB contributed to the design of the study, CV designed and coordinated the study. All the authors contributed to writing of the paper. All the authors have read and approved the final manuscript.

## Supplementary Material

Additional File 1 *helena *copies in the *Drosophila simulans *sequenced genome. The data provided is a list of the *D. simulans *copies Click here for file

Additional File 2 *helena *copies in the *Drosophila melanogaster *sequenced genome. The data provided is a list of the *D. melanogaster *copies. Click here for file

Additional File 3 Helena copy number in *D. melanogaster *and *D. simulans *populations by in situ hybridization in polytene chromosoms. The data provided de in situ hybridization results. Click here for file

Additional File 4 Alignment of helena ORF1. The data provided the alignement used to construct the tree on Figure 4 Click here for file

Additional File 5 Alignment of helena ORF2. The data provided the alignement used to construct the tree on Figure 5 Click here for file
