# Supplementary material for: Losing helena: The extinction of a drosophila line-like element
Source: BMC Genomics. 2008 Mar 31;9:149. doi: 10.1186/1471-2164-9-149 (PMC2330053; doi:10.1186/1471-2164-9-149)
Supplement: Additional File 1 — helena copies in the Drosophila simulans sequenced genome. The data provided is a list of the D. simulans copies [file 1471-2164-9-149-S1.doc]

Supplementary Table 1: *helena* copies in the *Drosophila simulans* sequenced genome

| **chromosome** | **strand** | **start** | **stop** | **length (bp)** | **%identity with the complete *helena*** |
| --- | --- | --- | --- | --- | --- |
| 2L^£^ | + | 1592410 | 1592944 | 534 | 99.25 |
| 2L^¥^ | + | 1674490 | 1674844 | 255 | 99.21 |
| 2L^*^ | - | 15248615 | 15252309 | 3680 | 99.75 |
| 2L^£^ | + | 17606797 | 17606986 | 190 | 100.00 |
| 2L^£¥^ | + | 21324887 | 21324994 | 108 | 90.74 |
| 2L^£^ | - | 21614564 | 21615205 | 642 | 99.22 |
| 2L^£^ | + | 21940141 | 21940640 | 500 | 96.59 |
| 2R^£^ | - | 1707808 | 1707920 | 113 | 97.32 |
| 2R^£^ | - | 11963548 | 11964592 | 1045 | 99.81 |
| 2R^£^ | - | 13305831 | 13310157 | 4327 | 99.22 |
| 2R^£^ | - | 14875310 | 14876458 | 1149 | 99.91 |
| 2R^¥^ | - | 14892514 | 14892896 | 383 | 98.35 |
| 2R^£^ | - | 15489919 | 15490045 | 128 | 96.06 |
| 3L^£^ | + | 6812601 | 6816422 | 3821 | 99.63 |
| 3L^£^ | - | 13781457 | 13782139 | 683 | 99.56 |
| 3L^£^ | - | 14741536 | 14741739 | 204 | 99.51 |
| 3L^£^ | - | 18635059 | 18635811 | 753 | 99.07 |
| 3L^¥^ | - | 18642496 | 18643290 | 795 | 100.00 |
| 3L^£^ | + | 22376004 | 22376483 | 479 | 100.00 |
| 3R^£¥^ | - | 30161 | 30267 | 107 | 88.79 |
| 3R^£^ | + | 5983652 | 5983777 | 126 | 95.20 |
| 3R^£^ | - | 1504547 | 1505432 | 885 | 99.77 |
| **3R^§^** | **-** | **1506433** | **1511368** | **4912** | **/** |
| 3R^£^ | + | 17102251 | 17102891 | 641 | 99.53 |
| 3R^£^ | + | 18466755 | 18467294 | 539 | 99.81 |
| X^£^ | + | 4098908 | 4099039 | 131 | 96.92 |
| X^£¥^ | - | 16594484 | 16595282 | 799 | 93.59 |
| X^£*^ | + | 16602314 | 16610995 | 8682 | 96.94 |
| X^£^ | - | 16622045 | 16623141 | 1096 | 95.62 |
| U^£^ | + | 233264 | 234099 | 835 | 95.08 |
| U^¥$^ | - | 963982 | 966622 | 2641 | 97.01 |
| U^£^ | + | 1677297 | 1679692 | 2396 | 96.16 |
| U^¥^ | - | 1817942 | 1818183 | 242 | 96.28 |
| U^£$^ | + | 2316095 | 2319355 | 3260 | 94.32 |
| U^£¥^ | + | 3850078 | 3850295 | 218 | 98.17 |
| U^$^ | + | 3975907 | 3980988 | 5098 | 97.12 |
| U^£^ | - | 4355937 | 4357004 | 1068 | 94.10 |
| U^£¥^ | + | 4537377 | 4539308 | 1932 | 96.83 |
| U^£^ | - | 5367908 | 5368352 | 444 | 99.55 |
| U^£¥*^ | + | 5384045 | 5387314 | 3270 | 96.16 |
| U^£¥^ | + | 5494730 | 5495329 | 600 | 95.67 |
| U^£^ | - | 5537882 | 5538331 | 449 | 95.54 |
| U^£¥$^ | - | 5679045 | 5679704 | 660 | 91.92 |
| U^£¥^ | - | 5680622 | 5681500 | 879 | 95.56 |
| U^£¥$^ | + | 5950518 | 5951775 | 1258 | 93.13 |
| U^£¥^ | - | 6132643 | 6133267 | 625 | 99.36 |
| U^£¥$^ | - | 6536133 | 6538085 | 1953 | 87.11 |
| U^£¥$^ | + | 6720385 | 6721210 | 826 | 96.73 |
| U^£^ | + | 6958505 | 6960048 | 1543 | 95.46 |
| U^£¥^ | + | 7409968 | 7410136 | 169 | 95.83 |
| U^£¥^ | - | 8905908 | 8906162 | 255 | 98.43 |
| U^£^ | - | 9126245 | 9126804 | 559 | 95.40 |
| U^£¥^ | + | 9852927 | 9853034 | 108 | 89.81 |
| U^£^ | + | 10162660 | 10163444 | 784 | 96.30 |
| U^£¥^ | + | 10317347 | 10317513 | 167 | 82.04 |
| U^£¥^ | + | 10625041 | 10625415 | 375 | 94.31 |
| U^£¥^ | + | 10862415 | 10864252 | 1838 | 97.82 |
| U^£^ | - | 11024602 | 11025244 | 643 | 91.19 |
| U^£$^ | - | 11023792 | 11024075 | 284 | 94.72 |
| U^£^ | - | 12899234 | 12899861 | 627 | 93.95 |
| U^£¥$^ | - | 13548152 | 13549068 | 917 | 95.85 |
| U^¥^ | - | 13613535 | 13613737 | 203 | 94.58 |
| U^¥^ | - | 15302493 | 15302886 | 394 | 97.21 |

^§^the complete *helena* sequence

^*^ sequences with internal deletions and insertions

^$^ sequences with internal deletions

^#^ sequence with insertions

^£^sequences truncated in 5'

^¥^sequences truncated in 3'
