# Supplementary material for: Losing helena: The extinction of a drosophila line-like element
Source: BMC Genomics. 2008 Mar 31;9:149. doi: 10.1186/1471-2164-9-149 (PMC2330053; doi:10.1186/1471-2164-9-149)
Supplement: Additional File 2 — helena copies in the Drosophila melanogaster sequenced genome. The data provided is a list of the D. melanogaster copies. [file 1471-2164-9-149-S2.doc]

Supplementary Table 2: *helena* copies in the *Drosophila melanogaster* sequenced genome

| **chromosome** | **strand** | **start** | **stop** | **length (bp)** | **%identity with the complete *helena*** |
| --- | --- | --- | --- | --- | --- |
| 2L^$^ | + | 20553860 | 20554967 | 1108 | 94.00 |
| 2L | + | 20502277 | 20502573 | 297 | 76.00 |
| 2L^*^ | - | 21790993 | 21793986 | 2994 | 61.00 |
| 2R^*^ | - | 483468 | 488273 | 4805 | 83.00 |
| 2R^*^ | + | 1630552 | 1632697 | 2146 | 64.66 |
| 2R^*^ | - | 1841627 | 1843766 | 1907 | 68.81 |
| 3L^#^ | + | 16603717 | 16607074 | 3358 | 65.00 |
| 3L | + | 18688097 | 18688680 | 583 | 70.05 |
| 3L^$^ | - | 23487977 | 23490595 | 2619 | 93.00 |
| 3R | - | 484629 | 484719 | 91 | 79.00 |
| 3R | - | 401148 | 401432 | 285 | 92.00 |
| 3R^*^ | + | 3313732 | 3314009 | 278 | 95.00 |
| 3R^*^ | - | 3884273 | 3886085 | 1813 | 70.21 |
| 3R^*^ | - | 7777247 | 7779392 | 2146 | 64.65 |
| X | - | 18697277 | 18697732 | 458 | 84.00 |
| X^*^ | - | 22519248 | 22521013 | 1765 | 90.00 |
| X^$^ | - | 2255945 | 2256468 | 524 | 88.00 |
| U | + | 1548034 | 1548124 | 91 | 86.00 |
| U | + | 1572147 | 1572700 | 554 | 90.00 |
| U | - | 3163038 | 3163543 | 506 | 72.00 |
| U | - | 3164132 | 3164716 | 585 | 70.00 |
| U^$^ | - | 390489 | 392808 | 2320 | 87.00 |
| U^$^ | + | 4397920 | 4400343 | 2423 | 94.00 |
| U^*^ | + | 5254588 | 5256562 | 1975 | 67.34 |
| U | + | 5813553 | 5813690 | 138 | 92.00 |
| U | + | 5814272 | 5814990 | 719 | 95.00 |

^*^ sequences with internal deletions and insertions

^$^ sequences with internal deletions

^#^ sequence with insertions
