# Supplementary material for: Losing helena: The extinction of a drosophila line-like element
Source: BMC Genomics. 2008 Mar 31;9:149. doi: 10.1186/1471-2164-9-149 (PMC2330053; doi:10.1186/1471-2164-9-149)
Supplement: Additional File 4 — Alignment of helena ORF1. The data provided the alignement used to construct the tree on Figure 4 [file 1471-2164-9-149-S4.pdf]

```
Alignment: /home/lerat/MANIP/simulans/HELENA/ARTICLE/revision/alignment_ORF1.ali
Seaview [blocks=10 fontsize=10 A4] on Fri Dec 21 10:42:44 2007

                   1
chr3R_1506433      ATGGATACGA ACAACGAAAC TTCTAACTCT CAGCTTA--G AAATAAGCCG ATTGATTATG
Brazzaville_16     ---------- ---------- ---------- ---------- ---------- ----------
Eden_3             ---------- ---------- ---------- ---------- ---------- ----------
Cann-River_9       ---------- ---------- ---------- ---------- ---------- ----------
Amieu_2            ---------- ---------- ---------- ---------- ---------- ----------
Makindu_2          ---------- ---------- ---------- ---------- ---------- -------ATG
Makindu_6          ---------- ---------- ---------- ---------- AAATAAGCCG ATTGATTATG
Makindu_7          ATGGATACGA ACAACGAAAC TTCTAACTCT CAGCTTA--G AAATAAGCCG ATTGATTATG
Valence_6          ATGGATACGA ACAACGAAAC TTCTAACTCT CAGCTTA--G AAATAAGCCG ATTGATTATG
Makindu_8          ATGGATACGA ACAACGAAAC TTCTAACTCT CAGCTTA--G AAATAAGCCG ATTGATTATG
Makindu_22         ATGGATACGA ACAACGAAAC TTCTAACTCT CAGCTTA--G AAATAAGCCG ATTGATTATG
Canberra_5         -----TAC-A ACAACGAAAC TTCTAACTCT CAGCTTA--G AAATAAGCCG ATTGATTATG
Cann-River_2       ATGGATACGA ACAACGAAAC TTCTAACTCT CAGCTTA--G AAATAAGCCG ATTGATTATG
Cann-River_6       ATGGATACGA ACAACGAAAC TTCTAACTCT CAGCTTA--G AAATAAGCCG ATTGATTATG
Cann-River_7       ATGGATACGA ACAACGAAAC TTCTAACTCT CAGCTTA--G AAATAAGCCG ATTGATTATG
Cann_River_5       --GGATACGA ACAACGAAAC TTCTAACTCT CAGCTTA--G AAATAAGCCG ATTGATTATG
Cann-River_8       --GGATACGA ACAGCGAAAC TTCTAACTCT CAGCTTA--G AAATAAGCCG ATTGATTATG
Valence_5          ATGGATACGA ACAACGAAAC TTCTAACTCT CAGCTTA--G AAATAAGCCG ATTGATTATG
Valence_9          ---GATACGA ACAACGAAAC TTCTAACTCT CAGCTTA--G AAATAAGCCG ATTGATTATG
Eden_1             ATGGATACGA ACAACGAAAC TTCTAACTCT CAGCTTA--G AAATAAGCCG ATTGATTATG
Valence_13         ---gatACGA ACAACGAAAC TTCTAACTCT CAGCTTA--G AAATAAGCCG ATTGATTATG
Eden_7             ATGGATACGA ACAACGAAAC TTCTAACTCT CAGCTTA--G AAATAAGCCG ATTGATTATG
Valence_4          ATGGATACGA ACAACGAAAC TTCTAACTCT CAGCTTA--G AAATAAGCCG ATTGATTATG
Valence_2          ---GATACGA ACAACGAAAC TTCTAACTCT CAGCTTA--G AAATAAGCCG ATTGATTATG
Valence_1          --GGATACGA ACAACGAAAC TTCTAACTCT CAGCTTA--G AAATAAGCCG ATTGATTATG
Valence_7          ATGGATACGA ACAACGAAAC TTCTAACTCT CAGCTTA--G AAATAAGCCG ATTGATTATG
Makindu_cM1        ATGGATACGA ACAACGAAAC TTCTAACTCT CAGCTTA--G AAATAAGCCG ATTGATTATG
Makindu_cM6        ATGGATACGA ACAACGAAAC TTCTAACTCT CAGCTTA--G AAATAAGCCG ATTGATTATG
Makindu_1          ATGGATACGA ACAACGAAAC TTCTAACTCT CAGCTTA--G AAATAAGCCG ATTGATTATG
Amieu_5            ATGGATACGA ACAACGAAAC TTCTAACTCT CAGCTTA--G AAATAAGCCG ATTGATTATG
Eden_11            ATGGATACGA ACAACGAAAC TTCTAACTCT CAGCTTA--G AAATAAGCCG ATTGATTATG
Cann-River_10      ATGGATACGA ACAACGAAAC TTCTAACTCT CAGCTTA--G AAATAA---- ----------
Brazzaville_10     ATGGATACGA ACAACGAAAC TTCTAACTCT CAGCTTA--G AAATAA---- ----------
Cann-River_3       ATGGATACGA ACAACGAAAC TTCTAACTCT CAGCTTA--G AAATAAGCCG ATTGATTATG
Cann-River_13      ATGGATACGA ACAATGAAAC TTCTAACTCT CAGCTTA--G AAATAAGCCG ATTGATTATG
Eden_4             --GGATACGA ACAATGAAAC TTCTAACTCT CAGCTTA--G AAATAAGCCG ATTGATTATG
Makindu_10         ATGGATACGA ACAATGAAAC TTCTAACTCT CAGCTTA--G AAATAAGCCG ATTGATTACG
Makindu_11         ATGGATACGA ACAATGAAAC TTCTAACTCT CAGCTTA--G AAATAAGCCG ATTGATTACG
Brazzaville_11     ATGGATACGA ACAACGAAAC TTCTAACTCT CAGCTTA--G AAATAAGCCG ATTGATTATG
Brazzaville_12     ATGGATACGA ACAACGAAAC TTCTAACTCT CAGCTTA--G AAATAAGCCG ATTGATTATG
Brazzaville_8      ATGGATACGA ACAACGAAAC TTCTAACTCT CAGCTTA--G AAATAAGCCG ATTGATTATG
chrU_963982        ATGGATACGA ACAACGAAAC TTCTAACTCT CAGCTTA--G AAATAAGCCG ATTGATTATG
chr3L_18642496     ATGGATACGA ACAACGAAAC TTCTAACTCT CAGCTTA--G AAATAAGCCG ATTGATTATG
chrU_3975907       ATGGATACGA ACAACGAAAC TTCTAACTCT CAGCTTA--G AAA--AGCCG ATTGATTGTG
chrU_10625041      ---------- ---------- ---------- ---------- ---------- ----------
chrU_2316095       ---------- ---------- ---------- ---------- ---------- ----------
chrU_4537377       ---------- ---------- ---------- ---------- ---------- ----------
chrU_5384045       ---------- ---------- ---------- ---------- ---------- ----------
chrU_5680622       ATGGATACGA ACAACGAAAC TTCTAACTCT CAGCTTA--G AAATAAGCCG ATTGATTATG
chrU_5950518       ---------- ---------- ---------- ---------- ---------- ----------
chrU_6132643       ATGGATACGA ACAACGAAAC TTCTAACTCT CAGCTTA--G AAATAAGCCG ATTGATTATG
chrX_16602314      ---------- ---------- ---------- ---------- ---------- ----------
chrU_6536133       ---------- ---------- ---------- ---------- ---------- ----------
```

```
                61
chr3R_1506433   AAGAAATGCG GCAAGTAGCT GGTAAGTCTC CACTGGATAT CAGGATGGAA TTATTGAAT-
Brazzaville_16  ---------- ---------- ---------- ---------- ---------- ----------
Eden_3          ---------- ---------- ---------- ---------- ---------- ----------
Cann-River_9    ---------- ---------- ---------- ---------- ---------- ----------
Amieu_2         ---------- ---------- ---------- ---------- ---------- ----------
Makindu_2       AAGAAATGCG GCAAGTAGCT GGTAAGTCTC CACTGAATAT CAGGATGGAG TTATTGAAC-
Makindu_6       AAGAAATGCG GCAAGTAGCT GGTAAGTCTC CACTGAATAT CAGGATGGAG TTATTGAAC-
Makindu_7       AAGAAATGCG GCAAGTAGCT GGTAAGTCTC CACTGAATAT CAGGATGGAG TTATTGAAC-
Valence_6       AAGAAATGCG GCAAGTAGCT GGTAAGTCTC CACTGAATAT CAGGATGGAG TTATTGAAC-
Makindu_8       AAGAAATGCG GCAAGTAGCT GGTAAGTCTC CACTGAATAT CAGGATGGAG TTATTGAAC-
Makindu_22      AAGAAATGCG GCAAGTAGCT GGTAAGTCTC CACTGGATAT CAGGATGGAA TTATTGAAT-
Canberra_5      AAGAAATGCG GCAAGTAGCT GGTAAGTCTC CACTGAATAT CAGGATGGAG TTATTGAAC-
Cann-River_2    AAGAAATGCG GCAAGTAGCT GGTAAGTCTC CACTGAATAT CAGGATGGAG TTATTGAAC-
Cann-River_6    AAGAAATGCG GCAAGTAGCT GGTAAGTCTC CACTGAATAT CAGGATGGAG TTATTGAAC-
Cann-River_7    AAGAAATGCG GCAAGTAGCT GGTAAGTCTC CACTGAATAT CAGGATGGAG TTATTGAAC-
Cann_River_5    AAGAAATGCG GCAAGTAGCT GGTAAGTCTC CACTGAATAT CAGGATGGAG TTATTGAAC-
Cann-River_8    AAGAAATGCG GCAAGTAGCT GGTAAGTCTC CACTGGATAT CAGGATGGAA TTATTGAAT-
Valence_5       AAGAAATGCG GCAAGTAGCT GGTAAGTCTC CACTGAATAT CAGGATGGAG TTATTGAAC-
Valence_9       AAGAAATGCG GCAAGTAGCT GGTAAGTCTC CACTGAATAT CAGGATGGAG TTATTGAAC-
Eden_1          AAGAAATGCG GCAAGTAGCT GGTAAGTCTC CACTGAATAT CAGGATGGAG TTATTGAAC-
Valence_13      AAGAAATGCG GCAAGTAGCT GGTAAGTCTC CACTGAATAT CAGGATGGAG TTATTGAAC-
Eden_7          AAGAAATGCG GCAAGTAGCT GGTAAGTCTC CACTGAATAT CAGGATGGAG TTATTGAAC-
Valence_4       AAGAAATGCG GCAAGTAGCT GGTAAGTCTC CACTGAATAT CAGGATGGAG TTATTGAAC-
Valence_2       AAGAAATGCG GCAAGTAGCT GGTAAGTCTC CACTGAATAT CAGGATGGAG TTATTGAAC-
Valence_1       AAGAAATGCG GCAAGTAGCT GGTAAGTCTC CACTGAATAT CAGGATGGAG TTATTGAAC-
Valence_7       AAGAAATGCG GCAAGTAGCT GGTAAGTCTC CACTGAATAT CAGGATGGAG TTATTGAAC-
Makindu_cM1     AAGAAATGCG GCAAGTAGCT GGTAAGTCTC CACTGAATAT CAGGATGGAG TTATTGAAC-
Makindu_cM6     AAGAAATGCG GCAAGTAGCT GGTAAGTCTC CACTGAATAT CAGGATGGAG TTATTGAAC-
Makindu_1       AAGAAATGCG GCAAGTAGCT GGTAAGTCTC CACTGAATAT CAGGATGGAG TTATTGAAC-
Amieu_5         AAGAAATGCG GCAAGTAGCT GGTAAGTCTC CACTGGATAT CAGGATGGAA TTATTGAAT-
Eden_11         AAGAAATGCG GCAAGTAGCT GGTAAGTCTC CACAGGATAT CAGGATGGAG TAATGGAAT-
Cann-River_10   ---------- ---------- ---------- ---------- ---------- ----------
Brazzaville_10  ---------- ---------- ---------- ---------- ---------- ----------
Cann-River_3    AAGAAATGCG GCAAGTAGCT GGTAAGTCCC CACTGGATAT CAGGATGGAA TTATTGAAT-
Cann-River_13   AAGAAATGCG GCAAGTAGCT GGTAAGTCTC CACAGGATAT CAGGATGGAG TAATGGAAT-
Eden_4          AAGAAATGCG GCAAGTAGCT GGTAAGTCTC CACAGGATAT CAGGATGGAG TAATGGAAT-
Makindu_10      AAGAAATGCG GCAAGTAGCT GGTAAGTCTC CACAGGATAT CAGGATGGAG TAATGGAAT-
Makindu_11      AAGAAATGCG GCAAGTAGCT GGTAAGTCTC CACAGGATAT CAGGATGGAG TAATGGAAT-
Brazzaville_11  AAGAAATGCG GCAAGTAGCT GGTAAGTCTC CACTGAATAT CAGGATGGAG TTATTGAAC-
Brazzaville_12  AAGAAATGCG GCAAGTAGCT GGTAAGTCTC CACTGAATAT CAGGATGGAG TTATTGAAC-
Brazzaville_8   AAGAAATGCG GCAAGTAGCT GGTAAGTCTC CACTGAATAT CAGGATGGAG TTATTGAAC-
chrU_963982     AAGAAATGCG GCAAGTAGCT GGTAAGTCTC CACTGGATAT CAGGATGGAG TTATTGAAT-
chr3L_18642496  AAGAAATGCG GCAAGTAGCT GGTAAGTCTC CACTGGATAT CAGGATGGAA TTATTGAAT-
chrU_3975907    AAGAAATGCG GCAAGTAGCT GGTAAGTCTC CGCTGGATAT CAGGATGGAG TTATTGAAT-
chrU_10625041   ---------- ---------- ---------- ---------- ---------- ----------
chrU_2316095    ---------- ---------- ---------- ---------- ---------- ----------
chrU_4537377    ---------- ---------- ---------- ---------- ---------- ----------
chrU_5384045    ---------- ---------- ---------- ---------- ---------- ----------
chrU_5680622    AAGAAATGCG GCAAGTAGCT GGTAAGTCTC CACTGGATAT CAGGATGGAG TTATTGAAT-
chrU_5950518    ---------- ---------- ---------- ---------- ---------- ----------
chrU_6132643    AAGAAATGCG GCAAGTAGCT GGTAAGTCTC CACTGGATAT CAGGATGGAA TTATTGAAT-
chrX_16602314   ---------- ---------- ---------- ---------- ---------- ----------
chrU_6536133    ---------- ---------- -GTAAATCTC CACTGGAGAT CAGGATGGAG TTATTGAATA
```

```
                121
chr3R_1506433    --CAACGCTC TTCATGCACT GAATCTGTTA ACCTAAGCAC AATAACTACG ACGATAACAT
Brazzaville_16   ---------- ---------- ---------- ---------- ---------- ----------
Eden_3           ---------- ---------- ---------- ---------- ---------- ----------
Cann-River_9     ---------- ---------- ---------- ---------- ---------- ----------
Amieu_2          ---------- ---------- ---------- ---------- ---------- ----------
Makindu_2        --CAACGCTT TTCATGCACT GAATCTGTTA ACCTAAGCAC AATAACTACG ACGATAACAT
Makindu_6        --CAACGCTT TTCATGCACT GAATCTGTTA ACCTAAGCAC AATAACTACG ACGATAACAT
Makindu_7        --CAACGCTT TTCATGCACT GAATCTGTTA ACCTAAGCAC AATAACTACG ACGATAACAT
Valence_6        --CAACGCTT TTCATGCACT GAATCTGTTA ACCTAAGCAC AATAACTACG ACGATAACAT
Makindu_8        --CAACGCTT TTCATGCACT GAATCTGTTA ACCTAAGCAC AATAACTACG ACGATAACAT
Makindu_22       --CAACGCTC TTCATGCACT GAATCTGTTA ACCTAAGCAC AATAACTACG ACGATAACAT
Canberra_5       --CAACGCTT TTCATGCACT GAATCTGTTA ACCTAAGCAC AATAACTACG ACGATAACAT
Cann-River_2     --CAACGCTT TTCATGCACT GAATCTGTTA ACCTAAGCAC AATAACTACG ACGATAACAT
Cann-River_6     --CAACGCTT TTCATGCACT GAATCTGTTA ACCTAAGCAC AATAACTACG ACGATAACAT
Cann-River_7     --CAACGCTT TTCATGCACT GAATCTGTTA ACCTAAGCAC AATAACTACG ACGATAACAT
Cann_River_5     --CAACGCTT TTCATGCACT GAATCTGTTA ACCTAAGCAC AATAACTACG ACGATAACAT
Cann-River_8     --CAACGCTC TTCATGCACT GAATCTGTTA ACCTAAGCAC AATAACTACG ACGATAACAT
Valence_5        --CAACGCTT TTCATGCACT GAATCTGTTA ACCTAAGCAC AATAACTACG ACGATAACAT
Valence_9        --CAACGCTT TTCATGCACT GAATCTGTTA ACCTAAGCAC AATAACTACG ACGATAACAT
Eden_1           --CAACGCTT TTCATGCACT GAATCTGTTA ACCTAAGCGC AATAACTGCG ACGATAACAT
Valence_13       --CAACGCTT TTCATGCACT GAATCTGTTA ACCTAAGCAC AATAACTACG ACGATAACAT
Eden_7           --CAACGCTT TTCATGCACT GAATCTGTTA ACCTAAGCGC AATAACTGCG ACGATAACAT
Valence_4        --CAACGCTT TTCATGCACT GAATCTGTTA ACCTAAGCAC AATAACTACG ACGATAACAT
Valence_2        --CAACGCTT TTCATGCACT GAATCTGTTA ACCTAAGCAC AATAACTACG ACGATAACAT
Valence_1        --CAACGCTT TTCATGCACT GAATCTGTTA ACCTAAGCAC AATAACTACG ACGATAACAT
Valence_7        --CAACGCTT TTCATGCACT GAATCTGTTA ACCTAAGCAC AATAACTACG ACGATAACAT
Makindu_cM1      --CAACGCTT TTCATGCACT GAATCTGTTA ACCTAAGCAC AATAACTACG ACGATAACAT
Makindu_cM6      --CAACGCTT TTCATGCACT GAATCTGTTA ACCTAAGCAC AATAACTACG ACGATAACAT
Makindu_1        --CAACGCTT TTCATGCACT GAATCTGTTA ACCTAAGCAC AATAACTACG ACGATAACAT
Amieu_5          --CAACGCTC TTCATGCACT GAATCTGTTA AC--AAGCAC AATAACTACG ACGATAACAT
Eden_11          --CAACGCTT TTCATGCACT GAATCTGTTA ACCTAAGCAC AATA-CTAC- ----------
Cann-River_10    ---------- ---------- ---------- ---------- --------CG ACGATAACAT
Brazzaville_10   ---------- ---------- ---------- ---------- --------CG ACGATAACAT
Cann-River_3     --CAACGCTC TTCATGCACT GAATCTGTTA ACCTAAGCAC AATAACTACG ACGATAACAT
Cann-River_13    --CAACGCTT TTCATGCACT GAATCTGTTA ACCTAAGCAC AATA-CTAC- ----------
Eden_4           --CAACGCTT TTCATGCACT GAATCTGTTA ACCTAAGCAC AATA-CTAC- ----------
Makindu_10       --CAACGCTT TTCATGCACT GAATCTGTTA ACCTAAGCAC AATA-CTAC- ----------
Makindu_11       --CAACGCTT TTCATGCACT GAATCTGTTA ACCTAAGCAC AATA-CTAC- ----------
Brazzaville_11   --CAACGCTT TTCATGCACT GAATCTGTTA ACCTAAGCAC AATAACTACG ACGATAACAT
Brazzaville_12   --CAACGCTT TTCATGCACT GAATCTGTTA ACCTAAGCAC AATAACTACG ACGATAACAT
Brazzaville_8    --CAACGCTT TTCATGCACT GAACCTGTTA ACCTAAGCAC AATAACTACg ACGATAACAT
chrU_963982      --CAACGCTT TTCATGCACT GAATCTGTTA ACCTAAGCAC AATATCTACG ACGATAACAT
chr3L_18642496   --CAACGCTC TTCATGCACT GAATCTGTTA ACCTAAGCAC AATAACTACG ACGATAACAT
chrU_3975907     --CAACGCTT TTCATGCACT GAATCTGTTA ACCTAAGCAC AATAACTACG ACGATAACAT
chrU_10625041    ---------- ---------- ---------- ---------- ---------- ----------
chrU_2316095     ---------- ---------- ---------- ---------- ---------- ----------
chrU_4537377     ---------- ---------- ---------- ---------- ---------- ----------
chrU_5384045     ---------- ---------- ---------- ---------- ---------- ----------
chrU_5680622     --CAACGCTT TTCATGCACT GAATCTGTTA ACCTAAGCAC AATAACTACG ACGATAACAT
chrU_5950518     ---------- ---------- ---------- ---------- ---------- ----------
chrU_6132643     --CAACGCTC TTCATGCACT GAATCTGTTA ACCTAAGCAC AATAACTACG ACGATAACAT
chrX_16602314    ---------- ---------- ---------- ---------- ---------- ----------
chrU_6536133     ATCAACGCTT TTCATGCACT GAATCTGGTA ACCTAAGCAC AATAACTACG ACGGCAACAT
```

```
                    181
chr3R_1506433     TTACAAGTGC AACTTGCAAT ACTACATCTA CAACAACTGC AGCAATCAGT AGGCCGAGCT
Brazzaville_16    ---------- ---------- ---------- ---------- ---------- ----------
Eden_3            ---------- ---------- ---------- ---------- ---------- ----------
Cann-River_9      ---------- ---------- ---------- ---------- ---------- ----------
Amieu_2           ---------- ---------- ---------- ---------- ---------- ----------
Makindu_2         TTACAAGTGC TACTTGCAAT ACTACATCTA CAACAACTGC AGCAATCAGT AGGCCGAGCT
Makindu_6         TTACAAGTGC TACTTGCAAT ACTACATCTA CAACAACTGC AGCAATCAGT AGGCCGAGCT
Makindu_7         TTACAAGTGC TACTTGCAAT ACTACATCTA CAACAACTGC AGCAATCAGT AGGCCGAGCT
Valence_6         TTACAAGTGC TACTTGCAAT ACTACATCTA CAACAACTGC AGCAATCAGT AGGCCGAGCT
Makindu_8         TTACAAGTGC TACTTGCAAT ACTACATCTA CAACAACTGC AGCAATCAGT AGGCCGAGCT
Makindu_22        TTACAAGTGC AACTTGCAAT ACTACATCTA CAACAACTGC AGCAATCAGT AGGCCGAGCT
Canberra_5        TTACAAGTGC TACTTGCAAT ACTACATCTA CAACAACTGC AGCAATCAGT AGGCCGAGCT
Cann-River_2      TTACAAGTGC TACTTGCAAT ACTACATCTA CAACAACTGC AGCAATCAGT AGGCCGAGCT
Cann-River_6      TTACAAGTGC TACTTGCAAT ACTACATCTA CAACAACTGC AGCAATCAGT AGGCCGAGCT
Cann-River_7      TTACAAGTGC TACTTGCAAT ACTACATCTA CAACAACTGC AGCAATCAGT AGGCCGAGCT
Cann_River_5      TTACAAGTGC TACTTGCAAT ACTACATCTA CAACAACTGC AGCAATCAGT AGGCCGAGCT
Cann-River_8      TTACAAGTGC AACTTGCAAT ACTACATCTA CAACAACTGC AGCAATCAGT AGGCCGAGCT
Valence_5         TTACAAGTGC TACTTGCAAT ACTACATCTA CAACAACTGC AGCAATCAGT AGGCCGAGCT
Valence_9         TTACAAGTGC TACTTGCAAT ACTACATCTA CAACAACTGC AGCAATCAGT AGGCCGAGCT
Eden_1            TTACAAGTGC TACTTGCAAT ACTACATCTA CAACAACTGC AGCAATCAGT AGGCCGAGCT
Valence_13        TTACAAGTGC TACTTGCAAT ACTACATCTA CAACAACTGC AGCAATCAGT AGGCCGAGCT
Eden_7            TTACAAGTGC TACTTGCAAT ACTACATCTA CAACAACTGC AGCAATCAGT AGGCCGAGCT
Valence_4         TTACAAGTGC TACTTGCAAT ACTACATCTA CAACAACTGC AGCAATCAGT AGGCCGAGCT
Valence_2         TTACAAGTGC TACTTGCAAT ACTACATCTA CAACAACTGC AGCAATCAGT AGGCCGAGCT
Valence_1         TTACAAGTGC TACTTGCAAT ACTACATCTA CAACAACTGC AGCAATCAGT AGGCCGAGCT
Valence_7         TTACAAGTGC TACTTGCAAT ACTACATCTA CAACAACTGC AGCAATCAGT AGGCCGAGCT
Makindu_cM1       TTACAAGTGC TACTTGCAAT ACTACATCTA CAACAACTGC AGCAATCAGT AGGCCGAGCT
Makindu_cM6       TTACAAGTGC TACTTGCAAT ACTACATCTA CAACAACTGC AGCAATCAGT AGGCCGAGCT
Makindu_1         TTACAAGTGC TACTTGCAAT ACTACATCTA CAACAACTGC AGCAATCAGT AGGCCGAGCT
Amieu_5           TTACAAGTGC AACTTGCAAT ACTACATCTA CAACAACTGC AGCAATCAGT AGGCCGAGCT
Eden_11           ---------- --------AT ACTACATCTA CAACAACTAC AGCAATCAGT AGGCCGAGCT
Cann-River_10     TTACAAGTGC TACTTGCAAT ACTACATCTA CTACAACTGC AGCAATCAGT AGGCCGAGCT
Brazzaville_10    TTACAAGTGC TACTTGCAAT ACTACATCTA CTACAACTGC AGCAATCAGT AGGCCGAGCT
Cann-River_3      TTACAAGTGC AACTTGCAAT ACTACATCTA CAACAACTGC AGCAATCAGT AGGCCGAGCT
Cann-River_13     ---------- --------AT ACTACATCTA CAACAACTAC AGCAATCAGT AGGCCGAGCT
Eden_4            ---------- --------AT ACTACATCTA CAACAACTAC AGCAATCAGT AGGCCGAGCT
Makindu_10        ---------- --------AT ACTACATCTA CAACAACTAC AGCAATCAGT AGGCCGAGCT
Makindu_11        ---------- --------AT ACTACATCTA CAACAACTAC AGCAATCAGT AGGCCGAGCT
Brazzaville_11    TTACAAGTGC TACTTGCAAT ACTACATCTA CAACAACTGC AGCAATCAGT AGGCCGAGCT
Brazzaville_12    TTACAAGTGC TACTTGCAAT ACTACATCTA CAACAACTGC AGCAATCAGT AGGCCGAGCT
Brazzaville_8     TTACAAGTGC TACTTGCAAT ACTACATCTA CAACAACtGC agcAATCAGT AGGCCGAGCT
chrU_963982       TTACAAGTGC TACTTGCAAT ACTACATCTA CAACACCTGC AGCAATCAGT AGGCCGAGCT
chr3L_18642496    TTACAAGTGC AACTTGCAAT ACTACATCTA CAACAACTGC AGCAATCAGT AGGCCGAGCT
chrU_3975907      TTACAAGTGC TACTTGCAAA ACTACATCTA CTACAACTGC AGCAATCAGT AGGCCGAGCT
chrU_10625041     ---------- ---------- ---------- ---------- ---------- ----------
chrU_2316095      ---------- ---------- ---------- ---------- ---------- ----------
chrU_4537377      ---------- ---------- ---------- ---------- ---------- ----------
chrU_5384045      ---------- ---------- ---------- ---------- ---------- ----------
chrU_5680622      TTACAAGTTC TACTTGCAAT ACTACATCTA CAACAACTGC AGCAATCAGT AGGCCGAGCT
chrU_5950518      ---------- ---------- ---------- ---------- ---------- ----------
chrU_6132643      TTACAAGTGC AACTTGCAAT ACTACATCTA CAACAACTGC AGCAATCAGT AGGCCGAGCT
chrX_16602314     ---------- ---------- ---------- ---------- ---------- ----------
chrU_6536133      TTACATGTGT TACTTGCAAT ACTACATCTA CTACA----- ---------- ----------
```

```
                241
chr3R_1506433   CCAGAGAAAA AGCTGCTACT AGATTGTCTG ATGCGCAAAA ACTTCTGACG AAAGAGAGGA
Brazzaville_16  ---------- ---------- ---------- ---------- ---------- ----------
Eden_3          ---------- ---------- ---------- ---------- ---------- ----------
Cann-River_9    ---------- ---------- ---------- ---------- ---------- ----------
Amieu_2         ---------- ---------- ---------- ---------- -CTTCTGACG AAAGAGAGGA
Makindu_2       CCAGAGAAAA AGCTGCTACT AGATTGTCTG ATGCGCAAAA ACTTCTGACG AAAGAGAGGA
Makindu_6       CCAGAGAAAA AGCTGCTACT AGATTGTCTG ATGCGCAAAA ACTTCTGACG AAAGAGAGGA
Makindu_7       CCAGAGAAAA AGCTGCTACT AGATTGTCTG ATGCGCAAAA ACTTCTGACG AAAGAGAGGA
Valence_6       CCAGAGAAAA AGCTGCTACT AGATTGTCTG ATGCGCAAAA ACTTCTGACG AAAGAGAGGA
Makindu_8       CCAGAGAAAA AGCTGCTACT AGATTGTCTG ATGCGCAAAA ACTTCTGACG AAAGAGAGGA
Makindu_22      CCAGAGAAAA AGCTGCTACT AGATTGTCTG ATGCGCAAAA ACTTCTGACG AAAGAGAGGA
Canberra_5      CCAGAGAAAA AGCTGCTACT AGATTGTCTG ATGCGCAAAA ACTTCTGACG AAAGAGAGGA
Cann-River_2    CCAGAGAAAA AGCTGCTACT AGATTGTCTG ATGCGCAAAA ACTTCTGACG AAAGAGAGGA
Cann-River_6    CCAGAGAAAA AGCTGCTACT AGATTGTCTG ATGCGCAAAA ACTTCTGACG AAAGAGAGGA
Cann-River_7    CCAGAGAAAA AGCTGCTACT AGATTGTCTG ATGCGCAAAA ACTTCTGACG AAAGAGAGGA
Cann_River_5    CCAGAGAAAA AGCTGCTACT AGATTGTCTG ATGTGCAAAA ACTTCTGACG AAAGAGAGGA
Cann-River_8    CCAGAGAAAA AGCTGCTACT AGATTGTCTG ATGCGCAAAA ACTTCTGACG AAAGAGAGGA
Valence_5       CCAGAGAAAA AGCTGCTACT AGATTGTCTG ATGCGCAAAA ACTTCTGACG AAAGAGAGGA
Valence_9       CCAGAGAAAA AGCTGCTACT AGATTGTCTG ATGCGCAAAA ACTTCTGACG AAAGAGAGGA
Eden_1          CCAGAGAAAA AGCTGCTACT AGATTGTCTG ATGCGCAAAA ACTTCTGACG AAAGAGAGGA
Valence_13      CCAGAGAAAA AGCTGCTACT AGATTGTCTG ATGCGCAAAA ACTTCTGACG AAAGAGAGGA
Eden_7          CCAGAGAAAA AGCTGCTACT AGATTGTCTG ATGCGCAAAA ACTTCTGACG AAAGAGAGGA
Valence_4       CCAGAGAAAA AGCTGCTACT AGATTGTCTG ATGCGCAAAA ACTTCTGACG AAAGAGAGGA
Valence_2       CCAGAGAAAA AGCTGCTACT AGATTGTCTG ATGCGCAAAA ACTTCTGACG AAAGAGAGGA
Valence_1       CCAGAGAAAA AGCTGCTACT AGATTGTCTG ATGCGCAAAA ACTTCTGACG AAAGAGAGGA
Valence_7       CCAGAGAAAA AGCTGCTACT AGATTGTCTG ATGCGCAAAA ACTTCTGACG AAAGAGAGGA
Makindu_cM1     CCAGAGAAAA AGCTGCTACT AGATTGTCTG ATGCGCAAAA ACTTCTGACG AAAGAGAGGA
Makindu_cM6     CCAGAGAAAA AGCTGCTACT AGATTGTCTG ATGCGCAAAA ACTTCTGACG AAAGAGAGGA
Makindu_1       CCAGAGAAAA AGCTGCTACT AGATTGTCTG ATGCGCAAAA ACTTCTGACG AAAGAGAGGA
Amieu_5         CCAGAGAAAA AGCTGCTACT AGATTGTCTG ATGCGCAAAA ACTTCTGACG AAAGAGAGGA
Eden_11         CCAGAGAAAA AGCTTCTACT AGATTGTCTG ATGCGCAAAA ACTTCTGACG AAAGAGAGGA
Cann-River_10   CCAGAGAAAA AGCAGCTACT AGATAGTCTG ATGCGTAAAA ACTTCTGACG AAAGAGAGGA
Brazzaville_10  CCAGAGAAAA AGCAGCTACT AGATAGTCTG ATGCGCAAAA ACTTCTGACG AAAGAGAGGA
Cann-River_3    CCAGAGAGAA AGCTGCTACT AGATTGTCTG ATGCGCAAAA ACTTCTGACG AAAGAGAGGA
Cann-River_13   CCAGAGAAAA AGCTTCTACT AGATTGTCTG ATGCGCAAAA ACTTCTGACG AAAGAGAGGA
Eden_4          CCAGAGAAAA AGCTTCTACT AGATTGTCTG ATGCGCAAAA ACTTCTGACG AAAGAGAGGG
Makindu_10      CCAGAGAAAA AGCTTCTACT AGATTGTCTG ATGCGCAAAA ACTTCTGACG AAAGAGAGGA
Makindu_11      CCAGAGAAAA AGCTTCTACT AGATTGTCTG ATGCGCAAAA ACTTCTGACG AAAGAGAGGA
Brazzaville_11  CCAGAGAAAA AGCTGCTACT AGATTGTCTG ATGCGCAAAG ACTTCTGACG AAAGAGAGGA
Brazzaville_12  CCAGAGAAAA AGCTGCTACT AGATTGTCTG ATGCGCAAAA ACTTCTGACG AAAGAGAGGA
Brazzaville_8   CCAGAGAAAA AGCCGCTACT AGATTGTCTG ATGCGCAAAA ACTTCTGACG AAAGAGAGGA
chrU_963982     CCAGAGAAAA AGCTGCTACT AGATTGTCTA ATGCGCAAAA ACTTCTGACG AAAGAGAGGA
chr3L_18642496  CCAGAGAAAA AGCTGCTACT AGATTGTCTG ATGCGCAAAA ACTTCTGACG AAAGAGAGGA
chrU_3975907    CCAGAGAAAA AGCTGCTACT AGATAGTCTG ATGCGCAAAA ACTTCTGACG AAAGAGAGGA
chrU_10625041   ---------- ---------- ---------- ---------- ---------- ----------
chrU_2316095    ---------- ---------- ---------- ---------- ---------- ----------
chrU_4537377    ---------- ---------- ---------- ---------- ---------- ----------
chrU_5384045    ---------- ---------- ---------- ---------- ---------- ----------
chrU_5680622    CTAGAGAAAA AGCTGCTACT AGATTGTCTG ATGCGCAAAA ACTTCTGACG AAAGAGAGGA
chrU_5950518    ---------- ---------- ---------- ---------- ---------- ----------
chrU_6132643    CCAGAGAAAA AGCTGCTACT AGATTGTCTG ATGCGCAAAA ACTTCTGACG AAAGAGAGGA
chrX_16602314   ---------- ---------- ---------- ---------- ---------- ----------
chrU_6536133    ---------- -----GCAAT CAGTAGTCTG ATGCGCAAAA CCTTTTGACG AAAGAGAGGA
```

```
                   301
chr3R_1506433      AGAGGAAGAC CAATTTAACA CCCAACC-AT AGCTCTAAAC GAGCTGTCAG AGACGCGAAG
Brazzaville_16     ---------- ---------- ---------- ---------- ---------- ----------
Eden_3             ---------- ---------- ---------- ---------- ---------- ----------
Cann-River_9       ---------- ---------- ---------- ------AAAC AAGCTGTCAG AGACGCGAAG
Amieu_2            AGAGGAAGAC CAATTTAACA CCCAACC-AT AGCTCTAAAC GAGCTGTCAG AGACGCGAAG
Makindu_2          AAAGGAAGAC CAATTTAACA CCCAACA-AT AGTTTTAAAC GAGCTGTCAG AGACGCGAAG
Makindu_6          AAAGGAAGAC CAATTTAACA CCCAACA-AT AGTTTTAAAC GAGCTGTCAG AGACGCGAAG
Makindu_7          AAAGGAAGAC CAATTTAACA CCCAACA-AT AGTTTTAAAC GAGCTGTCAG AGACGCGAAG
Valence_6          AAAGGAAGAC CAATTTAACA CCCAACA-AT AGTTTTAAAC GAGCTGTCAG AGACGCGAAG
Makindu_8          AAAGGAAGAC CAATTTAACA CCCAACA-AT AGTTTTAAAC GAGCTGTCAG AGACGCGAAG
Makindu_22         AAAGGAAGAC CAATTTAACA CCCAACA-AT AGTTTTAAAC GAGCTGTCAG AGACGCGAAG
Canberra_5         AAAGGAAGAC CAATTTAACA CCCAACA-AT AGTTTTAAAC GAGCTGTCAG AGACGCGAAG
Cann-River_2       AAAGGAAGAC CAATTTAACA CCCAACA-AT AGTTTTAAAC GAGCTGTCAG AGACGCGAAG
Cann-River_6       AAAGGAAGAC CAATTTAACA CCCAACA-AT AGTTTTAAAC GAGCTGTCAG AGACGCGAAG
Cann-River_7       AAAGGAAGAC CAATTTAACA CCCAACA-AT AGTTTTAAAC GAGCTGTCAG AGACGCGAAG
Cann_River_5       AAAGGAAGAC CAATTTAACA CCCAACA-AT AGTTTTAAAC GAGCTGTCAG AGACGCGAAG
Cann-River_8       AGAGGAAGAC CAATTTAACA CCCAACC-AT AGCTCTAAAC GAGCTGTCAG AGACGCGAAG
Valence_5          AAAGGAAGAC CAATTTAACA CCCAACA-AT AGTTTTAAAC GAGCTGTCAG AGACGCGAAG
Valence_9          AAAGGAAGAC CAATTTAACA CCCAACA-AT AGTTTTAAAC GAGCTGTCAG AGACGCGAAG
Eden_1             AAAGGAAGAC CAATTTAACA CCCAACA-AT AGTTTTAAAC GAGCTGTCAG AGACGCGAAG
Valence_13         AAAGGAAGAC CAATTTAACA CCCAACA-AT AGTTTTAAAC GAGCTGTCAG AGACGCGAAG
Eden_7             AAAGGAAGAC CAATTTAACA CCCAACA-AT AGTTTTAAAC GAGCTGTCAG AGACGCGAAG
Valence_4          AAAGGAAGAC CAATTTAACA CCCAACA-AT AGTTTTAAAC GAGCTGTCAG AGACGCGAAG
Valence_2          AAAGGAAGAC CAATTTAACA CCCAACA-AT AGTTTTAAAC GAGCTGTCAG AGACGCGAAG
Valence_1          AAAGGAAGAC CAATTTAACA CCCAACA-AT AGTTTTAAAC GAGCTGTCAG AGACGCGAAG
Valence_7          AAAGGAAGAC CAATTTAACA CCCAACA-AT AGTTTTAAAC GAGCTGTCAG AGACGCGAAG
Makindu_cM1        AAAGGAAGAC CAATTTAACA CCCAACA-AT AGTTTTAAAC GAGCTGTCAG AGACGCGAAG
Makindu_cM6        AAAGGAAGAC CAATTTAACA CCCAACA-AT AGTTTTAAAC GAGCTGTCAG AGACGCGAAG
Makindu_1          AAAGGAAGAC CAATTTAACA CCCAGCA-AT AGTTTTAAAC GAGCTGTCAG AGACGCGAAG
Amieu_5            AGAGGAAGAC CAATTTAACA CCCAACC-AT AGCTCTAAAC GAGCTGTCAG AGACGCGAAG
Eden_11            AGAGGAAGAC CAATTTAAAA CCCCACCCAT AGCTCTAAAC GAGCTGTCAG AGACGCGAAG
Cann-River_10      AGAGGAAGAC CAATTTAACA CCCAACC-AT AGCTCTAAAC GAGCTGTCAG AGACGCGAAG
Brazzaville_10     AGAGGAAGAC CAATTTAACA CCCAACC-AT AGCTCTAAAC GAGCTGTCAG AGACGCGAAG
Cann-River_3       AGAGGAAGAC CAATTTAACA CCCAACC-AT AGCTCTAAAC GAGCTGTCAG AGACGCGAAG
Cann-River_13      AGAGGAAGAC CAATTTAAAA CCCCACCCAT AGCTCTAAAC GAGCTGTCAG AGACGCGAAG
Eden_4             AGAGGAAGAC CAATTTAAAA CCCCACCCAT AGCTCTAAAC GAGCTGTCAG AGACGCGAAG
Makindu_10         AGAGGAAGAC CAATTTAAAA CCCCACCCAT AGCTCTAAAC GAGCTGTCAG AGACGCGAAG
Makindu_11         AGAGGAAGAC CAATTTAAAA CCCCACCCAT AGCTCTAAAC GAGCTGTCAG AGACGCGAAG
Brazzaville_11     AAAGGAAGAC CAATTTAACA CCCAACA-AT AGTTTTAAAC GAGCTGTCAG AGACGCGAAG
Brazzaville_12     AAAGGAAGAC CAATTTAACA CCCAACA-AT GGTTTTAAAC GAGCTGTCAG AGACGCGAAG
Brazzaville_8      AAAGGAAGAC CAATTTAACA CCCGACA-AT AGTTTTAAAC GAGCTGTCAG AGACGCGAAG
chrU_963982        AGAGGAAGAC CAATTTAACA CCCAACC-AT AGCTCTATAC GAGCTGTCAG AGGCGCGAAG
chr3L_18642496     AGAGGAAGAC CAATTTAACA CCCAACC-AT AGCTCTAAAC GAGCTGTCAG AGACGCGAAG
chrU_3975907       AGA------C CAATTTAATA CCCAACC-AT AGCTCTAAAC GAGCTGTCAG AGACGCGAAG
chrU_10625041      ---------- ---------- ---------- ---------- ---------- ----------
chrU_2316095       ---------- ---------- ---------- ---------- ---------- ----------
chrU_4537377       ---------- ---------- ---------- ---------- ---------- ----------
chrU_5384045       ---------- ---------- ---------- ---------- ---------- ----------
chrU_5680622       AGAGGAAGAC CAA------- -CCAACC-AT AGCTCTAAAC GAGTTGTCAG AGACGCGAAG
chrU_5950518       ---------- ---------- ---------- ---------- ---------- ----------
chrU_6132643       AGAGGAAGAC CAATTTAACA CCCAACC-AT AGCTCTAAAC GAGCTGTCAG AGACGCGAAG
chrX_16602314      ---------- ---------- ---------- ---------- ---------- ----------
chrU_6536133       AGCGGGAGAC AAATTTATAA CCCAACC-TT AGCGCTAAAA GAGCTGTCAG AGACGCGAAG
```

```
                 361
chr3R_1506433    CTGGAGCCAT ATCCAGCCAT AAATAGTAAT TCAAACTCAA ACAATAGGTT TGCCATGCTA
Brazzaville_16   ---------- ---------- ---------- ---------- ---AATCGTT TGGG------
Eden_3           ---------- ---------- ---------- ---------- ---------- ----------
Cann-River_9     CTGGAGCCAT ATCCAGCCAC AAATAGTAAT TCAAACTCAA ACAATAGGTT TGCCATGCTA
Amieu_2          CTGGAGCCAT GTCCAGCCAC AAATAGTAAT TCAAACTCAA ACAATAGGTT TGCCATGCCA
Makindu_2        CTGGAGCCAT ATCCAGCCAC AAATAGTAAT TCAAACTCAA ACAATAGGTT TGCCATGCTA
Makindu_6        CTGGAGCCAT ATCCAGCCAC AAATAGTAAT TCAAACTCAA ACAATAGGTT TGCCATGCTA
Makindu_7        CTGGAGCCAT ATCCAGCCAC AAATAGTAAT TCAAACTCAA ACAATAGGTT TGCCATGCTA
Valence_6        CTGGAGCCAT ATCCAGCCAC AAATAGTAAT TCAAACTCAA ACAATAGGTT TGCCATGCTG
Makindu_8        CTGGAGCCAT ATCCAGCCAC AAATAGTAAT TCAAACTCAA ACAATAGGTT TGCCATGCTA
Makindu_22       CTGGAGCCAT ATCCAGCCAC AAATAGTAAT TCAAACTCAA ACAATAGGTT TGCCATGCTA
Canberra_5       CTGGAGCCAT ATCCAGCCAC AAATAGTAAT TCAAACTCAA ACAATAGGTT TGCCATGCTA
Cann-River_2     CTGGAGCCAT ATCCAGCCAC AAATAGTAAT TCAAACTCAA ACAATAGGTT TGCCATGCTA
Cann-River_6     CTGGAGCCAT ATCCAGCCAC AAATAGTAAT TCAAACTCAA ACAATAGGTT TGCCATGCTA
Cann-River_7     CTGGAGCCAT ATCCAGCCAC AAATAGTAAT TCAAACTCAA ACAATAGGTT TGCCATGCTA
Cann_River_5     CTGGAGCCAT ATCCAGCCAC AAATAGTAAT TCAAACTCAA ACAATAGGTT TGCCATGCTA
Cann-River_8     CTGGAGCCAT ATCCAGCCAT AAATAGTAAT TCAAACTCAA ACAATAGGTT TGCCATGCTA
Valence_5        CTGGAGCCAT ATCCAGCCAC AAATAGTAAT TCAAACTCAA ACAATAGGTT TGCCATGCTG
Valence_9        CTGGAGCCAT ATCCAGCCAC AAATAGTAAT TCAAACTCAA ACAATAGGTT TGCCATGCTG
Eden_1           CTGGAGCCAT ATCCAGCCAC AAATAGTAAT TCAAACTCAA ACAATAGGTT TGCCATGCTA
Valence_13       CTGGAGCCAT ATCCAGCCAC AAATAGTAAT TCAAACTCAA ACAATAGGTT TGCCATGCTG
Eden_7           CTGGAGCCAT ATCCAGCCAC AAATAGTAAT TCAAACTCAA ACAATAGGTT TGCCATGCTA
Valence_4        CTGGAGCCAT ATCCAGCCAC AAATAGTAAT TCAAACTCAA ACAATAGGTT TGCCATGCTG
Valence_2        CTGGAGCCAT ATCCAGCCAC AAATAGTAAT TCAAACTCAA ACAATAGGTT TGCCATGCTG
Valence_1        CTGGAGCCAT ATCCAGCCAC AAATAGTAAT TCAAACTCAA ACAATAGGTT TGCCATGCTG
Valence_7        CTGGAGCCAT ATCCAGCCAC AAATAGTAAT TCAAACTCAA ACAATAGGTT TGCCATGCTG
Makindu_cM1      CTGGAGCCAT ATCCAGCCAC AAATAGTAAT TCAAACTCAA ACAATAGGTT TGCCATGCTG
Makindu_cM6      CTGGAGCCAT ATCCAGCCAC AAATAGTAAT TCAAACTCAA ACAATAGGTT TGCCATGCTG
Makindu_1        CTGGAGCCAT ATCCAGCCAC AAATAGTAAT TCAAACTCAA ACAATAGGTT TGCCATGCTA
Amieu_5          CTGGAGCCAT ATCCAGCCAT AAATAGTAAT TCAAACTCAA ACAATAGGTT TGCCATGCTA
Eden_11          CTGGAGCCAT ATCCAGCCAC AAATAGTAAT TCAAACTCAA ACAATAGGTT TGCCATGCTA
Cann-River_10    CTGGAGCCAT ATCCAGCCAC AAATAGTAAT TCAAACTCAA ACAATAGGTT TGCCATGCCA
Brazzaville_10   CTGGAGCCAT ATCCAGCCAC AAATAGTAAT TCAAACTCAA ACAATAGGTT TGCCATGCCA
Cann-River_3     CTGGAGCCAT ATCCAGCCAT AAATAGTAAT TCAAACTCAA ACAATAGGTT TGCCGTGCTA
Cann-River_13    CTGGAGCCAT ATCCAGCCAC AAATAGTAAT TCAAACTCAA ACAATAGGTT TGCCATGCTA
Eden_4           CTGGAGCCAT ATCCAGCCAC AAATAGTAAT TCAAACTCAA ACAATAGGTT TGCCATGCTA
Makindu_10       CTGGAGCCAT ATCCAGCCAC AAATAGTAAT TCAAACTCAA ACAATAGGTT TGCCATGCTA
Makindu_11       CTGGAGCCAT ATCCAGCCAC AAATAGTAAT TCAAACTCAA ACAATAGGTT TGCCATGCTA
Brazzaville_11   CTGGAGCCAT ATCCAGCCAC AAATAGTAAT TCAAACTCAA ACAATAGGTT TGCCATGCTA
Brazzaville_12   CTGGAGCCAT ATCCAGCCAC AAATAGTAAT TCAAACTCAA ACAATAGGTT TGCCATGCTA
Brazzaville_8    CTGGAGCCAT ATCCAGCCAC AAATAGTAAT TCAAACTCAA ACAATAGGTT TGCCATGCTA
chrU_963982      CTGGAGCCAT ATCCAGCCAC AAATAGTAAT TCAAACTCAA ACAATAGGCT TGCCATGCTA
chr3L_18642496   CTGGAGCCAT ATCCAGCCAT AAATAGTAAT TCAAACTCAA ACAATAGGTT TGCCATGCTA
chrU_3975907     CTGGAGCCAT ATCCAGCCAC AAATAGTAAT TCAAACTCAA ACAATAGGTT TGCCATGCTA
chrU_10625041    ---------- ---------- ---------- ---------- ---------- ----------
chrU_2316095     ---------- ---------- ---------- ---------- ---------- ----------
chrU_4537377     ---------- ---------- ---------- ---------- ---------- ----------
chrU_5384045     ---------- ---------- ---------- ---------- ---------- ----------
chrU_5680622     CTGGAGCCAT ATTCAGCCAC AAATAGTAAG TCAAACTCAA ACAATTGGAT GGCCATGCTA
chrU_5950518     ---------- ---------- ---------- ---------- ---------- ----------
chrU_6132643     CTGGAGCCAT ATCCAGCCAT AAATAGTAAT TCAAACTCAA ACAATAGGTT TGCCATGCTA
chrX_16602314    ---------- ---------- ---------- ---------- ---------- ----------
chrU_6536133     CTGGAGCCAT CCTCAGCAAC AATTAGGAAG GCAAACACAA ACAATAAGTT TGCCATGCTA
```

```
                  421
chr3R_1506433     -GACATGGAA TTGGACGAAA CCAGTGATGG CATAGAAACT CCACAAGTTT GTCATGATGT
Brazzaville_16    ---------- ---------- ---------- ---------- ---------- ----------
Eden_3            ---------- ---------- ---------- ---------- ---------- ----------
Cann-River_9      -GACATGGAA TTGGACGAAA CCAGTGATGG CATAGAAACT CCACAAGTTT GTCATGATGT
Amieu_2           TGCCATGGAA TTGGACGAAA CCAGTGATGG CATAGAAACT CCACAAGTTT GTCATGATGC
Makindu_2         -GACATGGAA TTGGACGAAA CCAGTGATGG CATAGAAACT CCACAAGTTT GTCATGATGC
Makindu_6         -GACATGGAA TTGGACGAAA CCAGTGATGG CATAGAAACT CCACAAGTTT GTCATGATGC
Makindu_7         -GACATGGAA TTGGACGAAA CCAGTGATGG CATAGAAACT CCACAAGTTT GTCATGATGC
Valence_6         -GACATGGAA TTGGACGAAA CCAGTGATGG CATAGAAACT CCACAAGTTT GTCATGATGC
Makindu_8         -GACATGGAA TTGGACGAAA CCAGTGATGG CATAGAAACT CCACAAGTTT GTCATGATGC
Makindu_22        -GACATGGAA TTGGACGAAA CCAGTGATGG CATAGAAACT CCACAAGTTT GTCATGATGC
Canberra_5        -GACATGGAA TTGGACGAAA CCAGTGATGG CATAGAAACT CCACAAGTTT GTCATGATGC
Cann-River_2      -GACATGGAA TTGGACGAAA CCAGTGATGG CATAGAAACT CCACAAGTTT GTCATGATGC
Cann-River_6      -GACATGGAA TTGGACGAAA CCAGTGATGG CATAGAAACT CCACAAGTTT GTCATGATGC
Cann-River_7      -GACATGGAA TTGGACGAAA CCAGTGATGG CATAGAAACT CCACAAGTTT GTCATGATGC
Cann_River_5      -GACATGGAA TTGGACGAAA CCAGTGATGG CATAGAAACT CCACAAGTTT GTCATGATGC
Cann-River_8      -GACATGGAA TTGGACGAAA CCAGTGATGG CATAGAAACT CCACAAGTTT GTCATGATGT
Valence_5         -GACATGGAA TTGGACGAAA CCAGTGATGG CATAGAAACT CCACAAGTTT GTCATGATGC
Valence_9         -GACATGGAA TTGGACGAAA CCAGTGATGG CATAGAAACT CCACAAGTTT GTCATGATGC
Eden_1            -GACATGGAA TTGGACGAAA CCAGTGATGG CATAGAAACT CCACAAGTTT GTCATGATGC
Valence_13        -GACATGGAA TTGGACGAAA CCAGTGATGG CATAGAAACT CCACAAGTTT GTCATGATGC
Eden_7            -GACATGGAA TTGGACGAAA CCAGTGATGG CATAGAAACT CCACAAGTTT GTCATGATGC
Valence_4         -GACATGGAA TTGGACGAAA CCAGTGATGG CATAGAAACT CCACAAGTTT GTCATGATGC
Valence_2         -GACATGGAA TTGGACGAAA CCAGTGATGG CATAGAAACT CCACAAGTTT GTCATGATGC
Valence_1         -GACATGGAA TTGGACGAAA CCAGTGATGG CATAGAAACT CCACAAGTTT GTCATGATGC
Valence_7         -GACATGGAA TTGGACGAAA CCAGTGATGG CATAGAAACT CCACAAGTTT GTCATGATGC
Makindu_cM1       -GACATGGAA TTGGACGAAA CCAGTGATGG CATAGAAACT CCACAAGTTT GTCATGATGC
Makindu_cM6       -GACATGGAA TTGGACGAAA CCAGTGATGG CATAGAAACT CCACAAGTTT GTCATGATGC
Makindu_1         -GACATGGAA TTGGACGAAA CCAGTGATGG CATAGAAACT CCACAAGTTT GTCATGATGC
Amieu_5           -GACATGGAA TTGGACGAAA CCAGTGATGG CATAGAAACT CCACAAGTTT GTCATGATGT
Eden_11           -GACATGGAA TTGGACGAAA CCAGTGATGG CATAGAAACT CCACAAGTTT GTCATGATGC
Cann-River_10     TGCCATGGAA TTGGACGAAA CCAGTGATGG CATAGAAACT CCACAAGTTT GTCATGATGC
Brazzaville_10    TGCCATGGAA TTGGACGAAA CCAGTGATGG CATAGAAATT CCACAAGTTT GTCATGATGC
Cann-River_3      -GACATGGAA TTGGACGAAA CCAGTGATGG CATAGAAACT CCACAAGTTC GTCATGATGT
Cann-River_13     -GACATGGAA TTGGACGAAA CCAGTGATGG CATAGAAACT CCACAAGTTT GTCATGATGC
Eden_4            -GACATGGAA TTGGACGAAA CCAGTGATGG CATAGAAACT CCACAAGTTT GTCATGATGC
Makindu_10        -GACATGGAA TTGGACGAAA CCAGTGATGG CATAGAAACT CCACAAGTTT GTCATGATGC
Makindu_11        -GACATGGAA TTGGACGAAA CCAGTGATGG CATAGAAACT CCACAAGTTT GTCATGATGC
Brazzaville_11    -GACATGGAA TTGGACGAAA CCAGTGATGG CATAGAAACT CCACAAGTTT GTCATGATGC
Brazzaville_12    -GACATGGAA TTGGACGAAA CCAGTGATGG CATAGAAACT CCACAAGTTT GTCATGATGC
Brazzaville_8     -GACATGGAA TTGGACGAAA CCAGTGATGG CATAGAAACT CCACAAGTTT GTCATGATGC
chrU_963982       -GACATGGAA TTGGACGAAA CCAGTGATGG CATAGAAACT CTACAAATTT GTCATGATGC
chr3L_18642496    -GACATGGAA TTGGACGAAA CCAGTGATGG CATAGAAACT CCACAAGTTT GTCATGATGT
chrU_3975907      -GACATGGAA TTGGACGAAA CCAGTGATGG CATAGAAACT CCACAAGTTT GTCATGATGC
chrU_10625041     ---------- ---------- ---------- ---------- ---------- ----------
chrU_2316095      ---------- ---------- ---------- ---------- ---------- ----------
chrU_4537377      ---------- ---------- ---------- ---------- ---------- ----------
chrU_5384045      ---------- ---------- ---------- ---------- ---------- ----------
chrU_5680622      -GACATGGAA TTGGACGAAA CCAGTGATGG CATAGAAACT CCACAAGTTT GTAATGATGC
chrU_5950518      ---------- ---------- ---------- ---------- ---------- ----------
chrU_6132643      -GACATGGAA TTGGACGAAA CCAGTGATGG CATAGAAACT CCACAAGTTT GTCATGATGT
chrX_16602314     ---------- ---------- ---------- ---------- ---------- -TCATGATGC
chrU_6536133      -GACATGGAA TTGGACGCAA CCAGTAATGG TATAGAAATT CCACAAGTTT CTAATGATGG
```

```
                481
chr3R_1506433     CGCATCCACC TCGTGTGCAT CGGCTGCTCC GAATATTCCT AATGATGACT GTGTACCAAA
Brazzaville_16    ---------- ---------- ---------- ---------- ---------- ----------
Eden_3            ---------- ---------- ---------- ---------- ---------- ----------
Cann-River_9      CGCATCCACC TCGTGTGCAT CGGCTGCTCC GAATATTCCT AATGATGACT GTGTACCTAA
Amieu_2           CGCATCCACC TCGTGTGCAT CGGCTGCTCC GAATATTCCT AATGATGACT GTGTACCAAA
Makindu_2         CGCATCCACC TCGTGTGCAT CGGCTGCTCC GAAT------ ---------- ----------
Makindu_6         CGCATCCACC TCGTGTGCAT CGGCTGCTCC GAAT------ ---------- ----------
Makindu_7         CGCATCCACC TCGTGTGCAT CGGCTGCTCC GAAT------ ---------- ----------
Valence_6         CGCATCCACC TCGTGTGCAT CGGCTGCTCC GAATATTCCT AATGATGACT GTGTCCCAAA
Makindu_8         CGCATCCACC TCGTGTGCAT CGGCTGCTCC GAAT------ ---------- ----------
Makindu_22        CGCATCCACC TCGTGTGCAT CGGCTGCTCC GAAT------ ---------- ----------
Canberra_5        CGCATCCACC TCGTGTGCAT CGGCTGCTCC GAAT------ ---------- ----------
Cann-River_2      CGCATCCACC TCGTGTGCAT CGGCTGCTCC GAATATTCCT AATGATGACT GTGTCCCAAA
Cann-River_6      CGCATCCACC TCGTGTGCAT CGGCTGCTCC GAATATTCCT AATGATGACT GTGTCCCAAA
Cann-River_7      CGCATCCACC TCGTGTGCAT CGGCTGCTCC GAGTATTCCT AATGATGACT GTGTCCCAAA
Cann_River_5      CGCATCCACC TCGTGTGCAT CGGCTGCTCC GAATATTCCT AATGATGACT GTGTCCCAAA
Cann-River_8      CGCATCCACC TCGTGTGCAT CGGCTGCTCC GAATATTCCT AATGATGACT GTGTACCAAA
Valence_5         CGCATCCACC TCGTGTGCAT CGGCTGCTCC GAATATTCCT AATGATGACT GTGTCCCAAA
Valence_9         CGCATCCACC TCGTGTGCAT CGGCTGCTCC GAATATTCCT AATGATGACT GTGTCCCAAA
Eden_1            CGCATCCACC TCGTGTGCAT CGGCTGCTCC GAATATTCCT AATGATGACT GTGTCCCAAA
Valence_13        CGCATCCACC TCGTGTGCAT CGGCTGCTCC GAATATTCCT AATGATGACT GTGTCCCAAA
Eden_7            CGCATCCACC TCGTGTGCAT CGGCTGCTCC GAATATTCCT AATGATGACT GTGTCCCAAA
Valence_4         CGCATCCACC TCGTGTGCAT CGGCTGCTCC GAATATTCCT AATGATGACT GTGTCCCAAA
Valence_2         CGCATCCACC TCGTGTGCAT CGGCTGCTCC GAATATTCCT AATGATGACT GTGTCCCAAA
Valence_1         CGCATCCACC TCGTGTGCAT CGGCTGCTCC GAATATTCCT AATGATGACT GTGTCCCAAA
Valence_7         CGCATCCACC TCGTGTGCAT CGGCTGCTCC GAATATTCCT AATGATGACT GTGTCCCAAA
Makindu_cM1       CGCATCCACC TCGTGTGCAT CGGCTGCTCC GAATATTCCT AATGATGACT GTGTCCCAAA
Makindu_cM6       CGCATCCACC TCGTGTGCAT CGGCTGCTCC GAATATTCCT AATGATGACT GTGTCCCAAA
Makindu_1         CGCATCCACC TCGTGTGCAT CGGCTGCTCC GAAT------ ---------- ----------
Amieu_5           CGCATCCACC TCGTGTGCAT CGGCTGCTCC GAATATTCCT AATGATAACT GTGTACCAAA
Eden_11           CGCATCCACC TCGTGTGCAT CGGCTGCTCC GAATATTCCT AATGATGACT GTGTACCAAA
Cann-River_10     CGCGTCCACC TCGTGTGCAT CGGCTGCTCC GAATATTCCT AATGATGACT GTGTACCAAA
Brazzaville_10    CGCATCCACC TCGTGTGCAT CGGCTGCTCC GAATATTCCT AATGATGACT GTGTACCAAA
Cann-River_3      CGCATCCACC TCGTGTGCAT CGGCTGCTCC GAATATTCCT AATGATGACT GTGAACCAAA
Cann-River_13     CGCATCCACC TCGTGTGCAC CGGCTGCTCC GAATATTCCT AATGATGACT GTGTACCGAA
Eden_4            CGCATCCACC TCGTGTGCAT CGGCTGCTCC GAATATTCCT AATGATGACT GTGTACCAAA
Makindu_10        CGCATCCACC TCGTGTGCAT CGGCTGCTCC GAATATTCCT AATGATGACT GTGTACCAAA
Makindu_11        CGCATCCACC TCGTGTGCAT CGGCTGCTCC GAATATTCCT AATGATGACT GTGTACCAAA
Brazzaville_11    CGCATCCACC TCGTGTGCAT CGGCTGCTCC GAATATTCCT AATGATGACT GTGTCCCAAA
Brazzaville_12    CGCATCCACC TCGTGTGCAT CGGCTGCTCC GAATATTCCT AATGATGACT GTGTCCCAAA
Brazzaville_8     CGCATCCACC TCGTGTGCAT CGGCTGCTCC GAATATTCCT AATGATGACT GTGTCCCAAA
chrU_963982       CGCATCCACC TCGTGTGCAT CGGCTGCTTC GAATATTCCT AATGATGACT GTGTACCAAA
chr3L_18642496    CGCATCCACC TCGTGTGCAT CGGCTGCT-- ---------- ---------- ----------
chrU_3975907      CGCATCCACC TCGTGTGCAT CGGCTGCTCC GAATATTCCT AATGATGACT GTGTACCAAA
chrU_10625041     ---------- ---------- ---------- ---------- ---------- ----------
chrU_2316095      ---------- ---------- ---------- ---------- ---------- ----------
chrU_4537377      ---------- ---------- ---------- ---------- ---------- ----------
chrU_5384045      ---------- ---------- ---------- ---------- ---------- ----------
chrU_5680622      CGCAGCCACC TCGTGTGCAT CGGCCGCTCC GAATATTCCT AATGATGACT GTGTACCAAA
chrU_5950518      ---------- ---------- ---------- ---------- ---------- ----------
chrU_6132643      CGCATCCACC TCGTGTGCAT CGGCTGCTCC GAATATTCCT AATGATGACT GTGTACCAAT
chrX_16602314     CGCATCCACC TCGTGTGCAT CGGGTGCTCC GAATATTCCT AATGATGACT GTGTACCAAA
chrU_6536133      CGCTGCCACC TTGTGTACAT TGGCCGCTCC AAATATTCCC AATGATGACT GCGTACCAAA
```

```
                  541
chr3R_1506433     TGTGACATCC AATAGCCCAC AACAGTATAC TGATAAGCAG AATTCTTATA AACAATCAAA
Brazzaville_16    ---------- ---------- ---------- ---------- ---------- ----------
Eden_3            ---------- ---------- ---------- ---------- ---------- ----------
Cann-River_9      TGTGACATCC AATAGCCCAC AACAGTATAC TGATAAGCAG AATTCTTATA AACAATCAAA
Amieu_2           TGTGACATCC AATAACCCAC AACAGTATAC TGATAAGCAG AATTCTTATA AACAATCAAA
Makindu_2         ---------- ---------- ---------- ---------- ---------- --CAATCAA-
Makindu_6         ---------- ---------- ---------- ---------- ---------- --CAATCAA-
Makindu_7         ---------- ---------- ---------- ---------- ---------- --CAATCAA-
Valence_6         TGTGACATCC AATTGCCCAC AACAGTATAC TGATAAGCAG AATTCTTATA AACAATCAAA
Makindu_8         ---------- ---------- ---------- ---------- ---------- --CAATCAA-
Makindu_22        ---------- ---------- ---------- ---------- ---------- --CAATCAA-
Canberra_5        ---------- ---------- ---------- ---------- ---------- --CAATCAA-
Cann-River_2      TGTGACATCC AATAGCCCAC AACAGTATAC TGATAAGCAG AATTCTTATA AACAATCAAA
Cann-River_6      TGTGACATCC AATAGCCCAC AACAGTATAC TGATAAGCAG AATTCTTATA AACAATCAAA
Cann-River_7      TGTGACATCC AATAGCCCAC AACAGTATAC TGATAAGCAG AATTCTTATA AACAATCAAA
Cann_River_5      TGTGACATCC AATAGCCCAC AACAGTATAC TGATAAGCAG AATTCTTATA AACAATCAAA
Cann-River_8      TGTGACATCC AATAGCCCAC AACAGTATAC TGATAAGCAG AATTCTTATA AACAATCAAA
Valence_5         TGTGACATCC AATTGCCCAC AACAGTATAC TGATAAGCAG AATTCTTATA AACAATCAAA
Valence_9         TGTGACATCC AATTGCCCAC AACAGTATAC TGATAAGCAG AATTCTTATA AACAATCAAA
Eden_1            TGTGACATCC AATAGCCCAC AACAGTATAC TGATAAGCAG AACTCTTATA AACAATCAAA
Valence_13        TGTGACATCC AATTGCCCAC AACAGTATAC TGATAAGCAG AATTCTTATA AACAATCAAA
Eden_7            TGTGACATCC AATAGCCCAC AACAGTATAC TGATAAGCAG AACTCTTATA AACAATCAAA
Valence_4         TGTGACATCC AATTGCCCAC AACAGTATAC TGATAAGCAG AATTCTTATA AACAATCAAA
Valence_2         TGTGACATCC AATTGCCCAC AACAGTATAC TGATAAGCAG AATTCTTATA AACAATCAAA
Valence_1         TGTGACATCC AATTGCCCAC AACAGTATAC TGATAAGCAG AATTCTTATA AACAATCAAA
Valence_7         TGTGACATCC AATTGCCCAC AACAGTATAC TGATAAGCAG AATTCTTATA AACAATCAAA
Makindu_cM1       TGTGACATCC AATTGCCCAC AACAGTATAC TGATAAGCAG AATTCTTATA AACAATCAAA
Makindu_cM6       TGTGACATCC AATTGCCCAC AACAGTATAC TGATAAGCAG AATTCTTATA AACAATCAAA
Makindu_1         ---------- ---------- ---------- ---------- ---------- --CAATCAA-
Amieu_5           TGTGACATCC AATAGCCCAC AACAGTATAC TGATAAGCAG AATTCTTATA AACAATCAAA
Eden_11           TGTGACATCC AATAGCCGAC AACAGTATAC TGATAAGCAG AATTCTTATA AACAATCAAA
Cann-River_10     TGTGACATCC AATAACCCAC AACAGTATAC TGATAAGCAG AATTCTTATA AACAATCAAA
Brazzaville_10    TGTGACATCC AATAACCCAC AACAGTATAC TGATAAGCAG AATTCTTATA AACAATCAAA
Cann-River_3      TGTGACATCC AATAGCCCAC AACAGTATAC TGATAAGCAG AATTCTTATA AACAATCAAA
Cann-River_13     TGTGACATCC AATAGCCGAC AACAGTATAC TGATAAGCAG AATTCTTATA AACAATCAAA
Eden_4            TGTGACTTCC AATAGCCGAC AACAGTATAC TGATAAGCAG AATTCTTATA AACAATCAAA
Makindu_10        TGTGACATCC AATAGCCGAC AACAGTATAC TGATAAGCAG AATTCTTATA AACAATCAAA
Makindu_11        TGTGACATCC AATAGCCGAC AACAGTATAC TGATAAGCAG AATTCTTATA AACAATCAAA
Brazzaville_11    TGTGACATCC AATAGCCCAC AACAGTATAC TGATAAGCAG AATTCTTATA AACAATCAAA
Brazzaville_12    TGTGACATCC AATAGCCCAC AACAGTATAC TGATAAGCAG AATTCTTATA AACAATCAAA
Brazzaville_8     TGTGACATCC AATAGCCCAC AACAGTATAC TGATAAGCAG AATTCTTATA AACAATCAAA
chrU_963982       TGTGACATCC AATAGCCCAC AACAGAATAC TGATAAGCAG AATTGCTATA AACAATCAAA
chr3L_18642496    ---------- ---------- ---------- ---------- ---------- ----------
chrU_3975907      TGTGACATCC AATAACCCAC AACAGTATAC TGATAAGCAG AATTCTTATA AACAATCAAA
chrU_10625041     ---------- ---------- ---------- ---------- ---------- ----------
chrU_2316095      ---------- ---------- ---------- ---------- ---------- ----------
chrU_4537377      ---------- ---------- ---------- ---------- ---------- ----------
chrU_5384045      ---------- ---------- ---------- ---------- ---------- ----------
chrU_5680622      TGTGACATCC ACTAGCCCAC AACAGTATAC TGATAAGCAG AATCCTTATA AACAATCAAA
chrU_5950518      ---------- ---------- ---------- ---------- ---------- ----------
chrU_6132643      TGTGACATCC AATAGCCCAC AACAGTATAC TGATTAACAA AATTCTTATA AACAATCAAA
chrX_16602314     TGTGACATCC AATAGCCCAC AACAGAATAC TGATAAGCAG AATTGCTATA AACAATCAAA
chrU_6536133      TAAGACATCC AACAGACCAC AACAGTATAC TGATAAGAAG AATTCTTATA AACAATCAAA
```

```
                 601
chr3R_1506433    ACCACCGCAA ATAGTACTGA GCCTTACCAA TCTTAATGAT CTCTATGAGC TCATTACGGA
Brazzaville_16   ---------- ---------- ---------- ---------- ---------- ----------
Eden_3           ---------- ---------- ---------- ---------- -TTTTTGAGC TCATTACGGA
Cann-River_9     ACCACCGCAA ATAGTACTGA GCCTTACCAA CCTTAATGAT CTTTTTGAGC TCATTACGGA
Amieu_2          ACCACCGCAA ATAGTACTGA GCCTTACCAA TCTTAATGAT CTCTATGAGC TCATTACGGA
Makindu_2        ---------- ---------- ---------- ---------- ---------- ----------
Makindu_6        ---------- ---------- ---------- ---------- ---------- ----------
Makindu_7        ---------- ---------- ---------- ---------- ---------- ----------
Valence_6        ACCACCGCAA ATAGTACTGA GCCTTACCAA CCTTAATGAT CTCTTTGAGC TCATTACGGA
Makindu_8        ---------- ---------- ---------- ---------- ---------- ----------
Makindu_22       ---------- ---------- ---------- ---------- ---------- ----------
Canberra_5       ---------- ---------- ---------- ---------- ---------- ----------
Cann-River_2     ACCACCGCAA ATAGTACTGA GCCTTACCAA CCTTAATGAT CTCTTTGAGC TCATTACGGA
Cann-River_6     ACCACCGCAA ATAGTACTGA GCCTTACCAA CCTTAATGAT CTCTTTGAGC TCATTACGGA
Cann-River_7     ACCACCGCAA ATAGTACTGA GCCTTACCAA CCTTAATGAT CTCTTTGAGC TCATTACGGA
Cann_River_5     ACCACCGCAA ATAGTACTGA GCCTTACCAA CCTTAATGAT CTCTTTGAGC TCATTACGGA
Cann-River_8     ACCACCGCAA ATAGTACTGA GCCTTACCAA TCTTAATGAT CTCTATGAGC TCATTACGGA
Valence_5        ACCACCGCAA ATAGTACTGA GCCTTACCAA CCTTAATGAT CTCTTTGAGC TCATTACGGA
Valence_9        ACCACCGCAA ATAGTACTGA GCCTTACCAA CCTTAATGAT CTCTTTGAGC TCATTACGGA
Eden_1           ACCACCGCAA ATAGTACTGA GCCTTACCAA CCTTAATGAT CTCTTTGAGC TCATTACGGA
Valence_13       ACCACCGCAA ATAGTACTGA GCCTTACCAA CCTTAATGAT CTCTTTGAGC TCATTACGGA
Eden_7           ACCACCGCAA ATAGTACTGA GCCTTACCAA CCTTAATGAT CTCTTTGAGC TCATTACGGA
Valence_4        ACCACCGCAA ATAGTACTGA GCCTTACCAA CCTTAATGAT CTCTTTGAGC TCATTACGGA
Valence_2        ACCACCGCAA ATAGTACTGA GCCTTACCAA CCTTAATGAT CTCTTTGAGC TCATTACGGA
Valence_1        ACCACCGCAA ATAGTACTGA GCCTTACCAA CCTTAATGAT CTCTTTGAGC TCATTACGGA
Valence_7        ACCACCGCAA ATAGTACTGA GCCTTACCAA CCTTAATGAT CTCTTTGAGC TCATTACGGA
Makindu_cM1      ACCACCGCAA ATAGTACTGA GCCTTACCAA CCTTAATGAT CTCTTTGAGC TCATTACGGA
Makindu_cM6      ACCACCGCAA ATAGTACTGA GCCTTACCAA CCTTAATGAT CTCTTTGAGC TCATTACGGA
Makindu_1        ---------- ---------- ---------- ---------- ---------- ----------
Amieu_5          ACCACCGCAA ATAGTACTGA GCCTTACCAA TCTTAATGAT CTCTATGAGC TCATTACGGA
Eden_11          ACCACCGCAA ATAGTACTGA GCCTTACCAA CCTTAATGAT CTCTTTCAGC TCATTACGGA
Cann-River_10    ACCACCGCAA ATAGTACTGA GCCTTACCAG CCTTAATGAT CTCTTTGAGC TCATTACTGA
Brazzaville_10   ACCACCGCAA ATAGTACTGA GCCTTACCAG CCTTAATGAT CTCTTTGAGC TCATTACTGA
Cann-River_3     ACCACCGCAA ATAGTACTGA GCCTTACCAA TCTTAATGAT CTCTATGAGC TCATTACGGA
Cann-River_13    ACCACCGCAA ATAGTACTGA GCCTTACCAA CCTTAATGAT CTCTTTCAGC TCATTACGGA
Eden_4           ACCACCGCAA ATAGTACTGA GCCTTACCAA CCTTAATGAT CTCTTTCAGC TCATTACGGA
Makindu_10       ACCACCGCAA ATAGTACTGA GCCTTACCAA CCTTAATGAT CTCTTTCAGC TCATTACGGA
Makindu_11       ACCACCGCAA ATAGTACTGA GCCTTACCAA CCTTAATGAT CTCTTTCAGC TCATTACGGA
Brazzaville_11   ACCACCGCaa ATAGTACTGA GCCTTACCAA CCTTAATGAT CTCTTTGAGC TCATTACGGA
Brazzaville_12   ACCACCGCAA ATAGTACTGA GCCTTACCAA CCTTAATGAT CTCTTTGAGC TCATTACGGA
Brazzaville_8    ACCACCGCAA ATAGTACTGA GCCTTACCAA CCTTAATGAT CTCTTTGAGC TCATTACGGA
chrU_963982      ACCACCGCAA ATAGTACTGA GCCTTACCAA CCTTAATGAT CTCTTTGAGC TCATTACGGA
chr3L_18642496   ---------- ---------- ---------- ---------- ---------- ----------
chrU_3975907     ACCACCGCAA ATAGTACTGA CCCTTACCAA CCTTAATGAT CTCTTTGAGC TCATTACGGA
chrU_10625041    ---------- ---------- ---------- ---------- ---------- ----------
chrU_2316095     ---------- ---------- ---------- ---------- ---------- ----------
chrU_4537377     ---------- ---------- ---------- ---------- -----TGAGC TCATTACGGA
chrU_5384045     ---------- ---------- ---------- ---------- ---------- ----------
chrU_5680622     ACCACCGCAA ATAGTACTGA GCCTTACTAA CCTTAATGAA CTCTTTGAGC TCATTACG-A
chrU_5950518     ---------- ---------- ---------- ---------- ---------- ----------
chrU_6132643     ACCACC---- ---------- ---------- ---------- ---------- ----------
chrX_16602314    ACCACCGCAA ATAGTACTGA GCCTTACCAA CCTTAATGAT CTCTTTGAGC TCATTACGGA
chrU_6536133     ACCACCGCAA ATAGAACTGC GAATTACCAA CCTTAATGAT CTCTTTG--C TCATTACGGA
```

```
                661
chr3R_1506433     GGT-CACTAG CCTAGATAAT TTAACAGTTA AAGTCAATCA AGGGGAAACA GTGAGAATAT
Brazzaville_16    ---------- ---------- ---------- ---------- ---------- ----------
Eden_3            GGC-CACTAA CCTAGATAAT TTAACAGTTA AAGTCAATCA AGGAGAAACA GTGAGAATAT
Cann-River_9      GGC-CACTAA CCTAGATAAT TTAACAGTTA AAGTCAATCA AGGAGAAACA GTGAGAATAT
Amieu_2           GGT-CACTAG CCTAGATAAT TTAACAGTTA AAGTCAATCA AGGGGAAACA GTGAGAATAT
Makindu_2         ---------- ---------- ---------- --GTCAATCA AGGGGAAACA GTGAGAATAT
Makindu_6         ---------- ---------- ---------- --GTCAATCA AGGGGAAACA GTGAGAATAT
Makindu_7         ---------- ---------- ---------- --GTCAATCA AGGGGAAACA GTGAGAATAT
Valence_6         GGT-CACTAG CCTAGATAAT TTAACAGTTA AAGTCAATCA AGGGGAAACA GTGAGAATAT
Makindu_8         ---------- ---------- ---------- --GTCAATCA AGGGGAAACA GTGAGAATAT
Makindu_22        ---------- ---------- ---------- --GTCAATCA AGGGGAAACA GTGAGAATAT
Canberra_5        ---------- ---------- ---------- --GTCAATCA AGGGGAAACA GTGAGAATAT
Cann-River_2      GGT-CACTAG CCTAGATAAT TTAACAGTTA AAGTCAATCA AGGGGAAACA GTGAGAATAT
Cann-River_6      GGT-CACTAG CCTAGATAAT TTAACAGTTA AAGTCAATCA AGGGGAAACA GTGAGAATAT
Cann-River_7      GGT-CACTAG CCTAGATAAT TTAACAGTTA AAGTCAATCA AGGGGAAACA GTGAGAATAT
Cann_River_5      GGT-CACTAG CCTAGATAAT TTAACAGTTA AAGTCAATCA AGGGGAAACA GTGAGAATAT
Cann-River_8      GGT-CACTAG CCTAGATAAT TTAACAGTTA AAGTCAATCA AGGGGAAACA GTGAGAATAT
Valence_5         GGT-CACTAG CCTAGATAAT TTAACAGTTA AAGTCAATCA AGGGGAAACA GTGAGAATAT
Valence_9         GGT-CACTAG CCTAGATAAT TTAACAGTTA AAGTCAATCA AGGGGAAACA GTGAGAATAT
Eden_1            GGT-CACTAG CCTAGATAAT TTAACAGTTA AAGTCAATCA AGGGGAAACA GTGAGAATAT
Valence_13        GGT-CACTAG CCTAGATAAT TTAACAGTTA AAGTCAATCA AGGGGAAACA GTGAGAATAT
Eden_7            GGT-CACTAG CCTAGATAAT TTAACAGTTA AAGTCAATCA AGGGGAAACA GTGAGAATAT
Valence_4         GGT-CACTAG CCTAGATAAT TTAACAGTTA AAGTCAATCA AGGGGAAACA GTGAGAATAT
Valence_2         GGT-CACTAG CCTAGATAAT TTAACAGTTA AAGTCAATCA AGGGGAAACA GTGAGAATAT
Valence_1         GGT-CACTAG CCTAGATAAT TTAACAGTTA AAGTCAATCA AGGGGAAACA GTGAGAATAT
Valence_7         GGT-CACTAG CCTAGATAAT TTAACAGTTA AAGTCAATCA AGGGGAAACA GTGAGAATAT
Makindu_cM1       GGT-CACTAG CCTAGATAAT TTAACAGTTA AAGTCAATCA AGGGGAAACA GTGAGAATAT
Makindu_cM6       GGT-CACTAG CCTAGATAAT TTAACAGTTA AAGTCAATCA AGGGGAAACA GTGAGAATAT
Makindu_1         ---------- ---------- ---------- --GTCAATCA AGGGGAAACA GTGAGAATAT
Amieu_5           GGT-CACTAG CCTAGATAAT TTAACAGTTA AAGTCAATCA AGGGGAAACA GTGAGAATAT
Eden_11           GGT-CACTAG CCTAGATAAT TTAACAGTTA AAGTCAATCA AGGAAAAACA GTGAGAATAT
Cann-River_10     GGT-CACTAG CATAGATAAT TTAACAGTTA ATGTCAATCA AGGGGAAGCA GTGAGAATAT
Brazzaville_10    GGT-CACTAG CATAGATAAT TTAACAGTTA ATGTCAATCA AGGGGAAGCA GTGAGAATAT
Cann-River_3      GGT-CACTAG CCTAGATAAT TTAACAGTTA AAGTCAATCA AGGGGAAACA GTGAGAATAT
Cann-River_13     GGT-CACTAG CCTAGATAAT TTAACAGTTA AAGTCAATCA AGGAAAAACA GTGAGAATAT
Eden_4            GGT-CACTAG CATAGATAAT TTAACAGTTA AAGTCAATCA AGGAAAAACA GTGAGAATAT
Makindu_10        GGT-CACTAG CCTAGATAAT TTAACAGTTA AAGTCAATCA AGGAAAAACA GTGAGAATAT
Makindu_11        GGT-CACTAG CCTAGATAAT TTAACAGTTA AAGTCAATCA AGGAAAAACA GTGAGAATAT
Brazzaville_11    gGT-CACTAG CCTAGATaAT TTAACAGTTA AAGTCAATCA AGGGGAAACA GTGAGAATAT
Brazzaville_12    GGT-CACTAG CCTAGATAAT TTAACAGTTA AAGTCAATCA AGGGGAAACA GTGAGAATAT
Brazzaville_8     GGT-CACTAG CCTAGATAAT TTAACAGTTA AAGTCAATCA AGGGGAAACA GTGAGAATAT
chrU_963982       GGT-CACTAG CCTAGATAAT TTAACAGTTA AAGTCAATCA AGGAGAAACA GTGAGAATAA
chr3L_18642496    ---------- ---------- ---------- ---------- ---------- ----------
chrU_3975907      GGT-CACTAG CATAAATAAT TTAACAGTTA AAGTCAATCA AGGGGA---- ----------
chrU_10625041     ---------- ---------- ---------- ---------- ---------- ----------
chrU_2316095      ---------- ---------- ---------- ---------- ---------- ----------
chrU_4537377      GGTTCATCAC CCTAGGTAAT TAACCAGTAA AGGTCAATCA GGGGGAAACA GGAGAAATTT
chrU_5384045      ---------- ---------- ---------- ---------- ---------- ----------
chrU_5680622      GAT-CACTAG CCTAGATA-- ---------- ---------- ---------- ----------
chrU_5950518      ---------- ---------- ---------- ---------- ---------- ----------
chrU_6132643      ---------- ---------- ---------- ---------- ---------- ----------
chrX_16602314     GGT-CACTAG ACTAGATAAT TTAACAGTTA AAGTCAATCA AGGAGAAACA GTGAGAATAT
chrU_6536133      GGT-CACTAG CCTAGATAAT ATAA------ ---------- ---------- ----GAATAT
```

```
                 721
chr3R_1506433    TACCCAAA-G ACTCTGATAC TTACAGAGCT ATTGTTAATC TTTTTGATAA TTCGGGAATT
Brazzaville_16   ---------- ---------- ---------- ---------- ------ATAA TTCGGGAATT
Eden_3           TACCCAAA-G ACGCTGATAC TTATAGAGCC ATTGTTAATC TTTTGGATAA TTCGGGAATT
Cann-River_9     TACCCAAA-G ACGCTGATAC TTATAGAGCC ATTGTTAATC TTTTGGATAA TTCGGGAATT
Amieu_2          TACCCAAA-G ACTCTGATAC TTACAGAGCT ATTGTTAATC TTTTTGATAA TTCGGGAATT
Makindu_2        TACCCAAA-G ACGCTGATAC TTATAGAGCC ATTGTGAATC GTTTGGATAA TTCGGGAATT
Makindu_6        TACCCAAA-G ACGCTGATAC TTATAGAGCC ATTGTGAATC GTTTGGATAA TTCGGGAATT
Makindu_7        TACCCAAA-G ACGCTGATAC TTATAGAGCC ATTGTGAATC GTTTGGATAA TTCGGGAATT
Valence_6        TACCCAAA-G ACGCTGATAC TTATAGAGCC ATTGTGAATC TTTTGGATAA TTCGGGAATT
Makindu_8        TACCCAAA-G ACGCTGATAC TTATAGAGCC ATTGTGAATC GTTTGGATAA TTCGGGAATT
Makindu_22       TACCCAAA-G ACGCTGATAC TTATAGAGCC ATTGTGAATC GTTTGGATAA TTCGGGAATT
Canberra_5       TACCCAAA-G ACGCTGATAC TTATAGAGCC ATTGTGAATC GTTTGGATAA TTCGGGAATT
Cann-River_2     TACCCAAA-G ACGCTGATAC TTATAGAGCC ATTGTGAATC GTTTGGATAA TTCGGGAATT
Cann-River_6     TACCCAAA-G ACGCTGATAC TTATAGAGCC ATTGTGAATC GTTTGGATAA TTCGGGAATT
Cann-River_7     TACCCAAA-G ACGCTGATAC TTATAGAGCC ATTGTGAATC GTTTGGATAA TTCGGGAATT
Cann_River_5     TACCCAAG-G ACGCTGATAC TTATAGAGCC ATTGTGAATC GTTTGGATAA TTCGGGAATT
Cann-River_8     TACCCAAA-G ACTCTGATAC TTACAGAGCT ATTGTTAATC TTTTTGATAA TTCGGGAATT
Valence_5        TACCCAAA-G ACGCTGATAC TTATAGAGCC ATTGTGAATC TTTTGGATAA TTCGGGAATT
Valence_9        TACCCAAA-G ACGCTGATAC TTATAGAGCC ATTGTGAATC TTTTGGATAA TTCGGGAATT
Eden_1           TACCCAAA-G ACGCTGATAC TTATAGAGCC ATTGTGAATC GTTTGGATAA TTCGGGAATT
Valence_13       TACCCAAA-G ACGCTGATAC TTATAGAGCC ATTGTGAATC TTTTGGATAA TTCGGGAATT
Eden_7           TACCCAAA-G ACGCTGATAC TTATAGAGCC ATTGTGAATC GTTTGGATAA TTCGGGAATT
Valence_4        TACCCAAA-G ACGCTGATAC TTATAGAGCC ATTGTGAATC TTTTGGATAA TTCGGGAATT
Valence_2        TACCCAAA-G ACGCTGATAC TTATAGAGCC ATTGTGAATC TTTTGGATAA TTCGGGAATT
Valence_1        TACCCAAA-G ACGCTGATAC TTATAGAGCC ATTGTGAATC TTTTGGATAA TTCGGGAATT
Valence_7        TACCCAAA-G ACGCTGATAC TTATAGAGCC ATTGTGAATC TTTTGGATAA TTCGGGAATT
Makindu_cM1      TACCCAAA-G ACGCTGATAC TTATAGAGCC ATTGTGAATC TTTTGGATAA TTCGGGAATT
Makindu_cM6      TACCCAAA-G ACGCTGATAC TTATAGAGCC ATTGTGAATC TTTTGGATAA TTCGGGAATT
Makindu_1        TACCCAAA-G ACGCTGATAC TTATAGAGCC ATTGTGAATC GTTTGGATAA TTCGGGAATT
Amieu_5          TACCCAAA-G ACTCTGATAC TTACAGAGCT ATTGTTAATC TTTTTGATAA TTCGGGAATT
Eden_11          TACCCAAA-G ACGCTGATAC TTATAGAGCC ATTGTTAATC TTTTGGATAA TTCGGGAATT
Cann-River_10    TACCCAAA-G ACGCTGATAC TTATAGAGCC ATTGTTAATC TTTTGGATAA TTCGGGAATT
Brazzaville_10   TACCCAAA-G ACGCTGATAC TTATAGAGCC ATTGTTAATC TTTTGGATAA TTCGGGAATT
Cann-River_3     TGCCCAAA-G ACTCTGATAC TTGCAGAGCT ATTGTTAATC TTTTTGTTAA TTCGGGAATT
Cann-River_13    TACCCAAA-G ACGCTGATAC TTATAGAGCC ATTGTTAATC TTTTGGATAA TTCGGGAATT
Eden_4           TACCCAAA-G ACGCTGATAC TTATAGAGCC ATTGTTAATC TTTTGGATAA TTCGGGAATT
Makindu_10       TACCCAAA-G ACGCTGATAC TTATAGAGCC ATTGTTAATC TTTTGGATAA TTCGGGAATT
Makindu_11       TACCCAAA-G ACGCTGATAC TTATAGAGCC ATTGTTAATC TTTTGGATAA TTCGGGAATT
Brazzaville_11   TACCCAAA-G ACGCTGATAC TTATAGAGCC ATTGTGAATC GTTTGGATAA TTCGGGAATT
Brazzaville_12   TACCCAAA-G ACGCTGATAC TTATAGAGCC ATTGTGAATC GTTTGGATAA TTCGGGAATT
Brazzaville_8    TACCCAAA-G ACGCTGATAC TTATAGAGCC ATTGTGAATC GTTTGGATAA TTCGGGAATT
chrU_963982      TACCCAAA-G ACGCTGATAC TTATAGAACC ATTGTTAATC TTTTGGATAA TTCGGGAATT
chr3L_18642496   ---------- ---------- ---------- ---------- ---------- ----------
chrU_3975907     ---------- ---------- ---------- ---------- ---------- ----------
chrU_10625041    ---------- ---------- ---------- ---------- ---------- ----------
chrU_2316095     ---------- ---------- ---------- ---------- ---------- ----------
chrU_4537377     TACCCAAAAG ATTCTGAAAT TACCAGAGTT ATTGTTATTG TTTTTGATAA TTCGGTAAGT
chrU_5384045     ---------- ---------- ---------- ---------- ---------- ----------
chrU_5680622     ---------- ---------- ---------- ---------- ---------- ----------
chrU_5950518     ---------- ---------- ---------- ---------- ---------- ----------
chrU_6132643     ---------- ---------- ---------- ---------- ---------- ----------
chrX_16602314    TACCC----- ---------- ---------- ---------- ---------- ----------
chrU_6536133     TTCCCAAA-G ACGTTGATAC TTATAGAGCC ATTGTTAATT CTTTGGATAG TTCGGGAATT
```

```
                781
chr3R_1506433    GAATTCCATA CGTACCAAAT GAAGGAAGAG AAGCCTCACA GAATAGTTGT TAAGGGACTC
Brazzaville_16   GAATTCCATA CGTACCAAAT GAAGGAAGAG AAGTCTCACA AAATAGTTGT TAAGGGACTC
Eden_3           GAGTTCCATA CGTACCAAAT TAAGGATGAG AAGCCTCACA GAATAGTTGT TAAGGGACTC
Cann-River_9     GAGTTCCATA CGTACCAAAT TAAGGATGAG AAGCCTCACA GAATAGTTGT TAAGGGACTC
Amieu_2          GAATTCCATA CgTACCAAAT GAAGGAAGAG AAGCCTCACA GAATAGTTGT TAAGGGACTC
Makindu_2        GAATTCCATA CGTACCAAAT GAAGGAAGAG AAGTCTCACA AAATAGTTGT TAAGGGACTC
Makindu_6        GAATTCCATA CGTACCAAAT GAAGGAAGAG AAGTCTCACA AAATAGTTGT TAAGGGACTC
Makindu_7        GAATTCCATA CGTACCAAAT GAAGGAAGAG AAGTCTCACA AAATAGTTGT TAAGGGACTC
Valence_6        GAATTCCATA CGTACCAAAT GAAGGAAGAG AAGTCTCACA AAATAGTTGT TAAGGGACTC
Makindu_8        GAATTCCATA CGTACCAAAT GAAGGAAGAG AAGTCTCACA AAATAGTTGT TAAGGGACTC
Makindu_22       GAATTCCATA CGTACCAAAT GAAGGAAGAG AAGTCTCACA AAATAGTTGT TAAGGGACTC
Canberra_5       GAATTCCATA CGTACCAAAT GAAGGAAGAG AAGTCTCACA AAATAGTTGT TAAGGGACTC
Cann-River_2     GAATTCCATA CGTACCAAAT GAAGGAAGAG AAGTCTCACA AAATAGTTGT TAAGGGACTC
Cann-River_6     GAATTCCATA CGTACCAAAT GAAGGAAGAG AAGTCTCACA AAATAGTTGT TAAGGGACTC
Cann-River_7     GAATTCCATA CGTACCAAAT GAAGGAAGAG AAGTCTCACA AAATAGTTGT TAAGGGACTC
Cann_River_5     GAATTCCATA CGTACCAAAT GAAGGAAGAG AAGTCTCACA AAATAGTTGT TAAGGGACTC
Cann-River_8     GAATTCCATA CGTACCAAAT GAAGGAAGAG AAGCCTCACA GAATAGTTGT TAAGGGACTC
Valence_5        GAATTCCATA CGTACCAAAT GAAGGAAGAG AAGTCTCACA AAATAGTTGT TAAGGGACTC
Valence_9        GAATTCCATA CGTACCAAAT GAAGGAAGAG AAGTCTCACA AAATAGTTGT TAAGGGACTC
Eden_1           GAATTCCATA CGTACCAAAT GAAGGAAGAG AAGTCTCACA AAATAGTTGT TAAGGGACTC
Valence_13       GAATTCCATA CGTACCAAAT GAAGGAAGAG AAGTCTCACA AAATAGTTGT TAAGGGACTC
Eden_7           GAATTCCATA CGTACCAAAT GAAGGAAGAG AAGTCTCACA AAATAGTTGT TAAGGGACTC
Valence_4        GAATTCCATA CGTACCAAAT GAAGGAAGAG AAGTCTCACA AAATAGTTGT TAAGGGACTC
Valence_2        GAATTCCATA CGTACCAAAT GAAGGAAGAG AAGTCTCACA AAATAGTTGT TAAGGGACTC
Valence_1        GAATTCCATA CGTACCAAAT GAAGGAAGAG AAGTCTCACA AAATAGTTGT TAAGGGACTC
Valence_7        GAATTCCATA CGTACCAAAT GAAGGAAGAG AAGTCTCACA AAATAGTTGT TAAGGGACTC
Makindu_cM1      GAATTCCATA CGTACCAAAT GAAGGAAGAG AAGTCTCACA AAATAGTTGT TAAGGGACTC
Makindu_cM6      GAATTCCATA CGTACCAAAT GAAGGAAGAG AAGTCTCACA AAATAGTTGT TAAGGGACTC
Makindu_1        GAATTCCATA CGTACCAAAT GAAGGAAGAG AAGTCTCACA AAATAGTTGT TAAGGGACTC
Amieu_5          GAATTCCATA CGTACCAAAT GAAGGAAGAG AAGCCTCACA GAATAGTTGT TAAGGGACTC
Eden_11          GAATTCCATA CGTACCAAAT GAAGGAAGAG AAGCCTCACA GAATAGTTGT TAAGGGACTC
Cann-River_10    GAATTCCATA CGTACCAAAT GAAGGAAGAG AAGCCTCACA GAATAGTTGT TAAGGGACTC
Brazzaville_10   GAATTCCATA CGTACCAAAT GAAGGAAGAG AAGCCTCACA GAATAGTTGT TAAGGGACTC
Cann-River_3     GAATTCCATA CGTACCAAAT GAAGGAAGAG AAGCCTCACA GAATAGTTGT TAAGGGACCC
Cann-River_13    GAATTCCATA CGTACCAAAT GAAGGAAGAG AAGCCTCACA GAATAGTTGT TAAGGGACTC
Eden_4           GAATTCCATA CGTACCAAAT GAAGGAAGAG AAGCCTCACA GAATAGTTGT TAAGGGACTC
Makindu_10       GAATTCCATA CGTACCAAAT GAAGGAAGAG AAGCCTCACA GAATAGTTGT TAAGGGACTC
Makindu_11       GAATTCCATA CGTACCAAAT GAAGGAAGAG AAGCCTCACA GAATAGTTGT TAAGGGACTC
Brazzaville_11   GAATTCCATA CGTACCAAAT GAAGGAAGAG AAGTCTCACA AAATAGTTGT TAAGGGACTC
Brazzaville_12   GAATTCCATA CGTACCAAAT GAAGGAAGAG AAGTCTCACA AAATAGTTGT TAAGGGACTC
Brazzaville_8    GAATTCCATA CGTACCAAAT GAAGGAAGAG AAGTCTCACA AAATAGTTGT TAAGGGACTC
chrU_963982      GAATTCCATA CGTACCAAAT GAAGGAAGAG AAGCCTCACA GAATAGTTGT TAAGGGACTT
chr3L_18642496   ---------- ---------- ---------- ---------- ---------- ----------
chrU_3975907     ---------- ---------- ---------- ---------- ---------- ----------
chrU_10625041    ---------- ---------- ---------- ---------- ---------- ----------
chrU_2316095     ---------- ---------- ---------- ---------- ---------- ----------
chrU_4537377     GAATTCCATA CGTACCAAAT GAAGGAAGAG AAGCCTCACA GAGTAGTTGT TAAGGGACTC
chrU_5384045     ---------- ---------- ---------- ---------- ---------- ----------
chrU_5680622     ---------- ---------- ---------- ---------- ---------- ----------
chrU_5950518     ---------- ---------- ---------- ---------- ---------- ----------
chrU_6132643     ---------- ---------- ---------- ---------- ---------- ----------
chrX_16602314    ---------- ---------- ---------- ---------- ---------- ----------
chrU_6536133     GAATTCCATA CGTAC----- ---------- ---------- ---------- ----------
```

```
                   841
chr3R_1506433      CACCATAGCA CCCTAACCTA CGAAATTATC GACAACTTTA AAACATATGG -CTTTGATGT
Brazzaville_16     CACCATGGCA CCCTAACCTA CGAAATTATC GACAACTTTA AAACATATGG -CTTTGATGT
Eden_3             CACCATAGCA CCCTAACCTA CGAAATTATC GACAACTTTA AAACATATGG -CTTTGATAT
Cann-River_9       CACCATAGCA CCCTAACCTA CGAAATTATC GACAACTTTA AAACATATGG -CTTTGATAT
Amieu_2            CACCATAGCA CCCTAACCTA CGAAATTATC GACAACTTTA AAACATATGG -CTTTGATGT
Makindu_2          CACCATGGCA CCCTAACCTA CGAAATTATC GACAACTTTA AAACATATGG -CTTTGATGT
Makindu_6          CACCATGGCA CCCTAACCTA CGAAATTATC GACAACTTTA AAACATATGG -CTTTGATGT
Makindu_7          CACCATGGCA CCCTAACCTA CGAAATTATC GACAACTTTA AAACATATGG -CTTTGATGT
Valence_6          CACCATGGCA CCCTAACCTA CGAAATTATC GACAACTTTA AAACATATGG -CTTTGATGT
Makindu_8          CACCATGGCA CCCTAACCTA CGAAATTATC GACAACTTTA AAACATATGG -CTTTGATGT
Makindu_22         CACCATGGCA CCCTAACCTA CGAAATTATC GACAACTTTA AAACATATGG -CTTTGATGT
Canberra_5         CACCATGGCA CCCTAACCTA CGAAATTATC GACAACTTTA AAACATATGG -CTTTGATGT
Cann-River_2       CACCATGGCA CCCTAACCTA CGAAATTATC GACAACTTTA AAACATATGG -CTTTGATGT
Cann-River_6       CACCATGGCA CCCTAACCTA CGAAATTATC GACAACTTTA AAACATATGG -CTTTGATGT
Cann-River_7       CACCATGGCA CCCTAACCTA CGAAATTATC GACAACTTTA AAACATATGG -CTTTGATGT
Cann_River_5       CACCATGGCA CCCTAACCTA CGAAATTATC GACAACTTTA AAACATATGG -CTTTGATGT
Cann-River_8       CACCATAGCA CCCTAACCTA CGAAATTATC GACAACTTTA AA-CATATGG -CTTTGATGT
Valence_5          CACCATGGCA CCCTAACCTA CGAAATTATC GACAACTTTA AAACATATGG -CTTTGATGT
Valence_9          CACCATGGCA CCCTAACCTA CGAAATTATC GACAACTTTA AAACATATGG -CTTTGATGT
Eden_1             CACCATGGCA CCCTAACCTA CGAAATTATC GACAACTTTA AAACATATGG -CTTTGATGT
Valence_13         CACCATGGCA CCCTAACCTA CGAAATTATC GACAACTTTA AAACATATGG -CTTTGATGT
Eden_7             CACCATGGCA CCCTAACCTA CGAAATTATC GACAACTTTA AAACATATGG -CTTTGATGT
Valence_4          CACCATGGCA CCCTAACCTA CGAAATTATC GACAACTTTA AAACATATGG -CTTTGATGT
Valence_2          CACCATGGCA CCCTAACCTA CGAAATTATC GACAACTTTA AAACATATGG -CTTTGATGT
Valence_1          CACCATGGCA CCCTAACCTA CGAAATTATC GACAACTTTA AAACATATGG -CTTTGATGT
Valence_7          CACCATGGCA CCCTAACCTA CGAAATTATC GACAACTTTA AAACATATGG -CTTTGATGT
Makindu_cM1        CACCATGGCA CCCTAACCTA CGAAATTATC GACAACTTTA AAACATATGG -CTTTGATGT
Makindu_cM6        CACCATGGCA CCCTAACCTA CGAAATTATC GACAACTTTA AAACATATGG -CTTTGATGT
Makindu_1          CACCATGGCA CCCTAACCTA CGAAATTATC GACAACTTTA AAACATATGG -CTTTGATGT
Amieu_5            CACCATAGCA CCCTAAACTA CGAAATTATC GACAACTTTA AAACATATGG -CTTTGATGT
Eden_11            CACCATAGCA CCCTAACCTA CGAAATTATC GACAACTTTA AAACATATGG -CTTTGATGT
Cann-River_10      CCCCAT---A CCC------A C-AAATTATC GACAACTTGA AAACATATGG -CTTTGATGT
Brazzaville_10     CCCCAT---A CCC------A C-AAATTATC GACAACTTGA AAACATATGG -CTTTGATGT
Cann-River_3       CACCATAGCA CCCTAACCTA CGAAATTATC GACAACTTTA AAACATATGG -CTTTGATGT
Cann-River_13      CACCATAGCA CCCTAACCTA CGAAATTATC GACAACTTTA AAACATATGG -CTTTGATGT
Eden_4             CACCATAGCA CCCTAACCTA CGAAATTATC GACAACTTTA AAACATATGG -CTTTGATGT
Makindu_10         CACCATAGCA CCCTAACCTA CGAAATTATC GACAACTTTA AAACATATGG -CTTTGATGT
Makindu_11         CACCATAGCA CCCTAACCTA CGAAATTATC GACAACTTTA AAACATATGG -CTTTGATGT
Brazzaville_11     CACCATGGCA CCCTAACCTA CGAAATTATC AACAACTTTA AAACATATGG -CTTTGATGT
Brazzaville_12     CACCATGGCA CCCTAACCTA CGAAATTATC GACAACTTTA AAACATATGG -CTTTGATGT
Brazzaville_8      CACCATGGCA CCCTAACCTA CGAAATTATC GACAACTTTA AAACATATGG -CTTTGATGT
chrU_963982        AACCATAGCA CCCTAACCTA CGAAATTATC GACAACTTTA AAA------- ----------
chr3L_18642496     ---------- ---------- ---------- ---------- ---------- ----------
chrU_3975907       ---------- ---------- ---------- ---------- ---------- ----------
chrU_10625041      ---------- ---------- ---------- ---------- ---------- ----------
chrU_2316095       ---------- ---------- ---------- ---------- ---------- ----------
chrU_4537377       CACCATAGCA CCGTAACCTA CGAAATTATC GACAACTTTA AAACATATGG -CTTTGATGT
chrU_5384045       -----TAGCA CCCTAACCTA CGAAATTATC GACAACTTTA AAACATATGG GCTTTGATAT
chrU_5680622       ---------- ---------- ---------- ---------- ---------- ----------
chrU_5950518       ---------- ---------- ---------- ---------- ---------- ----------
chrU_6132643       ---------- ---------- ---------- ---------- ---------- ----------
chrX_16602314      ---------- ---------- ---AATTATC GACAACTTTA AAACATATGG -CTTTGATGT
chrU_6536133       ---------- ---------- ---------- ---------- ---------- ----------
```

```
                901
chr3R_1506433    T-CTACAAGT ACACAACCCA AGATCCAGGA GAAATAGAGA AGAAAAACTT AATATATTCT
Brazzaville_16   T-CTACAAGT ACGTAACCCA AGATCCAGGG GAGATAGAGA AGAAAAACTT TATATATTCT
Eden_3           T-CTACAAGT ACATAACCCA AGATCCAGGA GAAATAGAGA AGAAAAACTT AATATATTCT
Cann-River_9     T-CTACAAGT ACATAACCCA AGATCCAGGA GAAATAGAGA AGAAAAACTT AATATATTCT
Amieu_2          T-CTACAAGT ACACAACCCA AGATCCAGGA GAAATAGAGA AGAAAAACTT AATATATTCT
Makindu_2        T-CTACAAGT ACATAACCCA AGATCCAGGG GAGATAGAGA AGAAAAACTT TATATATTCT
Makindu_6        T-CTACAAGT ACATAACCCA AGATCCAGGG GAGATAGAGA AGAAAAACTT TATATATTCT
Makindu_7        T-CTACAAGT ACATAACCCA AGATCCAGGG GAGATAGAGA AGAAAAACTT TATATATTCT
Valence_6        T-CTACAAGT ACATAACCCA AGATCCAGGG GAGATAGAGA AGAAAAACTT TATATATTCT
Makindu_8        T-CTACAAGT ACATAACCCA AGATCCAGGG GAGATAGAGA AGAAAAACTT TATATATTCT
Makindu_22       T-CTACAAGT ACATAACCCA AGATCCAGGG GAGATAGAGA AGAAAAACTT TATATATTCT
Canberra_5       T-CTACAAGT ACATAACCCA AGATCCAGGG GAGATAGAGA AGAAAAACTT TATATATTCT
Cann-River_2     T-CTACAAGT ACATAACCCA AGATCCAGGG GAGATAGAGA AGAAAGACTT TATATATTCT
Cann-River_6     T-CTACAAGT ACATAACCCA AGATCCAGGG GAGATAGAGA AGAAAAACTT TATATATTCT
Cann-River_7     T-CTACAAGT ACATAACCCA AGATCCAGGG GAGATAGAGA AGAAAAACTT TATATATTCT
Cann_River_5     T-CTACAAGT ACATAACCCA AGATCCAGGG GAGATAGAGA AGAAAAACTT TATATATTCT
Cann-River_8     TTCTACAAGT ACACAACCCA AGATCCAGGA GAAATAGAGA AGAAAAACTT AATATATTCT
Valence_5        T-CTACAAGT ACATAACCCA AGATCCAGGG GAGATAGAGA AGAAAAACTT TATATATTCT
Valence_9        T-CTACAAGT ACATAACCCA AGATCCAGGG GAGATAGAGA AGAAAAACTT TATATATTCT
Eden_1           T-CTACAAGT ACATAACCCA AGATCCAGGG GAGATAGAGA AGAAAAACTT TATATATTCT
Valence_13       T-CTACAAGT ACATAACCCA AGATCCAGGG GAGATAGAGA AGAAAAACTT TATATATTCT
Eden_7           T-CTACAAGT ACATAACCCA AGATCCAGGG GAGATAGAGA AGAAAAACTT TATATATTCT
Valence_4        T-CTACAAGT ACATAACCCA AGATCCAGGG GAGATAGAGA AGAAAAACTT TATATATTCT
Valence_2        T-CTACAAGT ACATAACCCA AGATCCAGGG GAGATAGAGA AGAAAAACTT TATATATTCT
Valence_1        T-CTACAAGT ACATAACCCA AGATCCAGGG GAGATAGAGA AGAAAAACTT TATATATTCT
Valence_7        T-CTACAAGT ACATAACCCA AGATCCAGGG GAGATAGAGA AGAAAAACTT TATATATTCT
Makindu_cM1      T-CTACAAGT ACATAACCCA AGATCCAGGG GAGATAGAGA AGAAAAACTT TATATATTCT
Makindu_cM6      T-CTACAAGT ACATAACCCA AGATCCAGGG GAGATAGAGA AGAAAAACTT TATATATTCT
Makindu_1        T-CTACAAGT ACATAACCCA AGATCCAGGG GAGATAGAGA AGAAAAACTT TATATATTCT
Amieu_5          T-CTACAAGT ACACAACCCA AGATCCAGGA GAAATAGAGA AGAAAAACTT AATATATTCT
Eden_11          T-CTACAAGT ACATAACCCA AGATCCAGGA GAAATAGAGA AGAAAAACTT AATATATTCT
Cann-River_10    T-CTACAAGT ACATAACCCA AGATCCAGGA CAAATAGAGA AGAAAAACTT AATATATTCT
Brazzaville_10   T-CTACAAGT ACATAACCCA AGATCCAGGA CAAATAGAGA AGAAAAACTT AATATATTCT
Cann-River_3     T-CTACAAGT ACACAACCCA AGATCCAGGA GAAATAGAGA AGAAAAACTT AATATGTTCT
Cann-River_13    T-CTACAAGT ACATAACCCA AGATCC-GGA GAAATAGAGA AGAAAAACTT AATATATTCT
Eden_4           T-CTACAAGT ACATAACCCA AGATCCAGGA GAAATAGAGA AGAAAAACTT AATATATTCT
Makindu_10       T-CTACAAGT ACATAACCCA AGATCCAGGA GAAATAGAGA AGAAAAACTT AATATATTCT
Makindu_11       T-CTACAAGT ACATAACCCA AGATCCAGGA GAAATAGAGA AGAAAAACTT AATATATTCT
Brazzaville_11   T-CTACAAGT ACATAACCCA AGATCCAGGG GAGATAGAGA AGAAAAACTT TATATATTCT
Brazzaville_12   T-CTACAAGT ACATAACCCA AGATCCAGGG GAGATAGAGA AGAAAAACTT TATATATTCT
Brazzaville_8    T-CTACAAGT ACATAACCCA AGATCCAGGG GAGATAGAGA AGAAAAACTT TATATATTCT
chrU_963982      ---------- ---------- ---------- ---------- ---------- ----------
chr3L_18642496   ---------- ---------- ---------- ---------- ---------- ----------
chrU_3975907     ---------- ---------- ---------- ---------- ---------- ----------
chrU_10625041    ---------- ---------- ---------- ---------- ---------- ----------
chrU_2316095     ---------- ---------- ---------- ---------- ---------- ----------
chrU_4537377     T-CTACAAGT ACACAACCCA AGATCCAGGA GAAATAGAGA AGAAAAACTT AATATATTCT
chrU_5384045     T-CTACAAGT ACATAACCCA AGATCCAGGA GAAATAGAGA AGAAAAACTT AATATATTCT
chrU_5680622     ---------- ---------- ---------- ---------- ---------- ----------
chrU_5950518     ---------- ---------- ---------- ---------- ---------- ----------
chrU_6132643     ---------- ---------- ---------- ---------- ---------- ----------
chrX_16602314    T-CTACAAGT ACATAACCAA AGATCCAGGA GAAATAGACA AGAAAAACTT AATATATTCT
chrU_6536133     ---------- ---------- ---------- ---------- ----AAACGT AC-ATATTCT
```

```
                  961
chr3R_1506433     TCATTAATAT AAAGACCTGT GCAAAAATTA ATGACATATA CGATATTAAC ACAATATGCC
Brazzaville_16    TCATTAATAT AAAGACCTGT GCAAAAATTA ATGACATATA CGATATTAAC ACAATATGCC
Eden_3            TCATTA-TAT AAAGACCTAT GCAAAAATTA ATGACATATA CGATATTAAC ACAATATGCC
Cann-River_9      TCATTAATAT AAAGACCTAT GCAAAAATTA ATGACATATA CGATATTAAC ACAATATGCC
Amieu_2           TCATTAATAT AAAGACCTGT GCAAAAATTA ATGACATATA CGATATTAAC ACAATATGCC
Makindu_2         TCATTAATAT AAAGACCTGT GCAAAAATTA ATGACATATA CGATATTAAC ACAATATGCC
Makindu_6         TCATTAATAT AAAGACCTGT GCAAAAATTA ATGACGTATA CGATATTAAC ACAATATGCC
Makindu_7         TCATTAATAT AAAGACCTGT GCAAAAATTA ATGACATATA CGATATTAAC ACAATATGCC
Valence_6         TCATTAATAT AAAGACCTGT GCAAAAATTA ATGACATATA CGATATTAAC ACAATATGCC
Makindu_8         TCATTAATAT AAAGACCTGT GCAAAAATTA ATGACATATA CGATATTAAC ACAATATGGC
Makindu_22        TCATTAATAT AAAGACCTGT GCAAAAATTA ATGACATATA CGATATTAAC ACAATATGCC
Canberra_5        TCATTAATAT AAAGACCTGT GCAAAAATTA ATGACATATA CGATATTAAC ACAATATGCC
Cann-River_2      TCATTAATAT TAAGACCTGT GCAAAAATTA ATGACATATA CGATATTAAC ACAATATGCC
Cann-River_6      TCATTAATAT AAAGACCTGT GCAAAAATTA ATGACATATA CGATATTAAC ACAATATGCC
Cann-River_7      TCAT-AATAT AAAGACCTGT GCAAAAATTA ATGACATATA CGATATTAAC ACAATATGCC
Cann_River_5      TCGTTAATAT AAAGACCTGT GCAAAAATTA ATGACATATA CGATATTAAC ACAATATGCC
Cann-River_8      TCATTAATAT AAAGACCTGT GCAAAAATTA ATGACATATA CGATATTAAC ACAATATGCC
Valence_5         TCATTAATAT AAAGACCTGT GCAAAAATTA ATGACATATA CGATATTAAC ACAATATGCC
Valence_9         TCATTAATAT AAAGACCTGT GCAAAAATTA ATGACATATA CGATATTAAC ACAATATGCC
Eden_1            TCATTAATAT AAAGACCTGT GCAAAAATTA ATGACATATA CGATATTAAC ACAATATGCC
Valence_13        TCATTAATAT AAAGACCTGT GCAAAAATTA ATGACATATA CGATATTAAC ACAATATGCC
Eden_7            TCATTAATAT AAAGACCTGT GCAAAAATTA ATGACATATA CGATATTAAC ACAATATGCC
Valence_4         TCATTAATAT AAAGACCTGT GCAAAAATTA ATGACATATA CGATATTAAC ACAATATGCC
Valence_2         TCATTAATAT AAAGACCTGT GCAAAAATTA ATGACATATA CGATATTAAC ACAATATGCC
Valence_1         TCATTAATAT AAAGACCTGT GCAAAAATTA ATGACATATA CGATATTAAC ACAATATGCC
Valence_7         TCATTAATAT AA-GACCTGT GCAAAAATTA ATGACATATA CGATATTAAC ACAATATGCC
Makindu_cM1       TCATTAATAT AAAGACCTGT GCAAAAATTA ATGACATATA CGATATTAAC ACAATATGCC
Makindu_cM6       TCATTAATAT AAAGACCTGT GCAAAAATTA ATGACATATA CGATATTAAC ACAATATGCC
Makindu_1         TCATTAATAT AAAGACCTGT GCAAAAATTA ATGACATATA CGATATTAAC ACAATATGCC
Amieu_5           TCATTAATAT AAAGACCTGT GCAAAAATTA ATGACATATA CGATATTAAC ACAATATGCC
Eden_11           TCA------- ---------- ---AAAATTA ATGACATA-- CGATATTAAC ACAAT-----
Cann-River_10     TCATTAATAT AAAGACCTGT GTAAAAATTA ATGACATATA CGATATTAAC ACAATATGCC
Brazzaville_10    TCATTAATAT AAAGACCTGT GTAAAAATTA ATGACATATA CGATATTAAC ACAATATGCC
Cann-River_3      TCATTAATAT AAAGACCTAT GCAAAAATTA ATGACATATA CGATATTAAC ACAATATGCC
Cann-River_13     TCA------- ---------- ---AAAATTA ATGACATA-- CGATATTAAC ACAAT-----
Eden_4            TCA------- ---------- ---AAAATTA ATGACATA-- CGATATTAAC ACAAT-----
Makindu_10        TCA------- ---------- ---AAAATTA ATGACATA-- CGATATTAAC ACAAT-----
Makindu_11        TCA------- ---------- ---AAAATTA ATGACATA-- CGATATTAAC ACAAT-----
Brazzaville_11    TCATTAATAT AAAGACCTGT GCAAAAATTA ATGACATATA CGATATTAAC ACAATATGCC
Brazzaville_12    TCATTAATAT AAAGACCTGT GCAAAAATTA ATGACATATA CGATATTAAC ACAATATGCC
Brazzaville_8     TCATTAATAT AAAGACCTGT GCAAAAATTA ATGACATATA CGATATTAAC ACAATATGCC
chrU_963982       ---------- ---------- ---------- ---------- ---------- ----------
chr3L_18642496    ---------- ---------- ---------- ---------- ---------- ----------
chrU_3975907      ---------- ---------- ---------- ---------- ---------- ----------
chrU_10625041     ---------- ---------- ---------- ---------- ---------- ----------
chrU_2316095      ---------- ---------- ---------- ---------- ---------- ----------
chrU_4537377      TCATTAATAT AAAGACCTGT GCAAAAATTA ATGACATATA CGATATTAAC ACAATATGCC
chrU_5384045      TCATTAATAT AAAGACCTAT GCAAAAATTA ATGACATATA CGATATTAAC ACAATATGCC
chrU_5680622      ---------- ---------- ---------- ---------- ---------- ----------
chrU_5950518      ---------- ---------- ---------- ---------- ---------- ----------
chrU_6132643      ---------- ---------- ---------- ---------- ---------- ----------
chrX_16602314     TCATTAATAT AAAGTCCTGT GCAAAAATTA ATGACGTATG CGATATTAAC ACAATATGCC
chrU_6536133      TCATTAATAT AAAGTCATAT GCAAATATAA ATGACATA-- CGATATTAAC ACACTATGCC
```

```
                1021
chr3R_1506433     GACAGAAAGT GCGGATAGAA AGAATGCGTA AATCATCTGA AATTGCACAA TGCATACGTT
Brazzaville_16    GACAGAAAGT GCGGATAGAA AGAATGCGTA AATCATCTAA AATTGCTCAA TGCATACGTT
Eden_3            GACAGAAAGT GCGGATAGAA AGAATGCGTT AATCATCTGA AATAGCTCAA TGCATACGTT
Cann-River_9      GACAGAAAGT GCGGATAGAA AGAATGCGTT AATCATCTGA AATAGCTCAA TGCATACGTT
Amieu_2           GACAGAAAGT GCGGATAGAA AGAATGCGTA AATCATCTGA AATTGCACAA TGCATACGTT
Makindu_2         GACAGAAAGT GCGGATAGAA AGAATGCGTA AATCATCTAA AATTGCTCAA TGCATACGTT
Makindu_6         GACAGAAAGT GCGGATAGAA AGAATGCGTA AATCATCTAA AATTGCTCAA TGCATACGTT
Makindu_7         GACAGAAAGT GCGGATAGAA AGAATGCGTA AATCATCTAA AATTGCTCAA TGCATACGTT
Valence_6         GACAGAATGT GCGGATAGAA AGAATGCGTA AATCATCTAA AATTGCTCAA TGCATACGTT
Makindu_8         GACAGAAAGT GCGGATAGAA AGAATGCGTA AATCATCTAA AATTGCTCAA TGCATACGTT
Makindu_22        GACAGAAAGT GCGGATAGAA AGAATGCGTA AATCATCTAA AATTGCTCAA TGCATACGTT
Canberra_5        GACAGAAAGT GCGGATAGAA AGAATGCGTA AATCATCTAA AATTGCTCAA TGCATACGTT
Cann-River_2      GACAGAAAGT GCGGATAGAA AGAATGCGTA AATCATCTAA AATTGCTCAA TGCATACGTT
Cann-River_6      GACAGAAAGT GCGGATAGAA AGAATGCGTA AATCATCTAA AATTGCTCAA TGCATACGTT
Cann-River_7      GACAGAAGGT GCGGATAGAA AGAATGCGTA AATCATCTAA AATTGCTCAA TGCATACGTT
Cann_River_5      GACAGAAAGT GCGGATAGAA AGAATGCGTA AATCATCTAA AATTGCTCAA TGCATACGTT
Cann-River_8      GACAGAAAGT GCGGATAGAA AGAATGCGTA AGTCATCTAA AATTGCTCAA TGCATACGTT
Valence_5         GACAGAATGT GCGGATAGAA AGAATGCGTA AATCATCTAA AATTGCTCAA TGCATACGTT
Valence_9         GACAGAATGT GCGGATAGAA AGAATGCGTA AATCATCTAA AATTGCTCAA TGCATACGTT
Eden_1            GACAGAAAGT GCGGATAGAA AGAATGCGTA AATCATCTAA AATTGCTCTA TGCATACGTT
Valence_13        GACAGAATGT GCGGATAGAA AGAATGCGTA AATCATCTAA AATTGCTCAA TGCATACGTT
Eden_7            GACAGAAAGT GCGGATAGAA AGAATGCGTA AATCATCTAA AATTGCTCTA TGCATACGTT
Valence_4         GACAGAATGT GCGGATAGAA AGAATGCGTA AATCATCTAA AATTGCTCAA TGCATACGTT
Valence_2         GACAGAATGT GCGGATAGAA AGAATGCGTA AATCATCTAA AATTGCTCAA TGCATACGTT
Valence_1         GACAGAATGT GCGGATAGAA AGAATGCGTA AATCATCTAA AATTGCTCAA TGC-------
Valence_7         GACAGAATGT GCGGATAGAA AGAATGCGTA AATCATCTAA AATTGCTCAA TGCATACGTT
Makindu_cM1       GACAGAATGT GCGGATAGAA AGAATGCGTA AATCATCTAA AATTGCTCAA TGCATACGTT
Makindu_cM6       GACAGAATGT GCGGATAGAA AGAATGCGTA AATCATCTAA AATTGCTCAA TGCATACGTT
Makindu_1         GACAGAAAGT GCGGATAGAA AGAATGCGTA AATCATCTAA AATTGCTCAA TGCATACGTT
Amieu_5           GACAGAAAGT GCGGATAGAA AGAATGCGTA AATCATCTGA AATTGCACAA TGCATACGTT
Eden_11           ---------- ---------- ---------- ---CATCTGA AATTGCTCAA TGCATACGTT
Cann-River_10     GACAGAAAGT ACGGATAGAA AGAATGCATA AATCATCTGA AATTTCTCAA TGCATACGTT
Brazzaville_10    GACAGAAAGT ACGGATAGAA AGAATGCATA AATCATCTGA AATTTCTCAA TGCATACGTT
Cann-River_3      GACAGAAAGT GCGGATAGAA AGAATGCGTA AATCATCTGA AATTGCGCAA TGCATACGTT
Cann-River_13     ---------- ---------- ---------- ---CATCTGA AATTGCTCAA TGCATACGTT
Eden_4            ---------- ---------- ---------- ---CATCTGA AATTGCTCAA TGCATACGTT
Makindu_10        ---------- ---------- ---------- ---CATCTGA AATTGCTCAA TGCATACGTT
Makindu_11        ---------- ---------- ---------- ---CATCTGA AATTGCTCAA TGCATACGTT
Brazzaville_11    GACAGAAAGT GCGGATAGAA AGAATGCGTA AATCATCTAA AATTGCTCAA TGCATACGTT
Brazzaville_12    GACAGAAAGT GCGGATAGAA AGAATGCGTA AATCATCTAA AATTGCTCAA TGCATACGTT
Brazzaville_8     GACAGAAAGT GCGGATAGAA AGAATGCGTA AATCATCTAA AATTGCTCAA TGCATACGTT
chrU_963982       ---------- ---------- ---------- ---------- ---------- ----------
chr3L_18642496    ---------- ---------- ---------- ---------- ---------- ----------
chrU_3975907      ---------- ---------- ---------- ---------- ---------- ----------
chrU_10625041     ---------- ---------- ---------- ---------- ---------- ----------
chrU_2316095      ---------- ---------- ---------- ---------- ---------- ----------
chrU_4537377      GACAGAAAGT GCGGATAGAA AGAATGCGTA AATCATCTGA AATTGCACAA TGCATACGTT
chrU_5384045      GACAGAAAGT GCGGATAGAA AGAATGCGTT AATCATCTGA AATAGCTCAA TGCATACGTT
chrU_5680622      ---------- ---------- ---------- ---------- ---------- ----------
chrU_5950518      ---------- ---------- ---------- ---------- ---------- ----------
chrU_6132643      ---------- ---------- ---------- ---------- ---------- ----------
chrX_16602314     GACAGAAAGT GCGGATAGAA AGAATGCGTA AATCATCTGA AATTGCTCAA TGCATACGTT
chrU_6536133      GACAGAAAAT GCGGATGGAA AAAATGCGTA AGTCTTCGGA AATTGCTCAA TGCATACGTT
```

```
                    1081
chr3R_1506433       GTCAGGAATT CGGCCACACA GCCTCGATGT GGTGAAAATC ACTTAACCAA GCTATGCGTA
Brazzaville_16      GTCAGGAATT CGGCCACACA GCCACGATGT GGTGAAAATC ACTTAACCAA GCTATGCGTA
Eden_3              GTCAGGAATT CGGCCACAAA GCCTCGATGT GGTGAAAATC ACTCAACCAA GCTATGCGTA
Cann-River_9        GTCAGGAATT CGGCCACAAA GCCTCGATGT GGTGAAAATC ACTTAACCAA GCTATGCGTA
Amieu_2             GTCAGGAATT CGGCCACACA GCCTCGATGT GGTGAAAATC ACTTAACCAA GCTATGCGTA
Makindu_2           GTCAGGAATT CGGCCACACA GCCACGATGT GGTGAAAATC ACTTAACCAA GCTATGCGTA
Makindu_6           GTCAGGAATT CGGCCACACA GCCACGATGT GGTGAAAATC ACTTAACCAA GCTATGCGTA
Makindu_7           GTCAGGAATT CGGCCACACA GCCACGGTGT GGTGAAAATC ACTTAACCAA GCTATGCGTA
Valence_6           GTCAGGAATT CGGCCACACA GCCACGATGT GGTGAAAATC ACTTAACCAA GCTATGCGTA
Makindu_8           GTCAGGAATT CGGCCACACA GCCACGATGT GGTGAAAATC ACTTAACCAA GCTATGCGTA
Makindu_22          GTCAGGAATT CGGCCACACA GCCACGATGT GGTGAAAATC ACTTAACCAA GCTATGCGTA
Canberra_5          GTCAGGAATT CGGCCACACA GCCACGATGT GGTGAAAATC ACTTAACCAA GCTATGCGTA
Cann-River_2        GTCAGGAATT CGGCCACACA GCCACGATGT GGTGAAAATC ACTTAACCAA GCTATGCGTA
Cann-River_6        GTCAGGAATT CGGCCACACA GCCACGATGT GGTGAAAATC ACTTAACCAA GCTATGCGTA
Cann-River_7        GTCAGGAATT CGGCCACACA GCCACGATGT GGTGAAAATC ACTTAACCAA GCTATGCGTA
Cann_River_5        GTCAGGAATT CGGCCACACA GCCACGATGT GGTGAAAATC ACTTAACCAA GCTATGCGTA
Cann-River_8        GTCAGGAATT CGGCCACACA GCCACGATGT GGTGAAAATC ACTTAACCAA GCTATGCGTA
Valence_5           GTCAGGAATT CGGCCACACA GCCACGATGT GGTGAAAATC ACTTAACCAA GCTATGCGTA
Valence_9           GTCAGGAATT CGGCCACACA GCCACGATGT GGTGAAAATC ACTTAACCAA GCTATGCGTA
Eden_1              GTCAGGAATT CGGCCACACA GCCACGATGT GGTGAAAATC ACTTAACCCA GCTATGCGTA
Valence_13          GTCAGGAATT CGGCCACACA GCCACGATGT GGTGAAAATC ACTTAACCAA GCTATGCGTA
Eden_7              GTCAGGAATT CGGCCACACA GCCACGATGT GGTGAAAATC ACTTAACCAA GCTATGCGTA
Valence_4           GTCAGGAATT CGGCCACACA GCCACGATGT GGTGAAAATC ACTTAACCAA GCTATGCGTA
Valence_2           GTCAGGAATT CGGCCACACA GCCACGATGT GGTGAAAATC ACTTAACCAA GCTATGCGTA
Valence_1           ---------- ---------- ---------- ---------- ---------- ----------
Valence_7           GTCAGGAATT CGGCCACACA GCCACGATGT GGTGAAAATC ACTTAACC-- ----------
Makindu_cM1         GTCAGGAATT CGGCCACACA GCCACGATGT GGTGAAAATC ACTTAACCAA GCTATGCGTA
Makindu_cM6         GTCAGGAATT CGGCCACACA GCCACGATGT GGTGAAAATC ACTTAACCAA GCTATGCGTA
Makindu_1           GTCAGGAATT CGGCCACACA GCCACGATGT GGTGAAAATC ACTTAACCAA GCTATGCGTA
Amieu_5             GTCAGGAATT CGGCCACACA GCCTCGATGT GGTGAAAATC ACTTAACCAA GCTATGCGTA
Eden_11             GTCAGGAATT CGGCCACACA GCCTCGATGT GGTGAAAATC ACTTAACCAA GCTATGCGTA
Cann-River_10       GTCAGGAATT CGTCCACACA GCCTCGATGT GGTGAAAATC ACTTAACCAA GCTATGCGTA
Brazzaville_10      GTCAGGAATT CGTCCACACA GCCTCGATGT GGTGAAAATC ACTTAACCCA GCTATGCGTA
Cann-River_3        GTCAGGAATT CGGCCACACA GCCTCGATGT GGTGAAAATC ACTTAACCAA GCTATGCGTA
Cann-River_13       GTCAGGAATT CGGCCACACA GCCTCGATGT GGTGAAAATC ACTTAACCAA GCTATGCGTA
Eden_4              GTCAGGAATT CGGCCACACA GCCTCGATGT GGTGAAAATC ACTTAACCAA GCTATGCGTA
Makindu_10          GTCAGGAATT CGGCCACACA GCCTCGATGT GGTGAAAATC ACTTAACCAA GCTATGCGTA
Makindu_11          GTCAGGAATT CGGCCACACA GCCTCGATGT GGTGAAAATC ACTTAACCAA GCTATGCGTA
Brazzaville_11      GTCAGGAATT CGGCCACACA GCCACGATGT GGTGAAAATC ACTTAACCAA GCTATGCGTA
Brazzaville_12      GTCAGGAATT CGGCCACACA GCCACGATGT GGTGAAAATC ACTTAACCAA GCTATGCGTA
Brazzaville_8       GTCAGGAATT CGGCCACACA GCCACGATGT GGTGAAAATC ACTTAACCAA GCTATGCGTA
chrU_963982         ---------- ---------- ---------- ---------- ---------- ----------
chr3L_18642496      ---------- ---------- ---------- ---------- ---------- ----------
chrU_3975907        ---------- ---------- ---------- -----AAATC ACTTAACCA- GCTATGCGTA
chrU_10625041       ---------- ---------- ---------- ---------- ---------- ----------
chrU_2316095        ---------- ---------- --CTCGATGT GGTGAAAATC ACTTAACCAA GCTATGCGTA
chrU_4537377        GTCAGGAATT TGGCCACACA GCCTCGATGT GGTGAAAATC ACTTAACCAA GCTATGCGTA
chrU_5384045        GTCAGGAATT CGGCCACAAA GCCTCGATGT GGTGAAAATC ACTTAACCAA GCTATGCGTA
chrU_5680622        ---------- ---------- ---------- ---------- ---------- ----------
chrU_5950518        ---------- ---------- ---------- ---------- ---------- ----------
chrU_6132643        ---------- ---------- ---------- ---------- ---------- ----------
chrX_16602314       GTCAGGAATT CGGCCACACA GCCTCGATGT GGTGAAAATC ACTTAACCAA GCTATGCGTA
chrU_6536133        GTCAGGAATT CGGACATACA GCCTCGCTGT GGCGAAAATC ACTTAACCAA GTTATGCGGG
```

```
                  1141
chr3R_1506433     CTTCCCAATG ATCAACAGCC -------TAT CTGTATACAC TGTGGAGGAA ATCACACGGC
Brazzaville_16    CTTCCCAATG ATCAACAGCC -------TAT CTGTATACAC TGTGGAGGAA ATCACAAGGC
Eden_3            CTTCCCAATG ATCAGCAGCC -------TAT CTGTATACAC TGTGGAGGAA ATCACACGGC
Cann-River_9      CTTCCCAATG ATCAACAGCC -------TAT CTGTATACAC TGTGGAGGAA ATCACACGGC
Amieu_2           CTTCCCAATG ATCAACAGCC -------TAT CTGTATACAC TGTGGAGGAA ATCACACGGC
Makindu_2         CTTCCCAATG ATCAACAGCC -------TAT CTGTATACAC TGTGGAGGAA ATCACAAGGC
Makindu_6         CTTCCCAATG ATCAACCGCC -------TAT CTGTATACAC TGTGGAGGAA ATCACAAGGC
Makindu_7         CTTCCCAATG ATCAACAGCC -------TAT CTGTATACAC TGTGGAGGAA ATCACAAGGC
Valence_6         CTTCCCAATG ATCAACAGCC -------TAT CTGTATACAC TGTGGAGGAA ATCACAAGGC
Makindu_8         CTTCCCAATG ATCAACAGCC -------TAT CTGTATACAC TGTGGAGGAA ATCACAAGGC
Makindu_22        CTTCCCAATG ATCAACAGCC -------TAT CTGTATACAC TGTGGAGGAA ATCACAAGGC
Canberra_5        CTTCCCAATG ATCAACAGCC -------TAT CTGTATACAC TGTGGAGGAA ATCACAAGGC
Cann-River_2      CTTCCCAATG ATCAACAGCC -------TAT CTGTATACAC TGTGGAGGAA ATCACAAGGC
Cann-River_6      CTTCCCAATG ATCAACAGCC -------TAT CTGTATACAC TGTGGAGGAA ATCACAAGGC
Cann-River_7      CTTCCCAATG ATCAACAGCC -------TAT CTGTATACAC TGTGGAGGAA ATCACAAGGC
Cann_River_5      CTTCCCAATG ATCAACAGCC -------TAT CTGTATACAC TGTGGAGGAA ATCACAAGGC
Cann-River_8      CTTCCCAATG ATCAACAGCC -------TAT CTGTATACAC TGTGGAGGAA ATCACAAGGC
Valence_5         CTTCCCAATG ATCAACAGCC -------TAT CTGTATACAC TGTGGAGGAA ATCACAAGGC
Valence_9         CTTCCCAATG ATCAACAGCC -------TAT CTGTATACAC TGTGGAGGAA ATCACAAGGC
Eden_1            CTTCCCAATG ATCAACAGCC -------TAT CTGTATACAC TGTGGAGGAA ATCACAAGGC
Valence_13        CTTCCCAATG ATCAACAGCC -------TAT CTGTATACAC TGTGGAGGAA ATCACAAGGC
Eden_7            CTTCCCAATG ATCAACAGCC -------TAT CTGTATACAC TGTGGAGGAA ATCACAAGGC
Valence_4         CTTCCCAATG ATCAACAGCC -------TAT CTGTATACAC TGTGGAGGAA ATCACAAGGC
Valence_2         CTTCCCAATG ATCAACAGCC -------TAT CTGTATACAC TGTGGAGGAA ATCACAAGGC
Valence_1         ---------- ---------- ---------- ---------- ---------- ----------
Valence_7         ---------- ---------- ---------- ---------- ---------- ----------
Makindu_cM1       CTTCCCAATG ATCAACAGCC -------TAT CTGTATACAC TGTGGAGGAA ATCACAAGGC
Makindu_cM6       CTTCCCAATG ATCAACAGCC -------TAT CTGTATACAC TGTGGAGGAA ATCACAAGGC
Makindu_1         CTTCCCAATG ATCAACAGCC -------TAT CTGTATACAC TGTGGAGGAA ATCACAAGGC
Amieu_5           CTTCCCAATG ATCAACAGCC -------TAT CTGTATACAC TGTGGAGGAA ATCACACGGC
Eden_11           CTTCCCAATG ATCAACAGCC -------TAT CTGTATACAC TGTGGAGGAA ATCACACAGC
Cann-River_10     CTTCCCAATG ATCAACAGCC -------TAT CTGTATACAC TGTGGAGGAA ATCACACGGC
Brazzaville_10    CTTCCCAATG ATCAACAGCC -------TAT CTGTATACAC TGTGGAGGAA ATCACACGGC
Cann-River_3      CTTCCCAATG ATCAACAGCC -------TAT CTGTATACAC TGTGGAGGAA ATCACACGGC
Cann-River_13     CTTCCCAATG ATCAACAGCC -------TAT CTGTATACAC TGTGGAGGAA ATCACACAGC
Eden_4            CTTCCCAATG ATCAACAGCC -------TAT CTGTATACAC TGTGGAGGAA ATCACACAGC
Makindu_10        CTTCCCAATG ATCAACAGCC -------TAT CTGTATACAC TGTGGAGGAA ATCACACAGC
Makindu_11        CTTCCCAATG ATCAACAGCC -------TAT CTGTATACAC TGTGGAGGAA ATCACACAGC
Brazzaville_11    CTTCCCAATG ATCAACAGCC -------TAT CTGTATACAC TGTGGAGGAA ATCACAAGGC
Brazzaville_12    CTTCCCAATG ATCAACAGCC -------TAT CTGTATACAC TGTGGAGGAA ATCACAAGGC
Brazzaville_8     CTTCCCAATG ATCAACAGCC -------TAT CTGTATACAC TGTGGAGGAA ATCACAAGGC
chrU_963982       ---------- ---------- ---------- ---------- ---------- ----------
chr3L_18642496    ---------- ---------- ---------- ---------- ---------- ----------
chrU_3975907      CT-CCC-ATG ATCA-CAGCC -------TAT CTGTATACAC TGTGGAGGAA -TCACACGGC
chrU_10625041     ---------- ---------- ---------- ---------- ---------- --CACACTGC
chrU_2316095      CTTCCCAATG ATCAACAGCC -------TAT CTGTATACAC TGTGGAGGAA ATCACACGGC
chrU_4537377      CTTCCCAATG ATCAACAGCC -------TAT CTGTATACAC TGTGGAGGAA ATCACACGGC
chrU_5384045      CTTCCCAATG ATCAACAGCC -------TAT CTGTATACAC TGTGGAGGAA ATCACACGGC
chrU_5680622      ---------- ---------- ---------- ---------- ---------- ----------
chrU_5950518      ---------- ---------- ---------- ---------- ---------- ----------
chrU_6132643      ---------- ---------- ---------- ---------- ---------- ----------
chrX_16602314     CTTCCCAATG ATCAACAGCC -------TAT CTGTATACAC TGTGGAGGAA ATCACACGGC
chrU_6536133      CTTCCCAATG ATCA---GCC AACAGCCTAT CAGT------ ---GGAGGAA ATCATTCGGC
```

```
                1201
chr3R_1506433     AAGTTACAAG GGTTGCCAGT TTTACCAGGA GTATCTTCGA CGATCAATGG GCACTGTAAA
Brazzaville_16    AAGCTACAAA GGTTGCCAGT TTTACCAGGA GTATCTTCGA CGATCAATGG GCACTGTAAA
Eden_3            AAGCTACAAG GGTTGCCAGT TTTACCAGGA GTATCTTCGA CGATCACTG- --ACTGTAAA
Cann-River_9      AAGCTACAAA GGTTGCCAGT TTTACCAGGA GTATCTTCGA CGATCACTG- --ACTGTAAA
Amieu_2           AAGTTACAAG GGTTGCCAGT TTTACCAGGA GTATCTTCGA CGATCAATGG GCACTGTAAA
Makindu_2         AAGCTACAAA GGTTGCCAGT TTTACCAGGA GTATCTTCGA CGATCAATGG GCACTGTAAA
Makindu_6         AAGCTACAAA GGTTGCCAGT TTTACCAGGA GTATCTTCGA CGATCAATGG GCACTGTAAA
Makindu_7         AAGCTACAAA GGTTGCCAGT TTTACCAGGA GTATCTTCGA CGATCAATGG GCACTGTAAA
Valence_6         AAGCTACAAA GGTTGCCAGT TTTACCAGGA GTATCTTCGA CGATCAATGG GCACTGTAAA
Makindu_8         AAGCTACAAA GGTTGCCAGT TTTACCAGGA GTATCTTCGA CGATCAATGG GCACTGTAAA
Makindu_22        AAGCTACAAA GGTTGCCAGT TTTACCAGGA GTATCTTCGA CGATCAATGG GCACTGTAAA
Canberra_5        AAGCTACAAA GGTTGCCAGT TTTACCAGGA GTATCTTCGA CGATCAATGG GCACTGTAAA
Cann-River_2      AAGCTACAAA GGTTGCCAGT TTTACCAGGA GTATCTTCGA CGATCAATGG GCACTGTAAA
Cann-River_6      AAGCTACAAA GGTTGCCAGT TTTACCAGGA GTATCTTCGA CGATCAATGG GCACTGTAAA
Cann-River_7      AAGCTACAAA GGTTGCCAGT TTTACCAGGA GTATCTTCGA CGATCAATGG GCACTGTAAA
Cann_River_5      AAGCTACGAA GGTTGCCAGT TTTACCAGGA GTATCTTCGA CGATCAATGG GCACTGTAAA
Cann-River_8      AAGCTACAAA GGTTGCCGGT TTTACCAGGA GTATCTTCGA CGATCAATGG GCACTGTAAA
Valence_5         AAGCTACAAA GGTTGCCAGT TTTACCAGGA GTATCTTCGA CGATCAATGG GCACTGTAAA
Valence_9         AAGCTACAAA GGTTGCCAGT TTTACCAGGA GTATCTTCGA CGATCAATGG GCACTGTAAA
Eden_1            AAGCTACAAA GGTTGCCAGT TTTACCAGGA GTATCTTCGA CGATCAATGG GCACTGTAAA
Valence_13        AAGCTACAAA GGTTGCCAGT TTTACCAGGA GTATCTTCGA CGATCAATGG GCACTGTAAA
Eden_7            AAGCTACAAA GGTTGCCAGT TTTACCAGGA GTATCTTCGA CGATCAATGG GCACTGTAAA
Valence_4         AAGCTACAAA GGTTGCCAGT TTTACCAGGA GTATCTTCGA CGATCAATGG GCACTGTAAA
Valence_2         AAGCTACAAA GGTTGCCAGT TTTACCAGGA GTATCTTCGA CGATCAATGG GCACTGTAAA
Valence_1         ---------- ---------- ---------- ---------- ---------- ----------
Valence_7         ---------- ---------- ---------- ---------- ---------- ----------
Makindu_cM1       AAGCTACAAA GGTTGCCAGT TTTACCAGGA GTATCTTCGA CGATCAATGG GCACTGTAAA
Makindu_cM6       AAGCTACAAA GGTTGCCAGT TTTACCAGGA GTATCTTCGA CGATCAATGG GCACTGTAAA
Makindu_1         AAGCTACAAA GGTTGCCAGT TTTACCAGGA GTATCTTCGA CGATCAATGG GCACTGTAAA
Amieu_5           AAGTTACAAG GGTTGCCAGT TTTACCAGGA GTATCTTCGA CGATCAATGG GCACTGTAAA
Eden_11           AAGTTACAAG GGTTGCCAGT TTTACCAGGA GTATCTTCGA CGATCAATGG GCACTGTAAA
Cann-River_10     AAGTTACAAG GGTTGCCAGT TTTACCAGGA GTATCTTCGA CGATCAATGG GCACTGTAAA
Brazzaville_10    AAGTTACAAG GGTTGCCAGT TTTACCAGGA GTATCTTCGA CGATCAATGG GCACTGTAAA
Cann-River_3      AAGTTACAAG GGTTGCCAGT TTTACCAGGA GTATCTTCGA CGATCAATGG GCACTGTAAA
Cann-River_13     AAGTTACAAG GGTTGCCAGT TTTACCAGGA GTATCTTCGA CGATCAATGG GCACTGTAAA
Eden_4            AAGTTACAAG GGTTGCCAGT TTTACCAGGA GTATCTTCGA CGATCAATGG GCACTGTAAA
Makindu_10        AAGTTACAAG GGTTGCCAGT TTTACCAGGA GTATCTTCGA CGATCAATGG GCACTGTAAA
Makindu_11        AAGTTACAAG GGTTGCCAGT TTTACCAGGA GTATCTTCGA CGATCAATGG GCACTGTAAA
Brazzaville_11    AAGCTACAAA GGTTGCCAGT TTTACCAGGA GTATCTTCGA CGATCAATGG GCACTGTAAA
Brazzaville_12    AAGCTACAAA GGTTGCCAGT TTTACCAGGA GTATCTTCGA CGATCAATGG GCACTGTAAA
Brazzaville_8     AAGCTACAAA GGTTGCCAGT TTTACCAGGA GTATCTTCGA CGATCAATGG GCACTGTAAA
chrU_963982       ---------- ---------- ---------- ---------- ---------- ----------
chr3L_18642496    ---------- ---------- ---------- ---------- ---------- ----------
chrU_3975907      AAGTTACAAG GGTTGCCAGT TTTACCAGGA GTATCTTTGA CGATCAATGG GCACTGTAAA
chrU_10625041     AAGTTACAAA GGTTGCAAGT GGTACCAGGA GTATCTTCGA CGATCAATGG GCACTGTAAA
chrU_2316095      AAGTTACAAG GGTTGCCAGT TTTACCAGGA GTATCTTCGA CGATCAATGG GCACTGTAAA
chrU_4537377      AAGTTACAAG GGTTGCCAGT TTTACCAGGA GTATCTTCGA CGATCAATGG GCACTGTAAA
chrU_5384045      AAGCTACAAA GGTTGCCAGT TTTACCAGGA GTATCTTCGA CGATCACTG- --ACTGTAAA
chrU_5680622      ---------- ---------- ---------- ---------- ---------- ----------
chrU_5950518      ---------- ---------- -----CAGGA GTATCTTCGA CGATCAATAA GCACTGTAAA
chrU_6132643      ---------- ---------- ---------- ---------- ---------- ----------
chrX_16602314     AAGCTACAAA GGCTGCCAGT TTTACCAGGA GTATCTTCGA CGATCAATGG GCACTGTAAA
chrU_6536133      AAGTTACAAA CG-------- ---------- ----CTTACA -----AATGG GCACTATAAG
```

```
                  1261
chr3R_1506433     GAAGCAACAA CCTAAGCAAG CCAAGTTTGA TAAAAGTGCA ACAAACCAAC AACAACCCCA
Brazzaville_16    GAAGCAACAA CCTAAGCAAG CCAGGTTAGA TAAAAGTGCA ACCAACCAGC AACAACCCCA
Eden_3            GAAGCAACAA CCTAAGCAAG CCAGGTTAGA TAAAAGTGCA ACCAACCAGC AACAACCCCA
Cann-River_9      GAAGCAACAA CCTAAGCAAG CCAGGTTAGA TAAAAGTGCA ACCAACCAGC AACAACCCCA
Amieu_2           GAAGCAACAA CCTAAGCAAG CCAAGTTTGA TAAAAGTGCA ACAAACCAAC AACAACCCCA
Makindu_2         GAAGCAACAA CCTAAGCAAG CCAGGTTAGA TAAAAGTGCA ACCAACCAGC AACAACCCCA
Makindu_6         GAAGCAACAA CCTAAGCAAG CCAGGTTAGA TAAAAGTGCA ACCAACCAGC AACAACCCCA
Makindu_7         GAAGCAACAA CCTAAGCAAG CCAGGTTAGA TAAAAGTGCA ACCAACCAGC AACAACCCCA
Valence_6         GAAGCAACAA CCTAAGCAAG CCAGGTTAGA TAAAAGTGCA ACCAACCAGC AACAACCCCA
Makindu_8         GAAGCAACAA CCTAAGCAAG CCAGGTTAGA TAAAAGTGCA ACCAACCAGC AACAACCCCG
Makindu_22        GAAGCAACAA CCTAAGCAAG CCAGGTTAGA TAAAAGTGCA ACCAACCAGC AACAACCCCA
Canberra_5        GAAGCAACAA CCTAAGCAAG CCAGGTTAGA TAAAAGTGCA ACCAACCAGC AACAACCCCA
Cann-River_2      GAAGCAACAA CCTAAGCAAG CCAGGTTAGA TAAAAGTGCA ACCAACCAGC AACAACCCCA
Cann-River_6      GAAGCAACAA CCTAAGCAAG CCAGGTTAGA TAAAAGTGCA ACCAACCAGC AACAACCCCA
Cann-River_7      GAAGCAACAA CCTAAGCAAG CCAGGTTAGA TAAAAGTGCA ACCAACCAGC AACAACCCCA
Cann_River_5      GAAGCAACAA CCTAAGCAAG CCAGGTTAGA TAAAAGTGCA ACCAACCAGC AACAACCCCA
Cann-River_8      GAAGCAACAA CCTAAGCAAG CCAGGTTAGA TAAAAGTGCA ACCAACCAGC AACAACCCCA
Valence_5         GAAGCAACAA CCTAAGCAAG CCAGGTTAGA TAAAAGTGCA ACCAACCAGC AACAACCCCA
Valence_9         GAAGCAACAA CCTAAGCAAG CCAGGTTAGA TAAAAGTGCA ACCAACCAGC AACAACCCCA
Eden_1            GAAGCAACAA CCTAAGCAAG CCAGGTTAGA TAAAAGTGCA ACCAACCAGC AACAACCCCA
Valence_13        GAAGCAACAA CCTAAGCAAG CCAGGTTAGA TAAAAGTGCA ACCAACCAGC AACAACCCCA
Eden_7            GAAGCAACAA CCTAAGCAAG CCAGGTTAGA TAAAAGTGCA ACCAACCAGC AACAACCCCA
Valence_4         GAAGCAACAA CCTAAGCAAG CCAGGTTAGA TAAAAGTGCA ACCAACCAGC AACAACCCCA
Valence_2         GAAGCAACAA CCTAAGCAAG CCAGGTTAGA TAAAAGTGCA ACCAACCAGC AACAACCCCA
Valence_1         ---------- ---------- ---------- ---------- ---------- ----------
Valence_7         ---------- ---------- ---------- ---------- ---------- ----------
Makindu_cM1       GAAGCAACAA CCTAAGCAAG CCAGGTTAGA TAAAAGTGCA ACCAACCAGC AACAACCCCA
Makindu_cM6       GAAGCAACAA CCTAAGCAAG CCAGGTTAGA TAAAAGTGCA ACCAACCAGC AACAACCCCA
Makindu_1         GAAGCAACAA CCTAAGCAAG CCAGGTTAGA TAAAAGTGCA ACCAACCAGC AACAACCCCA
Amieu_5           GAAGCAACAA CCTAAGCAAG CCAAGTTTGA TAAAAGTGCA ACAAACCAAC AACAACCCCA
Eden_11           GAAGCAACAA CCTAAGCAAG ACAGGTTAGA TAAAAGTGCA ACAAACCAGC AACAACCCCA
Cann-River_10     GAAGCAACAA CCTAAGCAAG CCAGGTTAGA TAAAAGTGCA ACAAACCAGC AACAACCCCA
Brazzaville_10    GAAGCAACAA CCTAAGCAAG CCAGGTTAGA TAAAAGTGCA ACAAACCAGC AACAACCCCA
Cann-River_3      GAAGCAACAA CCTAAGCAAG CCAGGTTTGA TAAAAGTGCA ACAAATCAAC AACAACCCCA
Cann-River_13     GAAGCAACAA CCTAAGCAAG ACAGGTTAGA TAAAAGTGCA ACAAACCAGC AACAACCCCA
Eden_4            GAAGCAACAA CCTAAGCAAG ACAGGTTAGA TAAAAGTGCA ACAAACCAGC AACAACCCCA
Makindu_10        GAAGCAACAA CCTAAGCAAG ACAGGTTAGA TAAAAGTGCA ACAAACCAGC AACAACCCCA
Makindu_11        GAAGCAACAA CCTAAGCAAG ACAGGTTAGA TAAAAGTGCA ACAAACCAGC AACAACCCCA
Brazzaville_11    GAAGCAACAA CCTAAGCAAG CCAGGTTAGA TAAAAGTGCA ACCAACCAGC AACAACCCCA
Brazzaville_12    GAAGCAACAA CCTAAGCAAG CCAGGTTAGA TAAAAGTGCA ACCAACCAGC AACAACCCCA
Brazzaville_8     GAAGCAACAA CCTAAGCAAG CCAGGTTAGA TAAAAGTGCA ACCAACCAGC AACAACCCCA
chrU_963982       ---------- ---------- ---------- ---------- ---------- ----------
chr3L_18642496    ---------- ---------- ---------- ---------- ---------- ----------
chrU_3975907      GAAGCAACAA CCTAAGCAAG CCAGGTTAGA TAAAAGTGCA ACAAACCAGC AACAACCCCA
chrU_10625041     GAAGCAACAA CCTAAGCAAG CCAGGTTAGA TAAAAGTGCA ACAAACCAGC AACA-CCCCA
chrU_2316095      GAAGCAACAA CCTAAGCAAG CCAGGTTAGA TAAAAGTGCA ACAAACCAGC AACAACCCCA
chrU_4537377      GAAGCAACAA CCTAAGCAAG CCAGGTTTGA TAAAAGTGCA ACAAACCAAC AACAACCCCA
chrU_5384045      GAAGCAACAA CCTAAGCAAG CCAGGTTAGA TAAAAGTGCA ACCAACCAGC AACAACCCCA
chrU_5680622      ---------- ---------- ---------- ---------- ---------- ----------
chrU_5950518      GAAGCAACAA CTTAAGCAAG CCAGGTTAGA TA-------- ---------G CAACACCCCA
chrU_6132643      ---------- ---------- ---------- ---------- ---------- ----------
chrX_16602314     GAAGCTACAA CCTAAGCAAG CCAGGTTAGA TAAAAGTGCA ACCAACCAGC AACAACCCCA
chrU_6536133      GAAGCAACAA CCTAAGCAAG ACATGTTAGA TAAAAGTGCA ACAAACCAAT AAGAACCCCA
```

```
                  1321
chr3R_1506433     GCAAAAACAG CAGCATGCAA TAGCTAGCAC TCCCAAAGAT CATACTGGGA GCTTGTCCTA
Brazzaville_16    GCAAAAACAG CAGCATACAA TAGCTAGCAC TCCCAAAGGT CATACTGGGA GCTTGTCCTA
Eden_3            GCAAAAACAG CAGCATACAA TAGAAGGCAC TCCCAAAGAT CATATTGGGA GCTTGTCCTA
Cann-River_9      GCGAAAACAG CAGCATACAA TAGAAGGCAC TCCCAAAGAT CATATTGGGA GCTTGTCCTA
Amieu_2           GCAAAAACAG CAGCATGCAA TAGCTAGC-- ---------- ---------- ----------
Makindu_2         GCAAAAACAG CAGCATACAA TAGCTAGCAC TCCCAAAGGT CATACTGGGA GCTTGTCCTA
Makindu_6         GCAAAAACAG CAGCATACAA TAGCTAGCAC TCCCAAAGGT CATACTGGGA GCTTGTCCTA
Makindu_7         GCAAAAACAG CAGCATACAA TAGCTAGCAC TCCCAAAGGT CATACTGGGA GCTTGTCCTA
Valence_6         GCAAAAACAG CAGCATACAA TAGCTAGCAC TCCCAAAGGT CATACTGGGA GCTTGTCCTA
Makindu_8         GCAAAAACAG CAGCATACAA TAGCTAGCAC TCCCAAAGGT CATACTGGGA GCTTGTCCTA
Makindu_22        GCAAAAACAG CAGCATACAA TAGCTAGCAC TCCCAAAGGT CATACTGGGA GCTTGTCCTA
Canberra_5        GCAAAAACAG CAGCATACAA TAGCTAGCAC TCCCAAAGGT CATACTGGGA GCTTGTCCTA
Cann-River_2      GCAAAAACAG CAGCATACAA TAGCTAGCAC TCCCAAAGGT CATACTGGGA GCTTGTCCTA
Cann-River_6      GCAAAAACAG CAGCATACAA TAGCTAGCAC TCCCAAAGGT CATACTGGGA GCTTGTCCTA
Cann-River_7      GCAAAAACAG CAGCATACAA TAGCTAGCAC TCCCAAAGGT CATACTGGGA GCTTGTCCTA
Cann_River_5      GCAAAAACAG CAGCATACAA TAGCTAGCAC TCCCAAAGGT CATACTGGGA GCTTGTCCTA
Cann-River_8      GCAAAAACAG CAGCATACAA TAGCTAGCAC TCCCAAAGGT CATACTGGGA GCTTGTCCTA
Valence_5         GCAAAAACAG CAGCATACAA TAGCTAGCAC TCCCAAAGGT CATACTGGGA GCTTGTCCTA
Valence_9         GCAAAAACAG CAGCATACAA TAGCTAGCAC TCCCAAAGGT CATACTGGGA GCTTGTCCTA
Eden_1            GCAAAAACAG CAGCATACAA TAGCTAGCAC TCCCAAAGGT CATACTGGGA GCTTGTCCTA
Valence_13        GCAAAAACAG CAGCATACAA TAGCTAGCAC TCCCAAAGGT CATACTGGGA GCTTGTCCTA
Eden_7            GCAAAAACAG CAGCATACAA TAGCTAGCAC TCCCAAAGGT CATACTGGGA GCTTGTCCTA
Valence_4         GCAAAAACAG CAGCATACAA TAGCTAGCAC TCCCAAAGGT CATACTGGGA GCTTGTCCTA
Valence_2         GCAAAAACAG CAGCATACAA TAGCTAGCAC TCCCAAAGGT CATACTGGGA GCTTGTCCTA
Valence_1         ---------- ---------- ---------- ---------- ---------- ----------
Valence_7         ---------- ---------- ---------- ---------- ---------- ----------
Makindu_cM1       GCAAAAACAG CAGCATACAA TAGCTAGCAC TCCCAAAGGT CATACTGGGA GCTTGTCCTA
Makindu_cM6       GCAAAAACAG CAGCATACAA TAGCTAGCAC TCCCAAAGGT CATACTGGGA GCTTGTCCTA
Makindu_1         GCAAAAACAG CAGCATACAA TAGCTAGCAC TCCCAAAGGT CATACTGGGA GCTTGTCCTA
Amieu_5           GCAAAAACAG CAGCATGCAA TAGCTAGCAC TCCCAAAGAT CATACTGGGA GCTTGTCCTA
Eden_11           GCAAAAACAG CAGCATGCAA TAGCTAGCAC TCCCAAAAAG AATACTGGGA GCTTGTCCTA
Cann-River_10     GCAAAAACAG CAGCATGCAA TAGCTAGCAC TCCCAAAGAT CATACTGGGA GCTTGTGCTA
Brazzaville_10    GCAAAAACAG CAGCATGCAA TAGCTAGCAC TCCCAAAGAT CATACTGGGA GCTTGTGCTA
Cann-River_3      GCAAAAACAG CAGCATGCAA TAGCTAGCAC TCCCAAAGAT CATACTGGGT GCTTGTCCTA
Cann-River_13     GCAAAAACAG CAGCATGCAA TAGCTAGCAC TCCCAAAAAG AATACTGGGA GCTTGTCCTA
Eden_4            GCAAAAACAG CAGCATGCAA TAGCTAGCAC TCCCAAAAAG AATACTGGGA GCTTGTCCTA
Makindu_10        GCAAAAACAG CAGCATGCAA TAGCTAGCAC TCCCAAAAAG AATACTGGGA GCTTGTCCTA
Makindu_11        GCAAAAACAG CAGCATGCAA TAGCTAGCAC TCCCAAAAAG AATACTGGGA GCTTGTCCTA
Brazzaville_11    GCAAAAACAG CAGCATACAA TAGCTAGCAC TCCCAAAGGT CATACTGGGA GCTTGTCCTA
Brazzaville_12    GCAAAAACAG CAGCATACAA TAGCTAGCAC TCCCAAAGGT CATACTGGGA GCTTGTCCTA
Brazzaville_8     GCAAAAACAG CAGCATACAA TAGCTAGCAC TCCCAAAGGT CATACTGGGA GCTTGTCCTA
chrU_963982       ---------- ---------- ---------- ---------- ---------- ----------
chr3L_18642496    ---------- ---------- ---------- ---------- ---------- ----------
chrU_3975907      GCAAAAACAG CAGCATGCAA TAGCTAGCAC TCCCAAAGAT CATACTGGGA GCTTGTGCTA
chrU_10625041     GCAAAAACAG TAGCATGCAA TAGCTAGCAC TCCCAAA--T CATACTGGAT GCATGTCCTA
chrU_2316095      GCAAAAACAG CAGCATGCAA TAGCTAGCAC TCCCAAAGAT CATACTGGGA GCTTGTGCTA
chrU_4537377      GCAAAAACAG CAGCATGCAA TAGCTAGCAC TCCCAAAGAT CATACTGGGA GCTTGTCCTA
chrU_5384045      GCAAAAACAG CAGCATACAA TAGAAGGCAC TCCCAAAGAT CATATTGGGA GCTTGTCCTA
chrU_5680622      ---------- ---------- ---------- ---------- ---------- ----------
chrU_5950518      GCAAAAACAG CAGCATGCAA TAGCTAGCAC TCCCAAAGAT CATACTGGAT GCTTGTCCTA
chrU_6132643      ---------- ---------- ---------- ---------- ---------- ----------
chrX_16602314     GCAAAAACAG CAGCATACAA TAGCTAGCAC TCCCAAAGAT CATACTGGGA GCTTGTCCTA
chrU_6536133      GCAAAAACAG CAGCATGCAA TAGCTAGCAC TCCCAAGGCC CATAGTTGTA CCTTGTCCTA
```

```
                   1381
chr3R_1506433      CGCAGATATT GCAAGAAATG GCAATACAAC AGCCCAGCCT CGTCTACATA ATGTACAATT
Brazzaville_16     CGCAGATATT GCAAGAAATG GCAATACAAC AGCCCAGCCT CGTCTACATA ATGTACAATT
Eden_3             CGCAGATATT GCAAGAAATG GCAATACAAC AGCCCAGCCT CGTCTACATA ATGTACAATT
Cann-River_9       CGCAGATGTT GCAAGAAATG GCAATACAAC AGCCCAGCCT CGTCTACATA ATGTACAATT
Amieu_2            ---------- ---------- ---------- ---------- ---------- ----------
Makindu_2          CGCAGATATT GCAAGAAATG GCAATACAAC AGCCCAGCCT CGTCTACATA ATGTACAATT
Makindu_6          CGCAGATATT GCAAGAAATG GCAATACAAC AGCCCAGCCT CGTCTACATA ATGTACAATT
Makindu_7          CGCAGATATT GCAAGAAATG GCAATACAAC AGCCCAGCCT CGTCTACATA ATGTACAATT
Valence_6          CGCAGATATT GCAAGAAATG GCAATACAAC AGCCCAGCCT CGTCTACATA ATGTACAATT
Makindu_8          CGCAGATATT GCAAGAAATG GCAATACAAC AGCCCAGCCT CGTCTACATA ATGTACAATT
Makindu_22         CGCAGATATT GCAAGAAATG GCAATACAAC AGCCCAGCCT CGTCTACATA ATGTACAATT
Canberra_5         CGCAGATATT GCAAGAAATG GCAATACAAC AGCCCAGCCT CGTCTACATA ATGTACAATT
Cann-River_2       CGCAGATATT GCAAGAAATG GCAATACAAC AGCCCAGCCT CGTCTACATA ATGTACAATT
Cann-River_6       CGCAGATATT GCAAGAAATG GCAATACAAC AGCCCAGCCT CGTCTACATA ATGTACAATT
Cann-River_7       CGCAGATATT GCAAGAAATG GCAATACAAC AGCCCAGCCT CGTCTACATA ATGTACAATT
Cann_River_5       CGCAGATATT GCAAGAAATG GCAATACAAC AGCCCAGCCT CGTCTACATA ATGTACAATT
Cann-River_8       CGCAGATATT GCAAGAAATG GCAATACAAC AGCCCAGCCT CGTCTACATA ATGTACAATT
Valence_5          CGCAGATATT GCAAGAAATG GCAATACAAC AGCCCAGCCT CGTCTACATA ATGTACAATT
Valence_9          CGCAGATATT GCAAGAAATG GCAATACAAC AGCCCAGCCT CGTCTACATA ATGTACAATT
Eden_1             CGCAGATATT GCAAGAAATG GCAATACAAC AGCCCAGCCT CGTCTACATA ATGTACAATT
Valence_13         CGCAGATATT GCAAGAAATG GCAATACAAC AGCCCAGCCT CGTCTACATA ATGTACAATT
Eden_7             CGCAGATATT GCAAGAAATG GCAATACAAC AGCCCAGCCT CGTCTACATA ATGTACAATT
Valence_4          CGCAGATATT GCAAGAAATG GCAATACAAC AGCCCAGCCT CGTCTACATA ATGTACAATT
Valence_2          CGCAGATATT GCAAGAAATG GCAATACAAC AGCCCAGCCT CGTCTACATA ATGTACAATT
Valence_1          ---------- ---------- ---------- ---------- ---------- ----------
Valence_7          ---------- ---------- ---------- ---------- ---------- ----------
Makindu_cM1        CGCAGATATT GCAAGAAATG GCAATACAAC AGCCCAGCCT CGTCTACATA ATGTACAATT
Makindu_cM6        CGCAGATATT GCAAGAAATG GCAATACAAC AGCCCAGCCT CGTCTACATA ATGTACAATT
Makindu_1          CGCAGATATT GCAAGAAATG GCAATACAAC AGCCCAGCCT CGTCTACATA ATGTACAATT
Amieu_5            CGCAGATATT GCAAGAAATG GCAATACAAC AGCCCAGCCT CGTCTACATA ATGTATAATT
Eden_11            CGCAGATATT GCAAGAAATG GCAATACAAC AGTCCAGCCT GGTCTACATA ATGTACAATT
Cann-River_10      CGCAGATATT GCAAGAAATG GCAATACAAC AGCCCAGTCT CGTCTACATA ATGTACAATT
Brazzaville_10     CGCAGATATT GCAAGAAATG GCAATACAAC AGCCCAGTCT CGTCTACATA ATGTACAATT
Cann-River_3       CGCAGATATT GCAAGAAATG GCAATACAAC AGCCCAGCCT CGTCTACATA ATGTACAATT
Cann-River_13      CGCAGATATT GCAAGAAATG GCAATACAAC AGTCCAGCCT GGTCTACATA ATGTACAATT
Eden_4             CGCAGATATT GCAAGAAATG GCAATACAAC AGTCCAGCCT GGTCTACATA ATGTACAATT
Makindu_10         CGCAGATATT GCAAGAAATG GCAACACAAC AGTCCAGCCT GGTCTACATA ATGTACAATT
Makindu_11         CGCAGATATT GCAAGAAATG GCAACACAAC AGTCCAGCCT GGTCTACATA ATGTACAATT
Brazzaville_11     CGCAGATATT GCAAGAAATG GCAATACAAC AGCCCAGCCT CGTCTACATA ATGTGCAATT
Brazzaville_12     CGCAGATATT GCAAGAAATG GCAATACAAC AGCCCAGCCT CGTCTACATA ATGTACAATT
Brazzaville_8      CGCAGATATT GCAAGAAATG GCAATACAAC AGCCCAGCCT CGTCTACATA ATGTACAATT
chrU_963982        ---------- ---------- ---------- ---------- ---------- ----------
chr3L_18642496     ---------- ---------- ---------- ---------- ---------- ----------
chrU_3975907       CGCAGATATT GCAAGAAATG GCAATACAAC AGCCCAGTCT CGTCTACATA ATGTACAATT
chrU_10625041      CGCAGATATT GCAAGAAATG GCAATACAAC AGCCCAGCCT CGTCTACATA ATGTACAATT
chrU_2316095       CGCAGATATT GCAAGAAATG GCAATACAAC AGCCCAGTCT CGTCTACATA ATGTACAATT
chrU_4537377       CGCAGATATT GCAAGAAATG GCAATACAAC AGCCCAGCCT CGTCTACATA ATGTACAATT
chrU_5384045       CGCAGATATT GCAAGAAATG GCAATACAAC AGCCCAGCCT CGTCTACATA ATGTACAATT
chrU_5680622       ---------- ---------- ---------- ---------- ---------- ----------
chrU_5950518       CGCAGATATT GCAAGAAATG GCAATACAAC AGCCCAGCCT CGTCTACAAA ATGTACAATT
chrU_6132643       ---------- ---------- ---------- ---------- ---------- ----------
chrX_16602314      CGCAGATATT GCAAGAAATG GCAATACAAC AGCCCAGCCT CGTCTACATA ATGTACAATT
chrU_6536133       CGCAGCTATT ACAA-AAATG GCAATACAAC GGCACAGCCT CGTCTACGTA ATGTACAATT
```

```
                  1441
chr3R_1506433     AAAGGGAACT AATATTAAGC AGCAACACCC GCTTGACGTT CAATCAATAT TGGCACAGCA
Brazzaville_16    AAAGGGAACT AAGATTAAGC AGCCACACCC GCTTGACGTT CAGTCAATAT TGGCACAGCA
Eden_3            ACAGGGAACT AATATTAAGC AGCAACATCC GCTTGACGTT CAGTCAATAT TGGCACAGCA
Cann-River_9      ACAGGGAACT AATATTAAGC AGCAACATCC GCTTGACGTT CAGTCAATAT TGGCACAGCA
Amieu_2           ---------- ---------- ---------- ---------- ---------- ----------
Makindu_2         AAA------- ---------- ---------- ---------- ---------- ----------
Makindu_6         AAAGGGAACT AAAATTAAGC AGCCACACCC ---------- ---------- ----------
Makindu_7         AAAGGGAACT AAAATTAAGC AGCCACACCC GCTTGACGTT CAGTCAATAT TGGCACAGCA
Valence_6         AAAGGGAACT AAAATTAAGC AGCCACACCC GCTTGACGTT CAGTCAATAT TGGCACAGCA
Makindu_8         AAAGGGAACT AAAATTAAGC AGCCACACCC GCTTGACGTT CAGTCAATAT TGGCACAGCA
Makindu_22        AAAGGGAACT AAAATTAAGC AGCCACACCC GCTTGACGTT CAGTCAATAT TGGCACAGCA
Canberra_5        AAAGGGAACT AAAATTAAGC AGCCACACCC GCTTGACGTT CAGTCAATAT TGGCACAGCA
Cann-River_2      AAAGGGAACT AAGATTAAGC AGCCACACCC GCTTGACGTT CAGTCAATAT TGGCACAGCA
Cann-River_6      AAAGGGAACT AAAATTAAGC AGCCACACCC GCTTGACGTT CAGTCAATAT TGGCACAGCA
Cann-River_7      AAAGGGAACT AAAATTAAGC AGCCACACCC GCTTGACGTT CAGTCAATAT TGGCACAGCA
Cann_River_5      AAAGGGAACT AAAATTAAGC AGCCACACCC GCTTGACGTT CAGTCAATAT TGGCACAcgA
Cann-River_8      AAAGGGAACT AAAATTAAGC AGCCACACCC GCTTGGCGTT CAGTCAATAT TGGCACAGCA
Valence_5         AAAGGGAACT AAAATTAAGC AGCCACACCC GCTTGACGTT CAGTCAATAT ----------
Valence_9         AAAGGGAACT AAAATTAAGC AGCCACACCC GCTTGACGTT CAGTCAATAT TGGCACA---
Eden_1            AAAGGGAACT AAAATTAAGC AGCCACACCC GCTTGACGTT CAGTCAATAT TGGCACAGCA
Valence_13        AAAGGGAACT AAAATTAAGC AGCCACACCC GCTTGACGTT CAGTCAATAT TGGCAC----
Eden_7            AAAGGGAACT AAAATTAAGC AGCCACACCC GCTTGACGTT CAGTCAATAT TGGCACtGCA
Valence_4         AAAGGGAACT AAAATTAAGC AGCCACACCC GCTTGACGTT CAGTCAATAT TGGCACAGCA
Valence_2         AAAGGGAACT AAAATTAAGC AGCCACACCC GCTTGACGTT CAGTCAATAT TGGCACAGCA
Valence_1         ---------- ---------- ---------- ---------- ---------- ----------
Valence_7         ---------- ---------- ---------- ---------- ---------- ----------
Makindu_cM1       AAAGGGAACT AAAATTAAGC AGCCACACCC GCTTGACGTT CAGTCAATAT TGGCACAGCA
Makindu_cM6       AAAGGGAACT AAAATTAAGC AGCCACACCC GCTTGACGTT CAGTCAATAT TGGCACAGCA
Makindu_1         AAAGGGAACT AAAATTAAGC AGCCACACCC GCTTGACGTT CAGTCAATAT TGGCACAGCA
Amieu_5           AAAGGGAACT AATATTAAGC AGCAACACCC GCTTGACGTT CAATCAATAT TGGCACAGCA
Eden_11           AAAGGAAACT AATATTAAGC AGCAACACCC GCTTGACGTT CATTCAATAT TGGCACAGCA
Cann-River_10     AAAGGGAACT AATATTAAGC AGCAACACCC GCTTGACGTT CAGTCAATAT TGGCACAGCA
Brazzaville_10    AAAGGGAACT AATATTAAGC AGCAACACCC GC-------- ---------- ----------
Cann-River_3      AAAGGGAACT AATATTAAGC AGCAACACCC GCTTGACGTT CAGTCAATAT TGGCACAGCA
Cann-River_13     AAAGGAAACT AATATTAAGC AGCAACACCC GCTTGACGTT CATTCAATAT TGGCACAGCA
Eden_4            AAAGGAAACT AATATTAAGC AGCAACACCC GCTTGACGTT CATTCAATAT TGGCACAGCA
Makindu_10        AAAGGAAACT AATATTAAGC AGCAACACCC GCTTGACGTT CATTCAATAT TGGCACAGCA
Makindu_11        AAAGGAAACT AATATTAAGC AGCAACACCC GCTTGACGTT CATTCAATAT TGGCACAGC-
Brazzaville_11    AAAGGGAACT AAAATTAAGC AGCCACACCC GCTTGACGTT CAGTCAATAT TGGCACAGCA
Brazzaville_12    AAAGGGAACT AAAATTAAGC AGCCACACCC GCTTGACGTT CAGTCAATAT TGGCACAGCA
Brazzaville_8     AAAGGGAACT AAAATTAAGC AGCCACACCC GCTTGACGTT CAGTCAATAT TGGCACAGCA
chrU_963982       ---------- ---------- ---------- ---------- ---------- ----------
chr3L_18642496    ---------- ---------- ---------- ---------- ---------- ----------
chrU_3975907      AAAGGGAACT AATATTAAGC AGCAACACCC GCTTGACGTT CAGTCAATAT TGGCACAGCA
chrU_10625041     AAAGGGAATT AATATTAAAC AGCAACACCC GCTTGACGTT CAATCAATAT TGGCACAGCA
chrU_2316095      AAAGGGAACT AATATTAAGC AGCAACACCC GCTTGACGTT CAGTCAATAT TGGCACAGCA
chrU_4537377      AAAGGGAACT AATATTAAGC AGCAACACCC GCTTGACGTT CAGTCAATAT TGGCACAGCA
chrU_5384045      ACAGGGAACT AATATTAAGC AGCAACATCC GCTTGACGTT CAGTCAATAT TGGCACAGCA
chrU_5680622      ---------- ---------- ---------- ---------- ---------- ----------
chrU_5950518      AAAGGGAATT AATATTAAAC AGCAACACCC GCTTGACGTT CAGTCAATAT CGGCACAGCA
chrU_6132643      ---------- ---------- ---------- ---------- ---------- ----------
chrX_16602314     AAAGGGAACT AATATTAAGC AGCAACACCC GCTTGACGTT CAGTCAATAT TGGCACAGCA
chrU_6536133      AAAGGGAATT GATATTAAAC AGTATCACCC GCTTGAAGTT CAGTCAATAT TAGCACAGCA
```

```
                 1501
chr3R_1506433    ACAGGAACAA TTTATGAA-- ----GTGGC
Brazzaville_16   ACAGGAACAA TTTATGAA-- ----GTGGC
Eden_3           ACAGGAACAA TTTA------ ---------
Cann-River_9     ACAGGAACAA TTTATGAA-- ----GTGG-
Amieu_2          ---------- ---------- ---------
Makindu_2        ---------- ---------- ---------
Makindu_6        ---------- ---------- ---------
Makindu_7        ACAGGAACAA TTTATGAA-- ----GTGGC
Valence_6        ACAGGAAGAA TTTATGAA-- ----GTGGC
Makindu_8        ACAGGAACAA TTTATGAA-- ----GTGGC
Makindu_22       ACAGGAACAA TTTATGAA-- ----GTGGC
Canberra_5       ACAGGAACAA TTTATGAA-- ----GTGGC
Cann-River_2     ACAGGAACAA TTTATGAA-- ----GTGGC
Cann-River_6     ACAGGAACAA TTTATGAA-- ----GTGGC
Cann-River_7     ACAGGAACAA TTTATGAA-- ----GTGGC
Cann_River_5     ACAGGAACAA TTTATGAA-- ----GTGGC
Cann-River_8     ACAGGAACAA TTTATGAA-- ----GTGGC
Valence_5        ---------- ---------- ---------
Valence_9        ---------- ---------- ---------
Eden_1           ACAGGAACAA TTTATGAA-- ----GTGGC
Valence_13       ---------- ---------- ---------
Eden_7           ACAGGAACAA TTTATGAA-- ----GTGGC
Valence_4        ACAGGAAGAA TTTATGAA-- ----GTGGC
Valence_2        ACAGGAAGAA TTTATGAA-- ----GTGGC
Valence_1        ---------- ---------- ---------
Valence_7        ---------- ---------- ---------
Makindu_cM1      ACAGGAAGAA TTTATGAA-- ----GTGGC
Makindu_cM6      ACAGGAAGAA TTTATGAA-- ----GTGGC
Makindu_1        ACAGGAACAA TTTATGAA-- ----G----
Amieu_5          ACAGGAACAA TTTATGAA-- ----GTGGC
Eden_11          ACAGGAACAA TTTATGAA-- ----GaGGC
Cann-River_10    ACAGGAACAA TTTATGAA-- ----G----
Brazzaville_10   ---------- ---------- ---------
Cann-River_3     GCAGGAACAA TTTATGAA-- ----G----
Cann-River_13    ACAGGAACAA TTTATGAA-- ----GTG--
Eden_4           ACAGGAACAA TTTATGAA-- ----GTGG-
Makindu_10       ACAGGAACAA TTTATGAA-- ----GTGGC
Makindu_11       ---------- ---------- ---------
Brazzaville_11   ACAGGAACAA TTTATGAA-- ----GTGGC
Brazzaville_12   ACAGGAACAA ---------- ---------
Brazzaville_8    ACAGGAAC-- ---------- ---------
chrU_963982      ---------- ---------- ---------
chr3L_18642496   ---------- ---------- ---------
chrU_3975907     ACAGGAACAA TTTATGAA-- ----GTGGC
chrU_10625041    GCAGGAACAA TTTATGAATT GTTAACTGA
chrU_2316095     ACAGGAACAA TTTATGAA-- ----GTGGC
chrU_4537377     ACAGGAACAA TTTATGAA-- ----GTGGC
chrU_5384045     ACAGGAACAA TTTATGAA-- ----TTGGC
chrU_5680622     ---------- ---------- ---------
chrU_5950518     GCAGGAACAA TTTATGAA-- ----GTGGC
chrU_6132643     ---------- ---------- ---------
chrX_16602314    ACAGAAACAA TTTATGAA-- ----GTGGC
chrU_6536133     GCATGAACAA TATACGAA-- ----TTGGC
```
