# Supplementary material for: Losing helena: The extinction of a drosophila line-like element
Source: BMC Genomics. 2008 Mar 31;9:149. doi: 10.1186/1471-2164-9-149 (PMC2330053; doi:10.1186/1471-2164-9-149)
Supplement: Additional File 5 — Alignment of helena ORF2. The data provided the alignement used to construct the tree on Figure 5 [file 1471-2164-9-149-S5.pdf]

Alignment: /home/lerat/MANIP/simulans/HELENA/ARTICLE/revision/alignment_ORF2.ali
Seaview [blocks=10 fontsize=10 A4] on Fri Dec 21 10:15:34 2007

```
                    1
chr3R_1506433_ORF2  ATCTCCTTGT TTTCAGACAA CATCTGGAAA ACTCTCTCCA ATTAAATACG GTTCTGAGCT
Canberra_82         ---------- ---------- ---------- -CTCTTTCCA ATTAAATACG CTTCTGAGCT
Zimbabwe_71         ---------- ---------- ------GAAA ACTCTTTCCA ATTAAATACG CTTCTGAGCT
Valence_6           ---------- ---------- ---------- ---------- ATTAAATACG CTTCTGAGCT
Makindu_51          ---------- ------ACAA CATCTGGAAA ACTCTTTCCA ATTAAATACG CTTCTGAGCT
Zimbabwe_79         ---------- ---------- --------AA ACTCTTTCCA ATTAAATACG CTTCTGAGCT
Zimbabwe_77         ---------- ---------- --------AA ACTCTTTCCA ATTAAATACG CTTCTGAGCT
Zimbabwe_714        ---------- ---------- CGTCTGGAAA ACTCTTTCCA ATTAAATACG CTTCTGAGCT
Zimbabwe_715        ---------- ------ACAA CATCTGGAAA ACTCTTTCCA ATTAAATACG CTTCTGAGCT
Papeete_5           ---------- ---------- ---------- ---------- ATTAAATACG CTTCTGAGCT
Papeete_1           ---------- ---------- ---------- ---------- ATTAAATACG CTTCTGAGCT
Eden_4'14           ---------- ---------A CaTCTGGAAA ACTCTTTCCa ATTAAATACG CTTCTGAGCT
Valence_5           ---------- ---------A CATCTGGAAA ACTCTTTCCA ATTAAATACG CTTCTGAGCT
Madeira_3'5         ---------- ------ACaA CaTCTGGAAA ACTCTTTCCa ATTAAATACG CTTCTGAGCT
Madeira_3'p'1       ---------- ------ACaA CaTCTGGAAA ACTCTTTCCa ATTAAATACG CTTCTGAGCT
Madeira__3'10       ---------- -----gacaa caTCTGGAAA ACTCTTTCCa ATTAAATACG CTTCTGAGCT
Canberra_810        ---------- ---------- --TCTGGAAA ACTCTTTCCA ATTAAATACG CTTCTGAGCT
Eden_4'9            ---------- -----gACAA CaTCTGGAAA ACTCTTTCCa ATTAAATACG CTTCTGAGCT
Eden_4'4            ---------- ---------A CaTCTGGAAA ACTCTTTCCa ATTAAATACG CTTCTGAGCT
Eden_4'3            ---------- ------ACaA CaTCTGGAAA ACTCTTTCCa ATTAAATACG CTTCTGAGCT
Eden_4'11           ---------- -----gACaA CaTCTGGAAA ACTCTTTCCa ATTAAATACG CTTCTGAGCT
Eden_4'12           ---------- ------ACaA CATCTGGAAA ACTCTTTCCa ATTAAATACG CTTCTGAGCT
Amieu_1             ---------- ------ACAA CATCTGGAAA ACTCTTTCCa ATTAAATACG CTTCTGAGCT
chrU_5384045        ATCTCCTTGT TTTCAGACAA CATCTGGAAA ACTCTTTCCA ATTAAATACG CTTCTGAGCT
Zimbabwe_72         ATCTCCTTGT TTTCAGACAA CATCTGGAAA ACTCTCTCCA ATTAAATACG GTTCTGAGCT
chrU_3975907        ATCTCCTTGT TTTTAGACAA CATCTGGAAA ACTCTCTCCA ATTAAATACG GTTCTGAGCT
Canberra_87         ---------- ---------- -----GGAAA ACTCTCTCCA ATTAAATACG GTTCTGAGCT
Canberra_88         ---------- ---------- -------AAA ACTCTCTCCA ATTAAATACG GTTCTGAGCT
Canberra_83         ---------- ---------- --------AA ACTCTCTCCA ATTAAATACG GTTCTGAGCT
Valence_13          ---------- -----GACAA CATCTGGAAA ACTCTCTCCA GTTAAATACG GTTCTGAGCT
Papeete_12          ---------- ---------- -----GGAAA ACTCTCTCCa ATTAAATACG GTTCTGAGCT
Zimbabwe_710        ---------- ---------- -----GGAAA ACTCTCTCCA ATTAAATACG GTTCTGAGCT
Papeete_6           ---------- ---------- ---------- ---------- ---------- ----------
Canberra_815        ---------- ---------- CATCTGGAAA ACTCTCTCCA ATTAAATACG GTTCTGAGCT
Papeete_2           ---------- ---------- ---------- ---------- ATTAAATACG GTTCTGAGCT
Brazzaville_1       ---------- ---------- ---------- ---------- ATTAAATACG GTTCTGAGCT
Madeira_3'9         ---------- ------ACAA CATCTGGAAA ACTCTCTCCA ATTAAATACG GTTCTGAGCT
Madeira_3'8         ---------- ------ACAA CATCTGGAAA ACTCTCTCCA ATTAAATACG GTTCTGAGCT
Madeira_3'7         ---------- -----GACAA CATCTGGAAA ACTCTCTCCA ATTAAATACG GTTCTGAGCT
Eden_4'13           ---------- -----GACAA CATCTGGAAA ACTCTCTCCA ATTAAATACG GTTCTGAGCT
Madeira_4'10        ---------- ----AGACAA CATCTGGAAA ACTCTCTCCA ATTAAATACG GTTCTGAGCT
Amieu_68            ---------- ---------- CATCTGGAAA ACTCTCTCCA ATTAAATACG GTTCTGAGCT
Amieu_69            ---------- ---------- CATCTGGAAA ACTCTCTCCA ATTAAATACG GTTCTGAGCT
Amieu_4             ---------- ---------- ---------- --TCTCTCCA ATTAAATACG GTTCTAAGCT
Makindu_5'4         ---------- -------CAA CATCTGGAAA ACTCTCTTCA ATTAAATACG GTTCTGAGCT
Papeete_10          ---------- ---------- ---------- ---CTCTTCA ATTAAATACG GTTCTGAGCT
Canberra_84         ---------- -----AACAA CATCTGGAAA ACTCTCTTCA ATTAAATACG GTTCTGAGCT
Cann                ---------- ---------A CaTCTGGAGA ACTCTCTCCA ATTAAATGCG GTTCTGAGCT
Amieu_61            ---------- ---------- -------AAA ACTCTCTCCA ATTAAATACG GTTCTGAGCT
Amieu_62            ---------- ---------- ---------- ACTCTCTCCA ATTAAATACG GTTCTGAGCT
Amieu_65            ---------- ------ACAA CATCTGGAAA ACTCTCTCCA ATTAAATACG GTTCTGAGCT
chrU_10862415       ---------- ---------- ---------- ---------- ---------- ----------
chrU_13548152       ATCTCCTTGT TTTCAGACAA CATCTGGAAA AATCTCTCCA ATTAAATACG GTTCTGAGCT
chrU_1677297        ATCTCCTTGT TTTCAGACAA CATCTGGAAA ACTCTCTCCA ATTAAATACG GTTCTGAGCT
chrU_2316095        ATCTCTTTGT TTTCAAACAA CATCTGGAAA ACTCTCTCCA ATTAAATTCG GTTCTGAGCT
chrU_5679045        ---------- ---------- ---------- ---------- ---------- ----------
Amieu_8             ---------- ---------- --TCTGGAAA ACTCTCTCCA ATTAAATACG GTTCTGAGCT
Valence_14          ---------- ---------- --TCTGGGAA ACTCTCTCCA ATTAAATACG GTTCTGAGCT
Valence_2           ---------- ---------- --TCTGGAAA ACTCTCTCCA ATTAAATACG GTTCTGAGCT
Valence_4           ---------- ---------- --TCTGGAAA ACTCTCTCCA ATTAAATACG GTTCTGAGCT
Zimbabwe_76         ---------- ---------- --TCTGGAAA ACTCTCTCCA ATTAAATACG GTTCTGAGCT
Zimbabwe_712        ---------- ---------- --TCTGGAGA ACTCTCTCCA -TTAAATGCG GTTCTGAGCT
chrU_5950518        ATCTCCTTGT TTTCAGACAA CATCTGGAAA AATCTCTCCA ATTAAATACG GTTCTGAGCT
chrU_6720385        ---------- ---------- ---------- ---------- ---------- ----------
Madeira_3'2         ---------- -------CAA CATCTGGAAA ACTCTCTTCA ATTAAATACG GTTCTGAGCT
chrX_16602314       ATCTCCTTGT TTTCAGACAA CATCTGGAAA ACTCTCTCCA ATTAAATACG GTTCTAAGCT
```

```
                    61
chr3R_1506433_ORF2  CTAAGGAAGA CATCGAGAAC GCAGTAGACA GTCTAACGCA AAATATACAT A--GAGCCGC
Canberra_82         CTAAGGAAGA CATCGAAAAC GCAGTAGACA GTCTAACGCA AAATATATAT A--GATCTGC
Zimbabwe_71         CTAAGGAAGA CATCGAAAAC GCAGTAGACA GTCTAACGCA AAATATATAT A--GATCTGC
Valence_6           CTAAGGAAGG CATCGAAAAC GCAGTAGACA GTCTAACGCA AAATATATAT A--GATCTGC
Makindu_51          CTAAGGAAGA CATCGAAAAC GCAGTAGACA GTCTAACGCA AAATATATAT A--GATCTGC
Zimbabwe_79         CTAAGGAAGA CATCGAAAAC GCAGTAGACA GTCTAACGCA AAATATATAT A--GATCTGC
Zimbabwe_77         CTAAGGAAGA CATCGAAAAC GCAGTAGACA GTCTAACGCA AAATATATAT A--GATCTGC
Zimbabwe_714        CTAAGGAAGA CATCGAAAAC GCAGTAGACA GTCTAACGCA AAATATATAT A--GATCTGC
Zimbabwe_715        CTAAGGAAGA CATCGAAAAC GCAGTAGACA GTCTAACGCA AAATATATAT A--GATCTGC
Papeete_5           CTAAGGAAGA CATCGAAAAC GCAGTAGACA GTCTAACGCA AAATATATA- ---GATCTGC
Papeete_1           CTAAGGAAGA CATCGAAAAC GCAGTAGACA GTCTAACGCA AAATATATAT A--GATCTGC
Eden_4'14           CTAAGGAAGA CATCGAAAAC GCAGTAGACA GTCTAACGCA AAATATATAT A--GATCTGC
Valence_5           CTAAGGAAGA CATCGAAAAC GCAGTAGACA GTCTAACGCA AAATATATAT A--GATCTGC
Madeira_3'5         CTAAGGAAGA CATCGAAAAC GCAGTAGACA GTCTAACGCA AGATATATAT A--GATCTGC
Madeira_3'p'1       CTAAGGAAGA CATCGAAAAC GCAGTAGACA GTCTAACGCA AAATATATAT A--GATCTGC
Madeira__3'10       CTAAGGAAGA CATCGAAAAC GCAGTAGACA GTCTAACGCA AAATATATAT A--GATCTGC
Canberra_810        CTAACGAAGA CATCGAAAAC GCAGTAGACA GTCTAACGCA AAATATATAT A--GATCTGC
Eden_4'9            CTAAGGAAGA CATCGAAAAC GCAGTAGACA GTCTAACGCA AAATATATAT A--GATCTGC
Eden_4'4            CTAAGGAAGA CATCGAAAAC GCAGTAGACA GTCTAACGCA AAATATATAT A--GATCTGC
Eden_4'3            CTAAGGAAGA CATCGAAAAC GCAGTAGACA GTCTAACGCA AAATATATAT A--GATCTGC
Eden_4'11           CTAAGGAAGA CATCGAAAAC GCAGTAGACA GTCTAACGCA AAATATATAT A--GATCTGC
Eden_4'12           CTAAGGAAGA CATCGAAAAC GCAGTAGACA GTCTAACGCA AAATATATAT A--GATCTGC
Amieu_1             CTAAGGAAGA CATCGAAAAC GCAGTAGACA GTCTAACGCA AAATATATAT A--GATCTGC
chrU_5384045        CTAAGGAAGA CATCGAAAAC GCAGTAGACA GTCTAACGCA AAATATATAT A--GATCTGC
Zimbabwe_72         CTAAGGAAGA CATCGAGAAC GCAGTAGACA GTCTAACGCA AAATATACAT A--GAGCCGC
chrU_3975907        CTAAGGAAGA CATCGAGAAC GCAGTAGACA GTCTAACGCA AAATATATAT A--GAGCTGC
Canberra_87         CAAAGGAAGA CATCGAGAAC GCAGTAGACA GTCTAACGCA AAATATACAT A--GAGCCGC
Canberra_88         CAAAGGAAGA CATCGAGAAC GCAGTAGACA GTCTAACGCA AAATATACAT A--GAGCCGC
Canberra_83         CTAAGGAAGA CATCGAGAAC GCAGTAGACA GTCTAACGCA AAATATATAT A--GAGCTGC
Valence_13          CTAAGGAAGA CATCGAGAAC GCAGTAGACA GTCTAACGCA AAATATACAT A--GAGCCGC
Papeete_12          caAaggaAgA caTCGAGAAC GCAGTAGACA GTCTAACGCA AAATATaCaT A--GAGCCGC
Zimbabwe_710        CTAAGGAAGA CATCGAGAAC GCAGTAGACA GTCTAACGAA AAATATACAT A--GAGCCGC
Papeete_6           ---------- --TCGAGAAC GCAGTAGACA GTCTAACGCA AAATATATAT A--GAGCTGC
Canberra_815        CTAAGGAAGA CATCGAGAAC GCAGTAGACA GTCTAACGCA AAATATACAT A--GAGCCGC
Papeete_2           CAAAGGAAGA CATCGAGAAC GCAGTAGACA GTCTAACGCA AAATATACAT A--GAGCCGC
Brazzaville_1       CTAAGGAAGA CATCGAGAAC GCAGTAGACA GTCTAACGCA AAATATACAT A--GAGCCGC
Madeira_3'9         CAAAGGAAGA CATCGAGAAC GCAGTAGACA GTCTAACGCA AAATATACAT A--GAGCCGC
Madeira_3'8         CTAAGGAAGA CATCGAGAAC GCAGTAGACA GTCTAACGCA AAATATACAT A--GAGCCGC
Madeira_3'7         CAAAGGAAGA CATCGAGAAC GCAGTAGACA GTCTAACGCA AAATATACAT A--GAGCCGC
Eden_4'13           CTAAGGAAGA CATCGAGAAC GCAGTAGACA GTCTAACGCA AAATATACAT A--GAGCCGC
Madeira_4'10        CTAAGGAAGA CATCGAGAAC GCAGTAGACA GTCTAACGCA AAATATATAT A--GAGCTGC
Amieu_68            CTAAGGAAGA CATCGAGAAC GCAGTAGACA GTCTAACGCA AAATATACAT A--GAGCCGC
Amieu_69            CTAAGGAAGA CATCGAGAAC GCAGTAGACA GTCTAACGCA AAATATACAT A--GAGCCGC
Amieu_4             CTAAGGAAGA CATCGAGAAC GCAGTAGACA GTCTA----- --ATATATAT A--GAGCTGC
Makindu_5'4         CTAAGGAAGA CATCGAGAAC GCAGTAGACA TTCTAACGCA AAATATACAT A--GAGCTGC
Papeete_10          CTAAGGAAGA CATCGAGAAC GCAGTAGACA TTCTAACGCA AAATATATAT A--GAGCTGC
Canberra_84         CTAAGGAAGA CATCGAGAAC GCAGTAGACA TTCTAACGCA AAATATATAT A--GAGCTGC
Cann                CTAAGGAAGA CATCGAGAAC GCAGTAGACA GTCTAACGTA AAATATATAT ATAGAACTGC
Amieu_61            CAAAGGAAGA CATCGAGAAC GCAGTAGACA GTCTAACGCA AAATATACAT A--GAGCCGC
Amieu_62            CTAAGGAAGA CATCGAGAAC GCAGTAGACA GTCTAACGCA AAATATACAT A--GAGCCGC
Amieu_65            CTAAGGAAGA CATCGAGAAC GCAGTAGACA GTCTAACGCA AAATATACAT A--GAGCCGC
chrU_10862415       ---------- ---------- ---------- ---------- ---------- ----------
chrU_13548152       CTAAGGAAGA CATCGAGAAC GAAGGATACA GTCTAACGCA AAATATATAT A--GAGCTGC
chrU_1677297        CTAAGGAAGA CATCGAGAAC GCAGTAGACA GTCTAACGCA CAATATATAT A--GAGC---
chrU_2316095        CTAAGGAAGA CTTCCAGAAC GCAGTTGACA TTCTAACACA AAGTATACAT A--GAGCTGC
chrU_5679045        ---------- ---------- TTTTATCCCG CATACAAACC TTCAATACAT A--GAGCTGC
Amieu_8             CTAAGGAAGA CATCGAGAAC GCAGTAGACA GTCTAACGCA AAATATACAT A--GAGCCGC
Valence_14          CAAAGGAAGA CATCGAGAAC GCAGTAGACA GTCTAACGCA AAATATACAT A--GAGCCGC
Valence_2           CAAAGGAAGA CATCGAGAAC GCAGTAGACA GTCCAACGCA AAATATACAT A--GAGCCGC
Valence_4           CAAAGGAAGA CATCGAGAAC GCAGTAGACA GTCTAACGCA AAATATACAT A--GAGCCGC
Zimbabwe_76         CTAAGGAAGA CATCGAGAAC GCAGTAGACA GTCTAACGCA AAATATATAT A--GAGCTGC
Zimbabwe_712        CTAAGGAAGA CATCGAGAAC GCAGTAGACA GTCTAACGTA AAATATATAT ATAGAACTGC
chrU_5950518        CTAAGGAAGA CATCGAGAAC GAAGGATACA GTCTAACGCA AAATATATAT A--GAGCTGC
chrU_6720385        ---------- ---------- ---------- ---------- ---------- ----------
Madeira_3'2         CTAAGGAAGA CATCGAGAAC GCAGTAGACA TTCTAACGCA AAATATACAT A--GAGCTGC
chrX_16602314       CTAAGGAAGA CATCGAGAAC GCAGTAGACA GTCTAA---- ---TATATAT A--GAGCTGC
```

```
                    121
chr3R_1506433_ORF2  TTCTGCTTCT ACGCCGTCTG AGCCCGAGAT ACGCCCCA-G AAGTTATGGT ATTGTTCTAA
Canberra_82         TTCTGCTTCT ACGCCGACTG AGCCCGAGAT ACGTCCCA-G AAGTTATGGT GTTGTA-TAA
Zimbabwe_71         TTCTGCTTCT ACGCCGACTG AGCCCGAGAT ACGTCCCA-G AAGTTATGGT GTTGTA-TAA
Valence_6           TTCTGCTTCT ACGCCGACTG AGCCCGAGAT ACGTCCCA-G AAGTTATGGT GTTGTA-TAA
Makindu_51          TTCTGCTTCT ACGCCGACTG AGCCCGAGAT ACGTCCCA-G AAGTTATGGT ATTGTA-TAA
Zimbabwe_79         TTCTGCTTCT ACGCCGACTG AGCCCGAGAT ACGTCCCA-G AAGTTATGGT GTTGTA-TAA
Zimbabwe_77         TTCTGCTTCT ACGCCGACTG AGCCCGAGAT ACGTCCCA-G AAGTTATGGT GTTGTA-TAA
Zimbabwe_714        TTCTGCTTCT ACGCCGACTG AGCCCGAGAT ACGTCCCA-G AAGTTATGGT GTTGTA-TAA
Zimbabwe_715        TTCTGCTTCT ACGCCGACTG AGCCCGAGAT ACGTCCCA-G AAGTTATGGT GTTGTA-TAA
Papeete_5           TTCTGCTTCT ACGCCGACTG AGCCCGAGAT ACGTCCCA-G AAGTTATGGT GTTGT-----
Papeete_1           TTCTGCTTCT ACGCCGACTG AGCCCGAGAT ACGTCCCA-G AAGTTATGGT GTTGT-----
Eden_4'14           TTCTGCTTCT ACGCCGACTG AGCCCGAGAT ACGTCCCA-G AAGTTATGGT GTTGTA-TAA
Valence_5           TTCTGCTTCT ACGCCGACTG AGCCCGAGAT ACGTCCCA-G AAGTTATGGT GTTGTA-TAA
Madeira_3'5         TTCTGCTTCT ACGCCGACTG AGCCCGAGAT ACGTCCCA-G AAGTTATGGT GTTGTA-TAA
Madeira_3'p'1       TTCTGCTTCT ACGCCGACTG AGCCCGAGAT ACGTCCCA-G AAGTTATGGT ATTGTA-TAA
Madeira__3'10       TTCTGCTTCT ACGCCGACTG AGCCCGAGAT ACGTCCCA-G AAGTTATGGT ATTGTA-TAA
Canberra_810        TTCTGCTTCT ACGCCGACTG AGCCCGAGAT ACGTCCCA-G AAGTTATGGT GTTGTA-TAA
Eden_4'9            TTCTGCTTCT ACGCCGACTG AGCCCGAGAT ACGTCCCA-G AAGTTATGGT GTTGTA-TAA
Eden_4'4            TTCTGCTTCT ACGCCGACTG AGCCCGAGAT ACGTCCCA-G AAGTTATGGT GTTGTA-TAA
Eden_4'3            TTCTGCTTCT ACGCCGACTG AGCCCGAGAT ACGTCCCA-G AAGTTATGGT GTTGTA-TAA
Eden_4'11           TTCTGCTTCT ACGCCGACTG AGCCCGAGAT ACGTCCCA-G AAGTTATGGT GTTGTA-TAA
Eden_4'12           TTCTGCTTCT ACGCCGACTG AGCCCGAGAT ACGTCCCA-G AAGTTATGGT GTTGTA-TAA
Amieu_1             TTCTGCTTCT ACGCCGACTG AGCCCGAGAT ACGTCCCA-G AAGTTATGGT GTTGTA-TAA
chrU_5384045        TTCTGCTTCT ACGCCGACTG AGCCCGAGAT ACGTCCCA-G AAGTTATGGT GTTGTA-TAA
Zimbabwe_72         TTCTGCTTCT ACGCCGTCTG AGCCCGAGAT ACGCCCCA-G AAGTTATGGT ATTGTTCTAA
chrU_3975907        TTCTGCTTCT ACGCCGTCTG AGCCCGAGAT ACGCCCCA-G AAGTTATGGT ATTGTACTAA
Canberra_87         TTCTGCTTCT ACGCCGTCTG AGCCCGAGAT ACGCCCCA-G AAGTTATGGT ATTGTTCTAA
Canberra_88         TTCTGCTTCT ACGCCGTCTG AGCCCGAGAT ACGCCCCA-G AAGTTATGGT ATTGTTCTAA
Canberra_83         TTCTGCTTCT ACGCCGTCTG AGCCCGAGAT ACGCCCCA-G AAGTTATGGT ATTGTACTAA
Valence_13          TTCTGCTTCT ACGCCGTCTG AGCCCGAGAT ACGCCCCA-G AAGTTATGGT ATTGTTCTAA
Papeete_12          TTCTGCTTCT ACGCCGTCTG AGCCCGAGAT ACGCCCCA-G AAGTTATGGT ATTGTTCTAA
Zimbabwe_710        TTCTGCTTCT ACGCCGTCTG AGCCCGAGAT ACGCCCCA-G AAGTTATGGT ATTGTTCTAA
Papeete_6           TTCTGCTTCT ACGCCGTCTG AGCCCGAGAT ACGCCCCA-G AAGTTATGGT ATTGTACTAA
Canberra_815        TTCTGCTTCT ACGCCGTCTG AGCCCGAGAT ACGCCCCA-G AAGTTATGGT ATTGTTCTAA
Papeete_2           TTCTGCTTCT ACGCCGTCTG AGCCCGAGAT ACGCCCCA-G AAGTTATGGT ATTGTTCTAA
Brazzaville_1       TTCTGCTTCT ACGCCGTCTG AGCCCGAGAT ACGCCCCA-G AAGTTATGGT ATTGTTCTAA
Madeira_3'9         TTCTGCTTCT ACGCCGTCTG AGCCCGAGAT ACGCCCCA-G AAGTTATGGT ATTGTTCTAA
Madeira_3'8         TTCTGCTTCT ACGCCGTCTG AGCCCGAGAT ACGCCCCA-G AAGTTATGGT ATTGTTCTAA
Madeira_3'7         TTCTGCTTCT ACGCCGTCTG AGCCCGAGAT ACGCCCCA-G AAGTTATGGT ATTGTTCTAA
Eden_4'13           TTCTGCTTCT ACGCCGTCTG AGCCCGAGAT ACGCCCCA-G AAGTTATGGT ATTGTTCTAA
Madeira_4'10        TTCTGCTTCT ACGCCGTCTG AGCCCGAGAT ACGCCCCA-G AAGTTATGGT ATTGTACTAA
Amieu_68            TTCTGCTTCT ACGCCGTCTG AGCCCGAGAT ACGCCCCA-G AAGTTATGGT ATTGTTCTAA
Amieu_69            TTCTGCTTCT ACGCCGTCTG AGCCCGAGAT ACGCCCCA-G AAGTTATGGT ATTGTTCTAA
Amieu_4             TTCTGCTTCT ACGCCGACTG AGCCCGAGAT ACGCCCCA-G AAGTTATGGT ATTGTACTAA
Makindu_5'4         TTCTGCTTTT ACGCCGTCTG AGCC---TAT ATGCCCCA-G AAGTTATGGT ATTGTACTAA
Papeete_10          TTCTGCTTCT ACGCCGTCTG AGCC---TAT ATGCCCCA-G AAGTTATGGT ATTGTACTAA
Canberra_84         TTCTGCTTCT ACGCCGTCTG AGCC---TAT ATGCCCCA-G AAGTTATGGT ATTGTACTAA
Cann                TTCTGCTTCT ACGCCGTCTG AGCCCGAGAT ACGCCCCA-G AAGTTATGGT ATTGTACTAA
Amieu_61            TTCTGCTTCT ACGCCGTCTG AGCCCGAGAT ACGCCCCA-G AAGTTATGGT ATTGTTCTAA
Amieu_62            TTCTGCTTCT ACGCCGTCTG AGCCCGAGAT ACGCCCCA-G AAGTTATGGT ATTGTTCTAA
Amieu_65            TTCTGCTTCT ACGCCGTCTG AGCCCGAGAT ACGCCCCA-G AAGTTATGGT ATTGTTCTAA
chrU_10862415       ------TTCT ACGCCGTTTG AGCCCGAGAT ACGCCCCGGA AAGTTATGGT ATTGTTCTAA
chrU_13548152       TTCTGCTTCT ACGCCGTGTG AGCCCGAGAT ACGCCCCA-G AAGTTATGGT ATTGTACTAA
chrU_1677297        ---------- ---------- ---------- ---------- ---------- ----------
chrU_2316095        TTCTG----- ----TGTCTG AGCCTGAGAC ATGCCCCA-G AAGTTATGGT ATTGTATTAA
chrU_5679045        TTCTGCTTCT ACGCCGTCTG AGCCTGAGAT ATGCACCA-G AAGTTATGGT ATTGTACTA-
Amieu_8             TTCTGCTTCT ACGCCGTCTG AGCCCGAGAT ACGCCCCA-G AAGTTATGGT ATTGTTCTAA
Valence_14          TTCTGCTTCT ACGCCGTCTG AGCCCGAGAT ACGCCCCA-G AAGTTATGGT ATTGTTCTAA
Valence_2           TTCTGCTTCT ACGCCGTCTG AGCCCGAGAT ACGCCCCA-G AAGTTATGGT ATTGTTCTAA
Valence_4           TTCTGCTTCT ACGCCGTCTG AGCCCGAGAT ACGCCCCA-G AAGTTATGGT ATTGTTCTAA
Zimbabwe_76         TTCTGCTTCT ACGCCGTCTG AGCCCGAGAT ACGCCCCA-G AAGTTATGGT ATTGTACTAA
Zimbabwe_712        TTCTGCTTCT ACGCCGTCTA AGCCCGAGAT ACGCCCCA-G AAGTTATGGT ATTGTACTAA
chrU_5950518        TTCTGCTTCT ACGCCGTGTG AGCCCGAGAT ACGCCCCA-G AAGTTATGGT ATTGTATTAA
chrU_6720385        ---------T ACGCCGACTG AGCCCGAGAT ACGTCCCA-G AAGTTATGGT ATTGTACTAA
Madeira_3'2         TTCTGCTTTT ACGCCGTCTG AGCC---TAT ATGCCCCA-G AAGTTATGGT ATTGTACTAA
chrX_16602314       TTCTGCTTCT ACGCCGACTG AGCCCGAGAT ACGCCCCA-G AAGTAATGGT ATTGTACTAA
```

```
                181
chr3R_1506433_ORF2 CAAGAGAGGC CAGAGAACTT ATCAGAACTA AGAGACGCCT TCGAAGAAGA GCAATTCGAA
Canberra_82        A--------- ---------- ---------- ---------- ---------- ----------
Zimbabwe_71        A--------- ---------- ---------- ---------- ---------- ----------
Valence_6          A--------- ---------- ---------- ---------- ---------- ----------
Makindu_51         A--------- ---------- ---------- ---------- ---------- ----------
Zimbabwe_79        A--------- ---------- ---------- ---------- ---------- ----------
Zimbabwe_77        A--------- ---------- ---------- ---------- ---------- ----------
Zimbabwe_714       A--------- ---------- ---------- ---------- ---------- ----------
Zimbabwe_715       A--------- ---------- ---------- ---------- ---------- ----------
Papeete_5          ---------- ---------- ---------- ---------- ---------- ----------
Papeete_1          ---------- ---------- ---------- ---------- ---------- ----------
Eden_4’14          A--------- ---------- ---------- ---------- ---------- ----------
Valence_5          A--------- ---------- ---------- ---------- ---------- ----------
Madeira_3’5        A--------- ---------- ---------- ---------- ---------- ----------
Madeira_3’p’1      A--------- ---------- ---------- ---------- ---------- ----------
Madeira__3’10      A--------- ---------- ---------- ---------- ---------- ----------
Canberra_810       A--------- ---------- ---------- ---------- ---------- ----------
Eden_4’9           A--------- ---------- ---------- ---------- ---------- ----------
Eden_4’4           A--------- ---------- ---------- ---------- ---------- ----------
Eden_4’3           A--------- ---------- ---------- ---------- ---------- ----------
Eden_4’11          A--------- ---------- ---------- ---------- ---------- ----------
Eden_4’12          A--------- ---------- ---------- ---------- ---------- ----------
Amieu_1            A--------- ---------- ---------- ---------- ---------- ----------
chrU_5384045       A--------- ---------- ---------- ---------- ---------- ----------
Zimbabwe_72        CAAGAGAGGC CAGAGAACTT ATCAGAACTA AGAGACGCCT TCGAAGAAGA GCAATTCGAA
chrU_3975907       CAAGAGAGGC CAGAGAACTT ATCAGAACTA AGAGACGCCT TCGAAGAAGA GCAATTCGAA
Canberra_87        CAAGAGAGGC CAGAGAACTT ATCAGAACTA AGAGACGCCT TCGAAGAAGA GCAATTCGAA
Canberra_88        CAAGAGAGGC CAGAGAACTT ATCAGAACTA AGAGACGCCT TCGAAGAAGA GCAATTCGAA
Canberra_83        CAAGAGAGGC CAGAGAACTT ATCAGAACTA AGAGACGCCT TCGAAGAAGA GCAATTCGAA
Valence_13         CAAGAGAGGC CAGAGAACTT ATCAGAACTA AGAGACGCCT TCGAAGAAGA GCAATTCGAA
Papeete_12         CAAGAGAGGC CAGAGAACTT ATCAGAACTA AGAGACGCCT TCGAAGAAGA GCAATTCGAA
Zimbabwe_710       CAAGAGAGGC CAGAGAACTT ATCAGAACTA AGAGACGCCT TCGAAGAAGA GCAATTCGGA
Papeete_6          CAAGAGAGGC CAGAGAACTT ATCAGAACTA AGAGACGCCT TCGAAGAAGA GCAATTCGAA
Canberra_815       CAAGAGAGGC CAGAGAACTT ATCAGAACTA AGAGACGCCT TCGAAGAAGA GCAATTCGAA
Papeete_2          CAAGAGAGGC CAGAGAACTT ATCAGAACTA AGAGACGCCC TCGAAGAAGA GCAATTCGAA
Brazzaville_1      CAAGAGAGGC CAGAGAACTT ATCAGAACTA AGAGACGCCT TCGAAGAAGA GCAATTCGAA
Madeira_3’9        CAAGAGAGGC CAGAGAGCTT ATCAGAACTA AGAGACGCCT TCGAAGAAGA GCAATTCGAA
Madeira_3’8        CAAGAGAGGC CAGAGAACTT ATCAGAACTA AGAGACGCCT TCGAAGAAGA GCAATTCGAA
Madeira_3’7        CAAGAGAGGC CAGAGAACTT ATCAGAACTA AGAGACGCCT TCGAAGAAGA GCAATTCGAA
Eden_4’13          CAAGAGAGGC CAGAGAACTT ATCAGAACTA AGAGACGCCT TCGAAGAAGA GCAATTCGAA
Madeira_4’10       CAAGAGAGGC CAGAGAACTT ATCAGAACTA AGAGACGCCT TCGAAGAAGA GCAATTCGAA
Amieu_68           CAAGAGAGGC CAGAGAACTT ATCAGAACTA AGAGACGCCT TCGAAGAAGA GCAATTCGAA
Amieu_69           CAAGAGAGGC CAGAGAACTT ATCAGAACTA AGAGACGCCT TCGAAGAAGA GCAATTCGAA
Amieu_4            CAAGAGAGGC CAGAGAACTT ATCAGAACTA AGAGACGCCT TCGAAGAAGA GCAATTCGAA
Makindu_5’4        CAAGAGAGGC CAGAGAACTT ATCAAAACTA AGAGATGCCT TCGAAGAAAA GCAATTCGAA
Papeete_10         CAAGAGAGGC CAGAGAACTT ATCAAAACTA AGAGATGCCT TCGAAGAAAA GCAATTCGAA
Canberra_84        CAAGAGAGGC CAGAGAACTT ATCAAAACTA AGAGATGCCT TCGAAGAAAA GCAATTCGAA
Cann               CAAGAGAGGC CAGAGAACTT ATCAGAACTA AAAGACGCCT TCGAAGAAGA GCAATTCGAA
Amieu_61           CAAGAGAGGC CAGAGAACTT ATCAGAACTA AGAGACGCCT TCGAAGAAGA GCAATTCGAA
Amieu_62           CAAGAGAGGC CAGAGAACTT ATCAGAACTA AGAGACGCCT TCGAAGAAGA GCAATTCGAA
Amieu_65           CAAGAGAGGC CAGAGAACTT ATCAGAACTA AGAGACGCCT TCGAAGAAGA GCAATTCGAA
chrU_10862415      CAAGGGAGGC CAGAGAACTT ATCAGAACTA AGAGACGCCT TCGAAGAAGA GCAATTCGAA
chrU_13548152      TACGAGAGGC CAGAGAACTT ATCAGAACTA AGAGACGCCT TCGAAGAAGA GCAATTCGAA
chrU_1677297       ---------- --------TA TATAGAACTA AGAGACGCCT TCGAAGAAAA GCAATTCGAA
chrU_2316095       CAAGAGAGGT GAGAGAACTA ATCAGAACTA AAAGACGCCT TCGAAGAAGA GCAATTCGAA
chrU_5679045       ---------- ---------- ---------- -TTGTACTGT TCGAAGAAGA GCAATTCGAA
Amieu_8            CAAGAGAGGC CAGAGAACTT ATCAGAACTA AGAGACGCCT TCGAAGAAGA GCAATTCGAA
Valence_14         CAAGAGAGGC CAGAGAACTT ATCAGAACTA AGAGACGCCT TCGAAGAAGA GCAATTCGAA
Valence_2          CAAGAGAGGC CAGAGAACTT ATCAGAACTA AGAGACGCCT TCGAAGAAGA GCAATTCGAA
Valence_4          CAAGAGAGGC CAGAGAACTT ATCAGAACTA AGAGACGCCT TCGAAGAAGA GCAATTCGAA
Zimbabwe_76        CAAGAGAGGC CAGAGAACTT ATCAGAACTA AGAGACGCCT TCGAAGAAGA GCAATTCGAA
Zimbabwe_712       CAAGAGAGGC CAGAGAACTT ATCAGAACTA AAAGACGCCT TCGAAGAAGA GCAATTCGAA
chrU_5950518       CACGAGAGGC CAGAGAACTT ATCAGAACTA AGAGACGCCT TCGAAGAAGA GCAATTCGAA
chrU_6720385       CACGAGAGGC CAGAGAACTT ATCAGAACTA AGAGACGCCT TCGAAGAAGA GCAATTCGAA
Madeira_3’2        CAAGAGAGGC CAGAGAACTT ATCAAAACTA AGAGATGCCT TCGAAGAAAA GCAATTCGAA
chrX_16602314      CAAGAGAGGC CAGAGAACTT ATCAGAACTA AGAGACGCCT TCGAAGAAGA GCAATTCGAA
```

```
                    241
chr3R_1506433_ORF2  CTCAGGATCC ATGGGACAGA ATC----TTG TGGAACCGAG CAGCAAAGCA ACTCAAAGTC
Canberra_82         ---------- ---------- ---------- ---------- ---------- ----------
Zimbabwe_71         ---------- ---------- ---------- ---------- ---------- ----------
Valence_6           ---------- ---------- ---------- ---------- ---------- ----------
Makindu_51          ---------- ---------- ---------- ---------- ---------- ----------
Zimbabwe_79         ---------- ---------- ---------- ---------- ---------- ----------
Zimbabwe_77         ---------- ---------- ---------- ---------- ---------- ----------
Zimbabwe_714        ---------- ---------- ---------- ---------- ---------- ----------
Zimbabwe_715        ---------- ---------- ---------- ---------- ---------- ----------
Papeete_5           ---------- ---------- ---------- ---------- ---------- ----------
Papeete_1           ---------- ---------- ---------- ---------- ---------- ----------
Eden_4'14           ---------- ---------- ---------- ---------- ---------- ----------
Valence_5           ---------- ---------- ---------- ---------- ---------- ----------
Madeira_3'5         ---------- ---------- ---------- ---------- ---------- ----------
Madeira_3'p'1       ---------- ---------- ---------- ---------- ---------- ----------
Madeira__3'10       ---------- ---------- ---------- ---------- ---------- ----------
Canberra_810        ---------- ---------- ---------- ---------- ---------- ----------
Eden_4'9            ---------- ---------- ---------- ---------- ---------- ----------
Eden_4'4            ---------- ---------- ---------- ---------- ---------- ----------
Eden_4'3            ---------- ---------- ---------- ---------- ---------- ----------
Eden_4'11           ---------- ---------- ---------- ---------- ---------- ----------
Eden_4'12           ---------- ---------- ---------- ---------- ---------- ----------
Amieu_1             ---------- ---------- ---------- ---------- ---------- ----------
chrU_5384045        ---------- ---------- ---------- ---------- ---------- ----------
Zimbabwe_72         CTCAGGATCC ATGGGACAGA ATC----TTG TGGAACCGAG CAGCAAAGCA ACTCAAAGTC
chrU_3975907        CTCAGGATCC ATGGGACAGA ATC----TTG TGGAACCGAG CAGCAAAGCA ACTCAAAGTC
Canberra_87         CTCAGGATCC ATGGGACAGA ATC----TTG TGGAACCGAG CAGCAAAGCA ACTCAAAGTC
Canberra_88         CTCAGGATCC ATGGGACAGA ATC----TTG TGGAACCGAG CAGCAAAGCA ACTCAAAGTC
Canberra_83         CTCAGGATCC ATGGGACAGA ATC----TTG TGGAACCGAG CAGCAAAGCA ACTCAAAGTC
Valence_13          CTCAGGATCC ATGGGACAGA ATC----TTG TGGAACCGAG CAGCAAAGCA ACTCAAAGTC
Papeete_12          CTCAGGATCC ATGGGACAGA ATC----TTG TGGAACCGAG CAGCAAAGCA ACTCAAAGTC
Zimbabwe_710        CTCAGGATCC ATGGGACAGA ATC----TTG TGGAACCGAG CAGCAAATCA ACTCAAAGTC
Papeete_6           CTCAGGATCC ATGGGACAGA ATC----TTG TGGAACCGAG CAGCAAAGCA ACTCAAAGTC
Canberra_815        CTCAGGATCC ATGGGACAGA ATC----TTG TGGAACCGAG CAGCAAAGCA ACTCAAAGTC
Papeete_2           CTCAGGATCC ATGGGACAGA ATC----TTG TGGAACCGAG CAGCAAAGCA ACTCAAAGTC
Brazzaville_1       CTCAGGATCC ATGGGACAGA ATC----TTG TGGAACCGAG CAGCAAAGCA ACTCAAAGTC
Madeira_3'9         CTCAGGATCC ATGGGACAGA ATC----TTG TGGAACCGAG CAGCAAAGCA ACTCAAAGTC
Madeira_3'8         CTCAGGATCC ATGGGACAGA ATC----TTG TGGAACCGAG CAGCAAAGCA ACTCAAAGTC
Madeira_3'7         CTCAGGATCC ATGGGACAGA ATC----TTG TGGAACCGAG CAGCAAAGCA ACTCAAAGCC
Eden_4'13           CTCAGGATCC ATGGGACAGA ATC----TTG TGGAACCGAG CAGCAAAGCA ACTCAAAGTC
Madeira_4'10        CTCAGGATCC ATGGGACAGA ATC----TTG TGGAACCGAG CAGCAAAGCA ACTCAAAGTC
Amieu_68            CTCAGGATCC ATGGGACAGA ATC----TTG TGGAACCGAG CAGCAAAGCA ACTCAAAGTC
Amieu_69            CTCAGGATCC ATGGGACAGA ATC----TTG TGGAACCGAG CAGCAAATCA ACTCAAAGTC
Amieu_4             CTCAGGATCC TTGGGACAGA ATC----TTG TGGAACCGAG CAGCAAAGCA ACTCAAAGTC
Makindu_5'4         CTCAGGATCC ATGGCACAGA ATC----TTG TGGAGCCGAG CAGCAAAGCA ACTCAAAATC
Papeete_10          CTCAGGATCC ATGGCACAGA ATC----TTG TGGAGCCGAG CAGCAAAGCA ACTCAAAATT
Canberra_84         CTCAGGATCC ATGGCACAGA ATC----TTG TGGAGCCGAG CAGCAAAGCA ACTCAAAATT
Cann                CTCAGGATCC ATGGGACAGA ATC----TTG TGGAACCGAA CAGCAAAGCA ACTCAAAGTC
Amieu_61            CTCAGGATCC ATGGGACAGA ATC----TTG TGGAACCGAG CAGCAAAGCA ACTCAAAGTC
Amieu_62            CTCAGGATCC ATGGGACAGA ATC----TTG TGGAACCGAG CAGCAAATCA ACTCAAAGTC
Amieu_65            CTCAGGATCC ATGGGACAGA ATC----TTG TGGAACCGAG CAGCAAAGCA ACTCAAAGTC
chrU_10862415       CTCAGGATCC ATGGGACAGA ATC----TTG TGGAACCGAG CAGCAAAGCA ACTCAAAGTC
chrU_13548152       CTCAGGATCC ATGGGACAGA ATC----TTG TGGAACCGAG CAGCTAAGCA ACT-------
chrU_1677297        TTTAGGATCC ATGGGACAGA ATC----TTG TGGAACCGAG CCGCAAAGCA ACTCAAAGCC
chrU_2316095        CTCAGGATCC ATGGGACAGA ATC----TTG TGGAACCGAG CAGCAAAGCA ACTCAAAATC
chrU_5679045        CTCAGGATCC ATGGGACAGA ATCCATGGGA CAGAACCGAG CAGCAAAGCA ACTCAAAGTC
Amieu_8             CTCAGGATCC ATGGGACAGA ATC----TTG TGGAACCGAG CAGCAAAGCA ACTCAAAGTC
Valence_14          CTCAGGATCC ATGGGACAGA ATC----TTG TGGAACCGAG CAGCAAAGCA ACTCAAAGTC
Valence_2           CTCAGGATCC ATGGGACAGA ATC----TTG TGGAACCGAG CAGCAAAGCA ACTCAAAGTC
Valence_4           CTCAGGATCC ATGGGACAGA ATC----TTG TGGAACCGAG CAGCAAAGCA ACTCAAAGTC
Zimbabwe_76         CTCAGGATCC ATGGGACAGA ATC----TTG TGGAACCGAG CAGCAAAGCA ACTCAAAGTC
Zimbabwe_712        CTCAGGATCC ATGGGACAGA ATC----TTG TGGAACCGAA CAGCAAAGCA ACTCAAAGTC
chrU_5950518        CTCAGGATCC ATGGGACAGA ATC----TTG TGGAACCGAG CAGCTAAGCA ACTCAAAGTC
chrU_6720385        CTCAGGATCC ATGGGACAGA ATC----TTG TGGAACCGAG CAGCAAAGCA ACTCAAAGTC
Madeira_3'2         CTCAGGATCC ATGGCACAGA ATC----TTG TGGAGCCGAG CAGCAAAGCA ACTCAAAATC
chrX_16602314       CTCAGGATCC ATGGGACAGA ATC----TTG TGGAACCGAG CAGCAAAGCA ACTCAAAGTC
```

```
                      301
chr3R_1506433_ORF2    GTCTTAAGGG AACTCAGAAG TGATTTCTTT GAGCAAAAAT TATCCTCCAT GGACTACACC
Canberra_82           ---------- ---------- ---------- ---------- ---------- ----------
Zimbabwe_71           ---------- ---------- ---------- ---------- ---------- ----------
Valence_6             ---------- ---------- ---------- ---------- ---------- ----------
Makindu_51            ---------- ---------- ---------- ---------- ---------- ----------
Zimbabwe_79           ---------- ---------- ---------- ---------- ---------- ----------
Zimbabwe_77           ---------- ---------- ---------- ---------- ---------- ----------
Zimbabwe_714          ---------- ---------- ---------- ---------- ---------- ----------
Zimbabwe_715          ---------- ---------- ---------- ---------- ---------- ----------
Papeete_5             ---------- ---------- ---------- ---------- ---------- ----------
Papeete_1             ---------- ---------- ---------- ---------- ---------- ----------
Eden_4’14             ---------- ---------- ---------- ---------- ---------- ----------
Valence_5             ---------- ---------- ---------- ---------- ---------- ----------
Madeira_3’5           ---------- ---------- ---------- ---------- ---------- ----------
Madeira_3’p’1         ---------- ---------- ---------- ---------- ---------- ----------
Madeira__3’10         ---------- ---------- ---------- ---------- ---------- ----------
Canberra_810          ---------- ---------- ---------- ---------- ---------- ----------
Eden_4’9              ---------- ---------- ---------- ---------- ---------- ----------
Eden_4’4              ---------- ---------- ---------- ---------- ---------- ----------
Eden_4’3              ---------- ---------- ---------- ---------- ---------- ----------
Eden_4’11             ---------- ---------- ---------- ---------- ---------- ----------
Eden_4’12             ---------- ---------- ---------- ---------- ---------- ----------
Amieu_1               ---------- ---------- ---------- ---------- ---------- ----------
chrU_5384045          ---------- ---------- ---------- ---------- ---------- ----------
Zimbabwe_72           GTCTTAAGGG AACTCAGAAG TGATTTCTTT GAGCAAAAAT TATCCTCCAT GGACTACACC
chrU_3975907          ATCTTAAGGG AACTCAGAAG TGATTTCTTT GAGCAAAAAT TATCCTCCAT GGACTACACC
Canberra_87           GTCTTAAGGG AACTCAGAAG TGATTTCTTT GAGCAAAAAT TATCCTCCAT GGACTACACC
Canberra_88           GTCTTAAGGG AACTCAGAAG TGATTTCTTT GAGCAAAAAT TATCCTCCAT GGACTACACC
Canberra_83           ATCTTAAGGG AACTCAGAAG TGATTTCTTT GAGCAAAAAT TATCCTCCAT GGACTACACC
Valence_13            GTCTTAAGGG AACTCAGAAG TGATTTCTTT GAGCAAAAAT TATCCTCCAT GGACTACACC
Papeete_12            GTCTTAAGGG AACTCAGAAG TGATTTCTTT GAGCAAAAAT TATCCTCCAT GGACTACACC
Zimbabwe_710          GTCCTAAGGG AACTCAGAAG TGATTTCTTT GAGCAAAAAT TATCCTCCAT GGACTACACC
Papeete_6             ATCTTAAGGG AACTCAGAAG TGATTTCTTT GAGCAAAAAT TATCCTCCAT GGACTACACC
Canberra_815          GTCTTAAGGG AACTCAGAAG TGATTTCTTT GAGCAAAAAT TATCCTCCAT GGACTACACC
Papeete_2             GTCTTAAGGG AACTCAGAAG TGATTTCCTT GAGCAAAAAT TATCCTCCAT GGACTACACC
Brazzaville_1         GTCTTAAGGG AACTCAGAAG TGATTTCTTT GAGCAAAAAT TATCCTCCAT GGACTACACC
Madeira_3’9           GTCTTAAGGG AACTCAGAAG TGATTTCTTT GAGCAAAAAT TATCCTCCAT GGACCACACC
Madeira_3’8           GTCTTAAGGG AACTCAGAAG TGATTTCTTT GAGCAAAAAT TATCCTCCAT GGACTACACC
Madeira_3’7           GTCTTAAGGG AACTCAGAAG TGATTTCTTT GAGCAAAAAT TATCCTCCAT GGACTACACC
Eden_4’13             GTCTTAAGGG AACTCAGAAG TGATTTCTTT GAGCAAAAAT TATCCTCCAT GGACTACACC
Madeira_4’10          ATCTTAAGGG AACTCAGAAG TGATTTCTTT GAGCAAAAAT TATCCTCCAT GGACTACACC
Amieu_68              GTCTTAAGGG AACTCAGAAG TGATTTCTTT GAGCAAAAAT TATCCTCCAT GGACTACACC
Amieu_69              GTCTTAAGGG AATTCAGAAG TGATTTCTTT GAGCAAAAAT TATCCTCCAT GGACTACACC
Amieu_4               ATCTTAAGGG AACTCAGAAG TGATTTCTTT GAGCAAAAAT TATCCTCCAT GGACTAAACC
Makindu_5’4           ATCTTAAGGG AACTCAGAAG TGATTACTTT GAGCAAAAAT T--------T G----CCTGC
Papeete_10            ATCTTAAGGG AACTCAGAAG TGATTACTTT GAGCAAAAAT T--------T G----CCTGC
Canberra_84           ATCTTAAGGG AACTCAGAAG TGATTACTTT GAGCAAAAAT T--------T G----CCTGC
Cann                  ATCTTAAGGG AACTCAGAAG TGATTTCTTT GAGCAAAAAT TATCCTCCAT GGACCACACC
Amieu_61              GTCTTAAGGG AACTCAGAAG TGATTTCTTT GAGCAAAAAT TATCCTCCAT GGACTACACC
Amieu_62              GTCTTAAGGG AACTCAGAAG TGATTTCTTT GAGCAAAAAT TATCCTCCAT GGACTACACC
Amieu_65              GTCTTAAGGG AACTCAGAAG TGATTTCTTT GAGCAAAAAT TATCCTCCAT GGACTACACC
chrU_10862415         GTCTTAAGGG AACTCAGAAG TGATTTCTTT GAGCAAAAAT TATCCTCCAT GGACTACACC
chrU_13548152         ---------- ---------- ---------- ---------- ---------- ----------
chrU_1677297          ATCTTAAGCG AACTCAGAAG TGATTTCTTT GAGCAAAAAT TATTCTTCAT GGACTACACC
chrU_2316095          ATCTTAAGGG AACTCAGAAG TGATTTCATT GAGCAAAAAT TATTCTCCAT GGAC-----C
chrU_5679045          ATCTTAGGGG AACTCAGAAG TGATTTCTTT GAGCAAAAAT TATACTCCAT GGACTAC--C
Amieu_8               GTCTTAAGGG AACTCAGAAG TGATTTCTTT GAGCAAAAAT TATCCTCCAT GGACTACACC
Valence_14            GTCTTAAGGG AACTCAGAAG TGATTTCTTT GAGCAAAAAT TATCCTCCAT GGACTACACC
Valence_2             GTCTTAAGGG AACTCAGAAG TGATTTCTTT GAGCAAAAAT TATCCTCCAT GGACTACACC
Valence_4             GTCTTAAGGG AACTCAGAAG TGATTTCTTT GAGCAAAAAT TATCCTCCAT GGACTACACC
Zimbabwe_76           ATCTTAAGGG AACTCAGAAG TGATTTCTTT GAGCAAAAAT TATCCTCCAT GGACTACACC
Zimbabwe_712          ATCTTAAGGG AACTCAGAAG TGATTTCTTT GAGCAAAAAT TATCCTCCAT GGACTACACC
chrU_5950518          ATCT------ ---------- ---------- ---------- ---------- ----------
chrU_6720385          ATCTTAAGGG AACTCAGAAG TGATTTCTTT GAGCAAAAAT TATCCGCCAT GGACTACACC
Madeira_3’2           ATCTTAAGGG AACTCAGAAG TGATTACTTT GAGCAAAAAT T--------T G----CCTGC
chrX_16602314         ATCTTAAGGG AACTCAGAAG TGATTTCTTT GAGCAAAAAT TATCCTCCAT GGACTAAACC
```

```
                     361
chr3R_1506433_ORF2   GTTGATGCAA ACTATTCGCT GTGGAAGTGC ACAAAAGCCC TTAAACGACA ACCACTTCGA
Canberra_82          ---------- ---------- ---------- ---------- ---------- ----------
Zimbabwe_71          ---------- ---------- ---------- ---------- ---------- ----------
Valence_6            ---------- ---------- ---------- ---------- ---------- ----------
Makindu_51           ---------- ---------- ---------- ---------- ---------- ----------
Zimbabwe_79          ---------- ---------- ---------- ---------- ---------- ----------
Zimbabwe_77          ---------- ---------- ---------- ---------- ---------- ----------
Zimbabwe_714         ---------- ---------- ---------- ---------- ---------- ----------
Zimbabwe_715         ---------- ---------- ---------- ---------- ---------- ----------
Papeete_5            ---------- ---------- ---------- ---------- ---------- ----------
Papeete_1            ---------- ---------- ---------- ---------- ---------- ----------
Eden_4’14            ---------- ---------- ---------- ---------- ---------- ----------
Valence_5            ---------- ---------- ---------- ---------- ---------- ----------
Madeira_3’5          ---------- ---------- ---------- ---------- ---------- ----------
Madeira_3’p’1        ---------- ---------- ---------- ---------- ---------- ----------
Madeira__3’10        ---------- ---------- ---------- ---------- ---------- ----------
Canberra_810         ---------- ---------- ---------- ---------- ---------- ----------
Eden_4’9             ---------- ---------- ---------- ---------- ---------- ----------
Eden_4’4             ---------- ---------- ---------- ---------- ---------- ----------
Eden_4’3             ---------- ---------- ---------- ---------- ---------- ----------
Eden_4’11            ---------- ---------- ---------- ---------- ---------- ----------
Eden_4’12            ---------- ---------- ---------- ---------- ---------- ----------
Amieu_1              ---------- ---------- ---------- ---------- ---------- ----------
chrU_5384045         ---------- ---------- ---------- ---------- ---------- ----------
Zimbabwe_72          GTTGATGCAA ACTATTCGCT GTGGAAGTGC ACAAAAGCCC TTAAACGACA ACCACTTCGA
chrU_3975907         GTTGATGCAA ACTATTTGCT GTGGAAGTGC ACAAAAGCGC TTAAACGACA ACCACTTCGA
Canberra_87          GTTGATGCAA ACTATTCGCT GTGGAAGTGC ACAAAAGCCC TTAAACGACA ACCACTTCGA
Canberra_88          GTTGATGCAA ACTATTCGCT GTGGAAGTGC ACAAAAGCCC TTAAACGACA ACCACTTCGA
Canberra_83          GTTGATGCAA ACTATTTGCT GTGGAAGTGC ACAAAAGCGC TTAAACGACA ACCACTTCGA
Valence_13           GTTGATGCAA ACTATTCGCT GTGGAAGTGC ACAAAAGCCC TTAAACGACA ACCACTTCGA
Papeete_12           GTTGATGCAA ACTATTCGCT GTGGAAGTGC ACAAAAGCCC TTAAACGACA ACCACTTCGA
Zimbabwe_710         GTTGATGCAA ACTATTCGCT GTGGAAGTGC ACAAAAGCCC TTAAACGACA ACCACTTCGA
Papeete_6            GTTGATGCAA ACTATTTGCT GTGGAAGTGC ACAAAAGCGC TTAAACGACA ACCACTTCGA
Canberra_815         GTTGATGCAA ACTATTCGCT GTGGAAGTGC ACAAAAGCCC TTAAACGACA ACCACTTCGA
Papeete_2            GTTGATGCAA ACTATTCGCT GTGGAAGTGC GCAAAAGCCC TTAAACGACA ACCACTTCGA
Brazzaville_1        GTTGATGCAA ACTATTCGCT GTGGAAGTGC ACAAAAGCCC TTAAACGACA ACCACTTCGA
Madeira_3’9          GTTGATGCAA ACTATTCGCT GTGGAAGTGC ACAAAAGCCC TTAAACGACA ACCACTTCGA
Madeira_3’8          GTTGATGCAA ACTATTCGCT GTGGAAGTGC ACAAAAGCCC TTAAACGACA ACCACTTCGA
Madeira_3’7          GTTGATGTAA ACTATTCGCT GTGGGAGTGC ACAAAAGCCC TTAAACGACA ACCACTTCGA
Eden_4’13            GTTGATGCAA ACTATTCGCT GTGGAAGTGC ACAAAAGCCC TTAAACGACA ACCACTTCGA
Madeira_4’10         GTTGATGCAA ACTATTTGCT GTGGAAGTGC ACAAAAGCGC TTAAACGACA ACCACTTCGA
Amieu_68             GTTGATGCAA ACTATTCGCT GTGGAAGTGC ACAAAAGCCC TTAAACGACA ACCACTTCGA
Amieu_69             GTTGATGCAA ACTATTCGCT GTGGAAGCGC ACAAAAGCCC TTAAACGACA ACCACTTCGA
Amieu_4              GTTGATGCAA ACTATTCGCT GTGGAAGTGC ACAAAAGCGC TCAAACGACA ACCACTTCGA
Makindu_5’4          TTGGATGCAA ACTATTCGCT GTGGAAGTGC ACAAAAGCGC TTAAACTACA ACCACCTCGA
Papeete_10           TTGGATGCAA ACTATTCGCT GTGGAAGTGC ACAAAAGCGC TTAAACTACA ACCACCTCGA
Canberra_84          TTGGATGCAA ACTATTCGCT GTGGAAGTGC ACAAAAGCGC TTAAACTACA ACCACCTCGA
Cann                 GTTGATGAAA ACTATTCGCT GTGGAAGTGC ACAAAAGCGC TTAAACAACA ACCACTTCGA
Amieu_61             GTTGATGCAA ACTATTCGCT GTGGAAGTGC ACAAAAGCCC TTAAACGACA ACCACTTCGA
Amieu_62             GTTGATGCAA ACTATTCGCT GTGGAAGTGC ACAAAAGCCC TTAAACGACA ACCACTTCGA
Amieu_65             GTTGATGCAA ACTATTCGCT GTGGAAGTGC ACAAAAGCCC TTAAACGACA ACCACTTCGA
chrU_10862415        GTTGATGCAA ACTATTCGCT GTGGAAGTGC ACAAAAGCCC TTAAACGACA ACCACTTCGA
chrU_13548152        -------CAA ACTATTCGCT GTGGAAGTGC ACAAAAGCGC TTAAACGACA ACTACTTCGA
chrU_1677297         GTTGATGCAA ACTATTCGCT GTGGAAGTGC ACAAAAGCGC TTAAACGACA ACCACTTCGA
chrU_2316095         GTTGATGCAA ACTATTCGCT GTGGAAGTGC ACAAAAGCGC TTAAACGACA ACCACTTTGA
chrU_5679045         GTTGATGAAA ACTATTCGCT GTGGAAGTGC ACAAAAGCGC TTAAACGACA ACCACTTCGA
Amieu_8              GTTGATGCAA ACTATTCGCT GTGGAAGTGC ACAAAAGCCC TTAAACGACA ACCACTTCGA
Valence_14           GTTGATGCAA ACTATTCGCT GTGGAAGTGC ACAAAAGCCC TTAAACGACA ACCACTTCGA
Valence_2            GTTGATGCAA ACTATTCGCT GTGGAAGTGC ACAAAAGCCC TTAAACGACA ACCACTTCGA
Valence_4            GTTGATGCAA ACTATTCGCT GTGGAAGTGC ACAAAAGCCC TTAAACGACA ACCACTTCGA
Zimbabwe_76          GTTGATGCAA ACTATTTGCT GTGGAAGTGC ACAAAAGCGC TTAAACGACA ACCACTTCGA
Zimbabwe_712         GTTGATGAAA ACTATTCGCT GTGGAAGTGC ACAAAAGCGC TTAAACAACA ACCACTTCGA
chrU_5950518         -------CAA ACTATTCGCT GTGGAAGTGC ACAAAAGCGC TTAAACGACA ACTACTTCGA
chrU_6720385         GTTGATGCAA ACTATTCGCT GTGGAAGTGC ACAAAAGAGA TTGAGCGACA ACCACTTCGA
Madeira_3’2          TTGGATGCAA ACTATTCGCT GTGGAAGTGC ACAAAAGCGC TTAAACTACA ACCACCTCGA
chrX_16602314        GTTGATGCAA ACTATTCGCT GTGGAAGTGC ACAAAAGCGC TTAAACGACA ACCACTTCGA
```

```
                           421
chr3R_1506433_ORF2  TGGGTACCCG TACGCTGTCC AGGTGGGGAA TTTGCAAAAA CTGAAG--TG GAACAGGCTA
Canberra_82         ---------- ---------- ---------- ---------- ---------- ----------
Zimbabwe_71         ---------- ---------- ---------- ---------- ---------- ----------
Valence_6           ---------- ---------- ---------- ---------- ---------- ----------
Makindu_51          ---------- ---------- ---------- ---------- ---------- ----------
Zimbabwe_79         ---------- ---------- ---------- ---------- ---------- ----------
Zimbabwe_77         ---------- ---------- ---------- ---------- ---------- ----------
Zimbabwe_714        ---------- ---------- ---------- ---------- ---------- ----------
Zimbabwe_715        ---------- ---------- ---------- ---------- ---------- ----------
Papeete_5           ---------- ---------- ---------- ---------- ---------- ----------
Papeete_1           ---------- ---------- ---------- ---------- ---------- ----------
Eden_4'14           ---------- ---------- ---------- ---------- ---------- ----------
Valence_5           ---------- ---------- ---------- ---------- ---------- ----------
Madeira_3'5         ---------- ---------- ---------- ---------- ---------- ----------
Madeira_3'p'1       ---------- ---------- ---------- ---------- ---------- ----------
Madeira__3'10       ---------- ---------- ---------- ---------- ---------- ----------
Canberra_810        ---------- ---------- ---------- ---------- ---------- ----------
Eden_4'9            ---------- ---------- ---------- ---------- ---------- ----------
Eden_4'4            ---------- ---------- ---------- ---------- ---------- ----------
Eden_4'3            ---------- ---------- ---------- ---------- ---------- ----------
Eden_4'11           ---------- ---------- ---------- ---------- ---------- ----------
Eden_4'12           ---------- ---------- ---------- ---------- ---------- ----------
Amieu_1             ---------- ---------- ---------- ---------- ---------- ----------
chrU_5384045        ---------- ---------- ---------- ---------- ---------- ----------
Zimbabwe_72         TGGGTACCCG TACGCTGTCC AGGTGGGGAA TTTGCAAAAA CTGAAG--TG GAACAGGCTA
chrU_3975907        TGGGTACCCG TACGCTGTCC AGGTGGGGAA TTTGCAAAAA CTGAAG--TG GAACAGGCTA
Canberra_87         TGGGTACCCG TACGCTGTCC AGGTGGGGAA TTTGCAAAAA CTGAAG--TG GAACAGGCTA
Canberra_88         TGGGTACCCG TACGCTGTCC AGGTGGGGAA TTTGCAAAAA CTGAAG--TG GAACAGGCTA
Canberra_83         TGGGTACCCG TACGCTGTCC AGGTGGGGAA TTTGCAAAAA CTGAAG--TG GAACAGGCTA
Valence_13          TGGGTACCCG TACGCTGTCC AGGTGGGGAA TTTGCAAAAA CTGATG--TG GAACAGGCTA
Papeete_12          TGGGTACCCG TACGCTGTCC AGGTGGGGAA TTTGCAAAGA CTGAAG--TG GAACAGGCTA
Zimbabwe_710        TGGGTACCCG TACGCTGTCC AGGTGGGGAA TTTGCAAAAA CTGAAG--TG GAACAGGCTA
Papeete_6           TGGGTACCCG TACGCTGTCC AGGTGGGGAA TTTGCAAAAA CTGAAG--TG GAACAGGCTA
Canberra_815        TGGGTACCCG TACGCTGTCC AGGTGGGGAA TTTGCAAAAA CTGAAG--TG GAACAGGCTA
Papeete_2           TGGGTACCCG TACGCTGTCC AGGTGGGGAA TTTGCAAAAA CTGAAG--TG GAACAGGCTA
Brazzaville_1       TGGGTACCCG TACGCTGTCC AGGTGGGGAA TTTGCAAAAA CTGAAG--TG GAACAGGCTA
Madeira_3'9         TGGGTACCCG TACGCTGTCC AGGTGGGGAA TCTGCAAAAA CTGAAG--TG GAACAGGCTA
Madeira_3'8         TGGGTACCCG TACGCTGTCC AGGTGGGGAA TTTGCAAAAA ATGAAG--TG GAACAGGCTA
Madeira_3'7         TGGGTACCCG TACGCTGTCC AGGTGGGGAA TTTGCAAAAA CTGAAG--TG GAACAGGCTA
Eden_4'13           TGGGTACCCG TACGCTGTCC AGGTGGGGAA TTTGCAAAAA CTGAAG--TG GAACAGGCTA
Madeira_4'10        TGGGTACCCG TACGCTGTCC AGGTGGGGAA TTTGCAAAAA CTGAAG--TG GAACAGGCTA
Amieu_68            TGGGTACCCG TACGCTGTCC AGGTGGGGAA TTTGCAAAAA CTGAAG--TG GAACAGGCTA
Amieu_69            TGGGTACCCG TACGCTGTCC AGGTGGGGAA TTTGCAAAAA CTGAAG--TG GAACAGGCTA
Amieu_4             TGGGTACCCG TACGCTGTCC AGGTGGGGAA TTTGCAAAAA CTGAAG--TG GAACAGGCTA
Makindu_5'4         TGGGTACCCG TACGCTGTCC AGGTGGGGAA TTTGCAAAAG CTGAAGGCTG GAACAGGCTA
Papeete_10          TGGGTACCCG TACGCTGTCC AGGTGGGGAA TTTGCAAAAG CTGAAGGCT- ----------
Canberra_84         TGGGTACCCG TACGCTGTCC AGGTGGGGAA TTTGCAAAAG CTGAAG---- ------GCT-
Cann                TGGGTACCCG TACGCTGTCC AGGTGGGGAA TTTGCAAAAA CTGAAG--TG GAACAGGCTT
Amieu_61            TGGGTACCCG TACGCTGTCC AGGTGGGGAA TTTGCAAAAA CTGAAG--TG GAACAGGCTA
Amieu_62            TGGGTACCCG TACGCTGTCC AGGTGGGGAA TTTGCAAAAA CTGAAG--TG GAACAGGCTA
Amieu_65            TGGGTACCCG TACGCTGTCC AGGTGGGGAA TTTGCAAAAA CTGAAG--TG GAACAGGCTA
chrU_10862415       TGGGTACCCG TACGCTGTCC AGGTGGGGAA TTTGCAAAAA CTGAAG--TG GAACAGGCTA
chrU_13548152       TGGGTACCCG CACGCTGTCC AGGTGGGGAA TTTGCAAAAA CTGAAG--TG GAACAGGCTA
chrU_1677297        TGGGTACCCG TACGCTGTCC AGGTGGGGAA TTTGCAAAAA CTGAAG--TG GAACAAGCTA
chrU_2316095        TGGGTACCAG TACGCTGTCC AGGTGGGGAA TTTGCAAAAA CTGAAG--TA AAACAGGCTA
chrU_5679045        TGGGTACCCG TACTCTGTCA AGGTGGGGAA TTTGTAAAAA CTGAAG--TG GAACAGGCTA
Amieu_8             TGGGTACCCG TACGCTGTCC AGGTGGGGAA TTTGCAAAAA CTGAAG--TG GAACAGGCTA
Valence_14          TGGGTACCCG TACGCTGTCC AGGTGGGGAA TTTGCAAAAA CTGAAG--TG GAACAGGCTA
Valence_2           TGGGTACCCG TACGCTGTCC AGGTGGGGAA TTTGCAAAAA CTGAAG--TG GAACAGGCTA
Valence_4           TGGGTACCCG TACGCTGTCC AGGTGGGGAA TTTGCAAAAA CTGAAG--TG GAACAGGCTA
Zimbabwe_76         TGGGTACCCG TACGCTGTCC AGGCGGGGAA TTTGCAAAAA CTGAAG--TG GAACAGGCTA
Zimbabwe_712        TGGGTACCCG TACGCTGTCC AGGTGGGGAA TTTGCAAAAA CTGAAG--TG GAACAGGCTT
chrU_5950518        TGGGTACCCG CACGCTGTAC AGGTGGGGAA TTTGCAAAAA CTGAAG--TG GAACAAGCTA
chrU_6720385        TGGGTACCCA TACGCTGTCC AGGTGGGGAA TTTGCAAAAA ATGAAG--TG GAACAGGCTA
Madeira_3'2         TGGGTACCCG TACGCTGTCC AGGTGGGGAA TTTGCAAAAG CTGAAGGCTG GAACAGGCTA
chrX_16602314       TGGGTACCCG TACGCTGTCC AGGTGGGGAA TTTGCAAAAA CTGAAG--TG GAACAGGCTA
```

```
                  481
chr3R_1506433_ORF2 ATGCATTCGG CTTCCACCTA GAGGATCGCT TCACTCCTTA CGACTTCGCC ACGACAGAAC
Canberra_82        ---------- ---------- ---------- ---------- ---------- ----------
Zimbabwe_71        ---------- ---------- ---------- ---------- ---------- ----------
Valence_6          ---------- ---------- ---------- ---------- ---------- ----------
Makindu_51         ---------- ---------- ---------- ---------- ---------- ----------
Zimbabwe_79        ---------- ---------- ---------- ---------- ---------- ----------
Zimbabwe_77        ---------- ---------- ---------- ---------- ---------- ----------
Zimbabwe_714       ---------- ---------- ---------- ---------- ---------- ----------
Zimbabwe_715       ---------- ---------- ---------- ---------- ---------- ----------
Papeete_5          ---------- ---------- ---------- ---------- ---------- ----------
Papeete_1          ---------- ---------- ---------- ---------- ---------- ----------
Eden_4’14          ---------- ---------- ---------- ---------- ---------- ----------
Valence_5          ---------- ---------- ---------- ---------- ---------- ----------
Madeira_3’5        ---------- ---------- ---------- ---------- ---------- ----------
Madeira_3’p’1      ---------- ---------- ---------- ---------- ---------- ----------
Madeira__3’10      ---------- ---------- ---------- ---------- ---------- ----------
Canberra_810       ---------- ---------- ---------- ---------- ---------- ----------
Eden_4’9           ---------- ---------- ---------- ---------- ---------- ----------
Eden_4’4           ---------- ---------- ---------- ---------- ---------- ----------
Eden_4’3           ---------- ---------- ---------- ---------- ---------- ----------
Eden_4’11          ---------- ---------- ---------- ---------- ---------- ----------
Eden_4’12          ---------- ---------- ---------- ---------- ---------- ----------
Amieu_1            ---------- ---------- ---------- ---------- ---------- ----------
chrU_5384045       ---------- ---------- ---------- ---------- ---------- ----------
Zimbabwe_72        ATGCATTCGG CTTCCACCTA GAGGATCGCT TCACTCCTTA CGACTTCGCC ACGACAGAAC
chrU_3975907       ATGCATTCGG CTTCCACCTA GAGGATCGCT TCACTCCTTA CGACTTCGC- ACTACAGAAC
Canberra_87        ATGCATTCGG CTTCCACCTA GAGGATCGCT TCACTCCTTA CGACTTCGCC ACGACAGAAC
Canberra_88        ATGCATTCGG CTTCCACCTA GAGGATCGCT TCACTCCTTA CGACTTCGCC ACGACAGAAC
Canberra_83        ATGCATTCGG CTTCCACCTA GAGGATCGCT TCACTCCTTA CGACCTCGC- ACTACAGAAC
Valence_13         ATGCATTCGG CTTCCACCTA GAGGATCGCT TCACTCCTTA CGACTTCGCC ACGACAGAAC
Papeete_12         ATGCATTCGG CTTCCACCTA GAGGATCGCT TCACTCCTTA CGACTTCGCC ACGACAGAAC
Zimbabwe_710       ATGCATTCGG CTTCCACCTA GAGGATCGCT TCACTCCTTA CGACTTCGCC ACGACAGAAC
Papeete_6          ATGCATTCGG CTTCCACCTA GAGGATCGCT TCACTCCTTA CGACTTCGC- ACTACAGAAC
Canberra_815       ATGCATTCGG CTTCCACCTA GACGATCGCT TCACTCCTTA CGACTTCGCC ACGACAGAAC
Papeete_2          ATGCATTCGG CTTCCACCTA GAGGATCGCT TCACTCCTTA CGACTTCGCC ACGACAGAAC
Brazzaville_1      ATGCATTCGG CTTCCACCTA GAGGATCGCT TCACTCCTTA CGACTTCGCC ACGACAGAAC
Madeira_3’9        ATGCATTCGG CTTCCACCTA GAGGATCGCT TCACTCCTTA CGACTTCGCC ACGACAGAAC
Madeira_3’8        ATGCATTCGG CTTCCACCTA GAGGATCGCT TCACTCCTTA CGACTTCGCC ACGACAGAAC
Madeira_3’7        ATGCATTCGG CTTCCACCTA GAGGATCGCT TCACTCCTTA CGACTTCGCC ACGACAGAAC
Eden_4’13          ATGCATTCGG CTTCCACCTA GAGGATCGCT TCACTCCTTA CGACTTCGCC ACGACAGAAC
Madeira_4’10       ATGCATTCGG CTTCCACCTA GAGGATCGCT TCACTCCTTA CGACTTCGC- ACTACAGAAC
Amieu_68           ATGCATTCGG CTTCCACCTA GAGGATCGCT TCACTCCTTA CGACTTCGCC ACGACAGAAC
Amieu_69           ATGCATTCGG CATCCACCTA GAGGATCGCT TCACTCCTTA CGACTTCGCC ACGACAGAAC
Amieu_4            ATGCATTCGG CTTCCACCTA GAGGATCGCT TCACTCCTTA CGACTTCCCC ACGACAGAAC
Makindu_5’4        ATGCATTCGG CTTCCACCTA GAGGATCGCT TCACTCCTTA CGACTTCGCC ACGACAGAAC
Papeete_10         TTGCATTCGG CTTCCACCTA GAGGATCGCT TCACTCCTTA CGACTTCGCC ACGACAGAAC
Canberra_84        TTGCATTCGG CTTCCACCTA GAGGATCGCT TCACTCCTTA CGACTTCGCC ACGACAGAAC
Cann               ATGCATTCGT CTTCCACCTA GGGTATCGCT TCACTCCTTA CGACTTCGCC ACGACAGAAC
Amieu_61           ATGCATTCGG CTTCCACCTA GAGGATCGCT TCACTCCTTA CGACTTCGCC ACGACAGAAC
Amieu_62           ATGCATTCGG CTTCCACCTA GAGGATCGCT TCACTCCTTA CGACTTCGCC ACGAGAGAAC
Amieu_65           ATGCATTCGG CTTCCACCTA GAGGATCGCT TCACTCCTTA CGACTTCGCC ACGAC-GAAC
chrU_10862415      ATGCATTCGG CTTCCACCTA GAGGATCGCT TCACTCCTTA CGACTTCGCC ACGACAGAAC
chrU_13548152      ATGCATTCGG CTTCCACCTA GAGGATCGCT TCACTCCTTA CGACTTCGCC ACGACAGAAC
chrU_1677297       ATGCATTCGG CTTCCACCTA GAGGATCGCT TCACTCCTTA CGACTTCGCC ACGACAGAAC
chrU_2316095       ATGCAATCGG CTTCCACCTA GAGGATCGCT TTACTCCTTA TGACTTCGCC ACGACTGAAC
chrU_5679045       ATGCATTCGG CTTCCACCTA GAGGATCGCT TCACTCCTTA CGACTTCGCC ACGACAGAAC
Amieu_8            ATGCATTCGG CTTTCACCTA GAGGATCGCT TCACTCCTTA CGACTTCGCC ACGACAGAAC
Valence_14         ATGCATTCGG CTTCCACCTA GAGGATCGCT TCACTCCTTA CGACTTCGCC ACGACAGAAC
Valence_2          ATGCATTCGG CTTCCACCTA GAGGATCGCT TCACTCCTTA CGACTTCGCC ACGACAGAAC
Valence_4          ATGCATTCGG CTTCCACCTA GAGGATCGCT TCACTCCTTA CGACTTCGCC ACGACAGAAC
Zimbabwe_76        ATGCATTCGG CTTCCACCTA GAGGATCGCT TCACTCCTTA CGACTTCGC- ACTACAGAAC
Zimbabwe_712       ATGCATTCGT CTTCCACCTA GGGTATCGCT TCACTCCTTA CGACTTCGCC ACGACAGAAC
chrU_5950518       ATGCATTCGG CTTCCACCTA GAGGATCGCT TCACTCCTTA CGACTTCCCC ACGACAAAAC
chrU_6720385       ATGCATTCGG CTTCCGCCTA GAGGATCGCT TCACTCCTTA CGACTTCGCC ACGACAGAAC
Madeira_3’2        ATGCATTCGG CTTCCACCTA GAGGATCGCT TCACTCCTTA CGACTTCGCC ACGACAGAAC
chrX_16602314      ATGCATTCGG CTTCCACCTA GAGGATCGCT TCACTCCTTA CGACTTCCCC ACGACAGAAC
```

```
                     541
chr3R_1506433_ORF2   AAATAAGAGA GACTCACCAG TACCTACAAA TGCCATTGCA GATGTCTTGG CC-TATTAAG
Canberra_82          ---------- ---------- ---------- ---------- ---------G CC--------
Zimbabwe_71          ---------- ---------- ---------- ---------- ---------G CC--------
Valence_6            ---------- ---------- ---------- ---------- ---------G CC-TATTAAG
Makindu_51           ---------- ---------- ---------- ---------- ---------G CC-TATTAAG
Zimbabwe_79          ---------- ---------- ---------- ---------- ---------G CCCTATTAAG
Zimbabwe_77          ---------- ---------- ---------- ---------- ---------G CC-TATTAAG
Zimbabwe_714         ---------- ---------- ---------- ---------- ---------G CC--------
Zimbabwe_715         ---------- ---------- ---------- ---------- ---------G CC-TATTAAG
Papeete_5            ---------- ---------- ---------- ---------- ---------- ----------
Papeete_1            ---------- ---------- ---------- ---------- ---------- ----------
Eden_4’14            ---------- ---------- ---------- ---------- ---------G CC-TATTAAG
Valence_5            ---------- ---------- ---------- ---------- ---------G CC-TATTAAG
Madeira_3’5          ---------- ---------- ---------- ---------- ---------G CC-TATTAAG
Madeira_3’p’1        ---------- ---------- ---------- ---------- ---------G CC-TATTAAG
Madeira__3’10        ---------- ---------- ---------- ---------- ---------G CC-TATTAAG
Canberra_810         ---------- ---------- ---------- ---------- ---------G CC-TATTAAG
Eden_4’9             ---------- ---------- ---------- ---------- ---------G CC-TATTAAG
Eden_4’4             ---------- ---------- ---------- ---------- ---------G CC-TATTAAG
Eden_4’3             ---------- ---------- ---------- ---------- ---------G CC-TATTAAG
Eden_4’11            ---------- ---------- ---------- ---------- ---------G CC-TATTAAG
Eden_4’12            ---------- ---------- ---------- ---------- ---------G CC-TATTAAG
Amieu_1              ---------- ---------- ---------- ---------- ---------G CC-TATTAAG
chrU_5384045         ---------- ---------- ---------- ---------- ---------G CC-TATTAAG
Zimbabwe_72          AAATAAGAGA GACTCACCAG TACCTACAAA TGCCATTGCA GATGTCTTGG CC-TATTAAG
chrU_3975907         AAATAAGAGA GACTCACCAG TACCTACAAA TGCCATTGCA GATGTCTTGG CC-TATTAAG
Canberra_87          AAATAAGAGA GACTCACCAG TACCTACAAA TGCCATTGCA GATGTCTTGG CC-TATTAAG
Canberra_88          AAATAAGAGA GACTCACCAG TACCTACAAA TGCCATTGCA GATGTCTTGG CC-TATTAAG
Canberra_83          AAATAAGAGA GACTCACCAG TACCTACAAA TGCCATTGCA GATGTCTTGG CC-TATTAAG
Valence_13           AAATAAGAGA GACTCACCAG TACCTACAAA TGCCATTGCA GATGTCTTGG CC-TATTAAG
Papeete_12           AAATAAGAGA GACTCACCAG TACCTACAAA TGCCATTGCA GATGTCTTGG CC-TATTAAG
Zimbabwe_710         AAATAAGAGA GACTCACCAG TACCTACAAA TGCCATTACA GATGTCTTGG CC-TATTAAG
Papeete_6            AAATAAGAGA GACTCACCAG TACCTACAAA TGCCATTGCA GATGTCTTGG CC-TATTAAG
Canberra_815         AAATAAGAGA GACTCACCAG TACCTACAAA TGCCATTGCA GATGTCTTGG CC-TATTAAG
Papeete_2            AAATAAGAGA GACTCACCAG TACCTACAAA TGCCATTGCA GATGTCTTGG CC-TATTAAG
Brazzaville_1        AAATAAGAGA GACTCACCAG TACCTACAAA TGCCATTGCA GATGTCTTGG CC-TATTAAG
Madeira_3’9          AAATAAGAGA GACTCACCAG TACCTACAAA TGCCATTGCA GATGTCTTGG CC-TATTAAG
Madeira_3’8          AAATAAGAGA GACTCACCAG TACCTACAAA TGCCATTGCA GATGTCTTGG CC-TATTAAG
Madeira_3’7          AAATAAGAGA GACTCACCAG TACCTACAAA TGCCATTGCA GATGTCTTGG CC-TATTAAG
Eden_4’13            AAATAAGAGA GACTCACCAG TACCTACAAA TGCCATTGCA GATGTCTTGG CC-TATTAAG
Madeira_4’10         AAATAAGAGA GACTCACCAG TACCTACAAA TGCCATTGCA GATGTCTTGG CC-TATTAAG
Amieu_68             AAATAAGAGA GACTTACCAG TACCTACAAA TGCCATTGCA GATGTCTTGG CC-TATTAAG
Amieu_69             AAATAAGAGA GACTCACCAG TACCTACAAA TGCCATTGCA GATGTCTTGG CC-TATTAAG
Amieu_4              AAATAAGAGA GACTCACCAG TACCTACAAA TGCCATTGCA GATGTCTTGG CC-TATTAAG
Makindu_5’4          AAATAAGAGA GACTCATCAG TACCTACAAA TGCCATTGCA GATGTCT--- ----------
Papeete_10           AAATAAGAGA GACTCATCAG TACCTACAAA TGCCATTGCA GATGTCTTGG CC-TTTTAAG
Canberra_84          AAATAAGAGA GACTCATCAG TACCTACAAA TGCCATTGCA GATGTCTTGG CC-TTTTAAG
Cann                 AAATAAGAGA GAC----CAG TACCTACAAA TGACATTGCA GATGTCTTGG CC-TATTAAG
Amieu_61             AAATAAGAGA GACTCACCAG TACCTACAAA TGCCATTGCA GATGTCTTGG CC-TATTAAG
Amieu_62             AAATAAGAGA GACTCACCAG TACCTACAAA TGCCATTACA GATGTCTTGG CC-TATTAAG
Amieu_65             AAATAAGAGG GACTCACCAG TACCTACAAA TGCCATTGCA GATGTCTTGG CC-TATTAAG
chrU_10862415        AAATAAGAGA GACTCACCAG TACCCACAAA TGCCATTGCA GATGTCTTGG CC-TATTAAG
chrU_13548152        AAATAAGAGA GACTCACCAG TACCTACAAA TGCCATTACA GATGTCTTGG CC-TATTAAG
chrU_1677297         AAATAAGAGA GACTCACCAG TACCTACAAA TGCCATTGCA GATGTCTTAG CC-TATTAAG
chrU_2316095         AAATAAGAGA GACTCACCAG TACCTACAAA TGCCATTGCA GATGTCTTGG CC-TATTAAG
chrU_5679045         AAATAAGAGA GACTCATCAG TACCTACAAA TGTCATTGCA GATGTCTTGG CC-TATTAAG
Amieu_8              AAATAAGAGA GACTCACCAG TACCTACAAA TGCCATTGCA GATGTCTTGG CC-TATTAAG
Valence_14           AAATAAGAGA GACTCACCAG TACCTACAAA TGCCATTGCA GATGTCTTGG CC-TATTAAG
Valence_2            AAATAAGAGA GACTC----- ---------- ---------- ---------- ----------
Valence_4            AAATAAGAGA GACTCACCAG TACCTACAAA TGCCATTGCA GATGTCTTGG CC-TATTAAG
Zimbabwe_76          AAATAAGAGA GACTCACCAG TACCTACAAA TGCCATTGCA GATGTCTTGG CC-TATTAAG
Zimbabwe_712         AAATAAGAGA GAC----CAG TACCTACAAA TGACATTGCA GATGTCTTGG CC-TATTAAG
chrU_5950518         AAATAAAAAA GACTCACCA- ---------- ---------- ---------- ----------
chrU_6720385         AAATAAGAGA GACTCACCAG TACCTACAAA TGCCATTGCA GATGTCTTGG CC-TATTAAG
Madeira_3’2          AAATAAGAGA GACTCATCAG TACCTACAAA TGCCATTGCA GATGTCC--- ----------
chrX_16602314        AAATAAGAGA GACTCACCAG TACCTACAAA TGCCATTGCA GATGTCTTGG CC-TATTAAG
```

```
                          601
chr3R_1506433_ORF2  CC-AATAAGG ATAGAAGAAA TCCTTGAAAT AAT
Canberra_82         ---------- ---------- ---------- ---
Zimbabwe_71         ---------- ---------- ---------- ---
Valence_6           CC-AATAAGG ATACAAGAA- ---------- ---
Makindu_51          CC-AATAAGG ATACAAGAAA TC-------- ---
Zimbabwe_79         CCCAATAAGG ATACAAGAAA TCCTTGAAAT AAT
Zimbabwe_77         CC-AATAAGG ATACAAGAAA TCCTTGAAAT AAT
Zimbabwe_714        ---------- ---------- ---------- ---
Zimbabwe_715        CC-AATAAGG ATACAAGAAA TCCT------ ---
Papeete_5           ---------- ---------- ---------- ---
Papeete_1           ---------- ---------- ---------- ---
Eden_4’14           CC-AATAAGG ATACAAGAA- ---------- ---
Valence_5           CC-AATAAGG ATACAAGAA- ---------- ---
Madeira_3’5         CC-AATAAGG ATACAAGAA- ---------- ---
Madeira_3’p’1       CC-AATAAGG ATACAAGAA- ---------- ---
Madeira__3’10       CC-AATAAGG ATACAAGAA- ---------- ---
Canberra_810        CC-AATAAGG ATACAAGAAA TCCT------ ---
Eden_4’9            CC-AATAAGG ATACAAGAA- ---------- ---
Eden_4’4            CC-AATAAGG ATACAAGAA- ---------- ---
Eden_4’3            CC-AATAAGG ATACAAGAA- ---------- ---
Eden_4’11           CC-AATAAGG ATACAAGAA- ---------- ---
Eden_4’12           CC-AATAAGG ATACAAGAAA TC-------- ---
Amieu_1             CC-AATAAGG ATACAAGAA- ---------- ---
chrU_5384045        CC-AATAAGG ATACAAGAAA TCCTTGAAAT AAT
Zimbabwe_72         CC-AATAAGG ATAGAAGAAA TCCTTGAAAT A--
chrU_3975907        CC-AATAAGG ATAGAAGAAA TCCTTGAAAT AAT
Canberra_87         CC-AATAAGG ATAGAAGAAA TCCT------ ---
Canberra_88         CC-AATAAGG ATAGAAGAAA TCCT------ ---
Canberra_83         CC-AATAAGG ATAGAAGAAA TCCT------ ---
Valence_13          CC-AATAAGG ATAGAAGAA- ---------- ---
Papeete_12          CC-AATAAGG ATAGAAGAA- ---------- ---
Zimbabwe_710        CC-AATAAGG ATAGAAGAAA TCCT------ ---
Papeete_6           CC-AATAAGG ATAGAAGAA- ---------- ---
Canberra_815        CC-AATAAGG ATAGAAGAAA TCCT------ ---
Papeete_2           CC-AATAAGG ATAGAAGAA- ---------- ---
Brazzaville_1       CC-AATAAGG ATAGAAGAA- ---------- ---
Madeira_3’9         CC-AATAAGG ATAGAAGAA- ---------- ---
Madeira_3’8         CC-AATAAGG ATAGAAGAA- ---------- ---
Madeira_3’7         CC-AATAAGG ATAGAAGAAA ---------- ---
Eden_4’13           CC-AATAAGG ATAGAAGAA- ---------- ---
Madeira_4’10        CC-AATAAGG ATAGAAGAA- ---------- ---
Amieu_68            CC-AATAAGG ATAGAAGAA- ---------- ---
Amieu_69            CC-AATAAGG ATAGAAGAA- ---------- ---
Amieu_4             CC-AATAAGG ATAGTAGAA- ---------- ---
Makindu_5’4         ---------- ---------- ---------- ---
Papeete_10          CC-AATAAGG ATAGAAG--- ---------- ---
Canberra_84         CC-AATAAGG ATAGAAGAAA TCC------- ---
Cann                CC-AATAATG ATAGAAGAA- ---------- ---
Amieu_61            CC-AATAAGG ATAGAAGAAA TCCT------ ---
Amieu_62            CC-A------ ---------- ---------- ---
Amieu_65            CC-AATAAGG ATAGAAGAA- ---------- ---
chrU_10862415       CC-AATAAGG ATAGAAGAAA TCCTTGAAAT AAT
chrU_13548152       CC-AATAGCG ATAGAAGAAA TCCTTGAAAT AAT
chrU_1677297        CC-AATAAGG ATAGAAGAAA TCCTTGAAAT AAT
chrU_2316095        CC-AATAAGG ATAGAAGAAT TATCTGAAAT AAT
chrU_5679045        CC-AATA--- ---GAAGAAA TCCTTGAAAT AAT
Amieu_8             CC-AATAAGG ATAGAAGAAA ---------- ---
Valence_14          CC-AATAAGG ATAGAAGAA- ---------- ---
Valence_2           ---------- ---------- ---------- ---
Valence_4           CC-AATAAGG ATAGAAGAAA ---------- ---
Zimbabwe_76         CC-AATAAGG ATAGAAGAAA TCC------- ---
Zimbabwe_712        CC-AATAATG ATAGAAGAAA TCCTTGAAAT AAT
chrU_5950518        ---------- ---------- ---------- ---
chrU_6720385        CC-AATAAGG ATAGAAGAAA TCCTTGAAAT AAT
Madeira_3’2         ---------- ---------- ---------- ---
chrX_16602314       CC-AATAAGG ATAGTAGAAA TCCTTGAAAT AAT
```
